# Interference with mitochondrial metabolism could serve as a potential therapeutic strategy for advanced prostate cancer

**DOI:** 10.1371/journal.pone.0290753

**Published:** 2024-04-10

**Authors:** Chuang Wu, Huihuang Zhu, Yang Zhang, Li Ding, Junqi Wang

**Affiliations:** 1 Department of Urology, Jiangsu Province Geriatric Hospital, Nanjing, Jiangsu, China; 2 Department of Urology, The Affiliated Hospital of Xuzhou Medical University, Xuzhou, Jiangsu, China; Northern Institute for Cancer Research, UNITED KINGDOM

## Abstract

Metabolic reprogramming has been defined as a hallmark of malignancies. Prior studies have focused on the single nucleotide polymorphism (SNP) of POLG2 gene, which is reportedly responsible for encoding mitochondrial DNA genes and is implicated in the material and energy metabolism of tumor cells, whereas its function in prostate cancer has been elusive. Gene expression profile matrix and clinical information were downloaded from TCGA (The Cancer Genome Atlas) data portal, and GSE3325 and GSE8511 were retrieved from GEO (Gene Expression Omnibus) database. We conducted analysis of the relative expression of POLG2, clinical characterization, survival analysis, GO / KEGG and GSEA (Gene Set Enrichment Analysis) enrichment analysis in R and employed STRING portal to acquaint ourselves with the protein-protein interaction (PPI). IHC (Immunohistochemical) profiles of POLG2 protein between normal and cancerous tissues were consulted via HPA (Human protein atlas) database and the immunohistochemical POLG2 were verified between para-cancerous and cancerous tissues in tissue array. At the cellular level, Mitochondrial dysfunction assay, DNA synthesis test, wound healing assay, and invasion assay were implemented to further validate the phenotype of POLG2 knockdown in PCa cell lines. RT-qPCR and western blotting were routinely adopted to verify variations of molecular expression within epithelial mesenchymal transition (EMT). Results showed that POLG2 was over-expressed in most cancer types, and the over-expression of POLG2 was correlated with PCa progression and suggested poor OS (Overall Survival) and PFI (Progress Free Interval). Multivariate analysis showed that POLG2 might be an independent prognostic factor of prostate cancer. We also performed GO/KEGG, GSEA analysis, co-expression genes, and PPI, and observed the metabolism-related gene alterations in PCa. Furthermore, we verified that POLG2 knockdown had an inhibitory effect on mitochondrial function, proliferation, cell motility, and invasion, we affirmed POLG2 could affect the prognosis of advanced prostate cancer via EMT. In summary, our findings indicate that over-expressed POLG2 renders poor prognosis in advanced prostate cancer. This disadvantageous factor can serve as a potential indicator, making it possible to target mitochondrial metabolism to treat advanced prostate cancer.

## Background

It was estimated that there were 1.4 million new cases and 375,000 deaths of prostate cancer in 2020 worldwide. Prostate cancer had been the second most common malignancy and ranked as the fifth-leading cause of cancer deaths for men [1]. ADT (Androgen deprivation therapy) is an effective first-line regime for patients with advanced prostate cancer. Despite good initial responses, the recurrence of CRPC is common [2]. The patients gradually lose their responses, leading to the progression of mCRPC (metastatic castration-resistant prostate cancer), with the Five-Year Relative Survival Rate declining from 99% to only 30% [3]. The regimens for patients with CRPC typically include high-affinity AR antagonists like enzalutamide and the androgen synthesis inhibitor AA (abiraterone acetate) [4–6]. Hopefully, the discovery of the correlation between molecular background and clinical outcomes and regimens to enhance response for tumor progression would bring a prospect for patients with prostate cancers [7]. Nonetheless, the genetic and molecular mechanisms contributory to the pathogenesis and progression of prostate cancer still remain masked [8]. Accordingly, the quest for new specific molecular biomarkers for the therapeutics and prognosis of PRAD (prostate adenocarcinoma) is of great urgency.

Metabolism reprogramming is a hallmark of malignant tumors, with profound metabolic changes contributing to the maintenance of the survival and proliferation of cancer cells [9]. As early as 1927, **Warburg et al.** first described the characteristics of abnormal energy metabolism of cancer cells, which was termed as "aerobic glycolysis", even in the presence of oxygen. However, emerging evidence has demonstrated that certain types of cancer cells rely heavily on the tricarboxylic acid cycle for energy consumption and biosynthesis, such as nucleotides, lipids, and proteins indispensable for cell division [10]. Normally, under regulation of AR signaling, prostatic epithelial cells utilize glucose and aspartate to generate citrate via glycolysis. During this process, prostate epithelial cells are responsible for the elevated levels of mitochondrial zinc via the cellular accumulation of zinc, which inhibits m-aconitase activity and citric acid aerobic oxidation [11]. On the contrary, malignant prostate tissues hardly retain the high levels of citric acid and zinc unique to the normal adjacent area. In the case of declined zinc levels in malignant cells, m-aconitase activity is no longer inhibited, eventually leading to the influx of citric acid into mitochondria and generation of ATP via oxidative phosphorylation to sustain the biosynthesis and proliferation of PCa cells [12, 13].

POLG2 on chromosome 17q, the gene encoding the p55 accessory subunit of pol γ, is required to maintain the genetic integrity of the 16,569-bp human mtDNA (mitochondrial genome) [14, 15]. POLG2 is characterized by fortification of the interaction with DNA catalytic subunit POLGA and prevention of the separation of pol γ from templates during DNA replication [16]. With the onset of DNA replication, the distal POLG2 subunit accelerates the nucleotide incorporation process and may play a role in initiating mitochondrial DNA replication. POLG2 subunit is characteristic of enhancing DNA binding, catalysis, and whole enzyme processing [17]. Catalytic subunit and accessory subunit have a remarkably high affinity for DNA. Kinetic analysis indicates that their affinity for primer terminals has increased by two orders of magnitude and plays a role in mitochondrial DNA repair and replication [18].

Meanwhile, there is evidence that the integrity of the mitochondrial DNA polymerase determines the DNA components of the mitochondrial nucleoids in human cultured cells [19]. Deletion, depletion of mitochondrial DNA and accumulation of locus mutations render the inability to sustain the genetic integrity of the mitochondrial genome and will eventually impair oxidative phosphorylation, leading to MDS(mitochondrial depletion syndrome) [20]. Mutations in the gene POLG2 encoding the accessory subunit are frequently implicated in diseases associated with mitochondria-related diseases. A single mutation, G451E substitution in POLG2, is not involved in p55 dimerization, resulting in a late onset of adPEO (autosomal dominant progressive external ophthalmoplegia) and COX (cytochrome *c* oxidase) deficient muscle fibers [14].

In 2016, **Sayantan Datta *et al*.** adopted Illumina Golden Gate analysis to explore the association between oral precancerous lesions and cancer risk factors in cancer patients, patients with precancerous lesions (leukoplakia) and healthy controls, with the finding that Rs9905016 of POLG2 significantly elevated the risk of oral cancer. Compared with the wild type, the variant heterozygous (TC) significantly increased the transcription of POLG2. The cancer tissues with the variant allele (TC + CC) in POLG2 contained 1.6 times more mitochondrial DNA (***P*** < 0.01) than those with wild-type cancer tissues. It is speculated that the polymorphism of POLG2 increased the risk of oral cavity cancer, with the specific mechanism unknown whatsoever [21]. Based on the above descriptions, the POLG2 inherent role and potential therapeutic significance in the prognosis of prostate cancer are investigated by means of bioinformatic analysis and experimental verification.

## Materials and methods

### Gene expression and clinical characterization in TCGA

The Cancer Genome Atlas includes the uniform analysis of all 33 cancer types [22]. Data mining was conducted on the entire unbiased dataset with 11093 samples. The profile matrix and clinical information of POLG2 were applied in TCGA-PRAD incorporating 499 cancerous tissues and 52 normal para-cancerous tissues [23]. All gene expression profile matrix was standardized by R software (ver. 4.2.1). Stats package and car package are used for data analysis, while the ggplot2 package was employed for the analysis of differential gene expression and clinical characterization [24]. Kaplan-Meier curve was employed to depict survival curves by survivminer package, a survival package for visualization and statistical analysis. Base package and survival package were utilized for clinical characteristics and univariate multivariate analysis of the PRAD patients, respectively [23].

### GSE3325 and GSE8511

The GSE3325 and GSE8511 were downloaded from GEO database (Gene Expression Omnibus, https://www.ncbi.nlm.nih.gov/geo/). The gene expression profile of GSE3325 included 6 benign prostate tissues, 7 local primary PCa tissues, and 6 metastatic PCa tissues, which were available based on the GPL570 platform, while GSE8511 contained 16 benign prostate tissues, 12 local primary PCa tissues and 13 metastatic PCa tissues, which were available on different platform.

### Human protein atlas (HPA) and IHC

The images of histology staining for PCa and normal tissues were collected from HPA [25]. Tissue array of prostate cancer was purchased from Shanghai Outdo Biotech Company, of which 3 cases were normal, 49 cases were at different stages (I, II, III), and 8 cases were para-cancerous tissues. A total of 54 pathological tissues were involved in immunohistochemical analysis due to exfoliation or absence of glandular tissue. IHC was performed on formalin fixed paraffin-embedded tissue. Heat-based antigen retrieval was performed by boiling the tissue chip with pH6 citrate buffer (NobleRyder R8321, 1:100 dilution) in the autoclave. At the end of air exhaustion for 5 minutes, we opened the lid and allowed for natural cooling for over 30 minutes. POLG2 immunostaining was performed on the Launch i6000 IHC autostainer using a 1:80 dilution of primary antibody-binding POLG2 (Proteintech, 10997-2-AP) at 4°C overnight. DAB (Servicebio, G1212-200T) staining after dripping of biotin-labeled goat anti-rabbit antibody (Servicebio, G1213-100 μL), visualization of antibody binding using Aperio ScanScope XT. To avoid false negative results, we defined POLG2 expression of endothelial cells as a necessary internal control for samples. Two pathologists observed and scored the chip under the condition of double blind. The Allred scoring system was employed as the scoring standard, according to the intensity of cell staining and the proportion of positive cells. The final score was the sum of the score of positive staining intensity and that of positive staining cell proportion of each point. The cutoff of POLG2 positivity was defined as a score >5 (+++).

### Gene Ontology (GO) and KEGG Kyoto Encyclopedia of Genes and Genomes (KEGG) pathway enrichment analysis

As per the established screening criteria, the clinical information retained was discarded (owing to the loss of some clinical information of RNA seq data) and duplicate samples were removed. GO analysis has become a common gene annotation tool for large-scale genomic data [26]. KEGG is a path database, which collects a spectrum of databases of channel-related pathway correlations. DESeq2 package was utilized to analyze the differential genes from comparative RNA-seq data [27]. Cluster Profiler package was adopted for data conversion [28], while org.Hs.eg.db package (www.bioconductor.org/packages) was applied to ID conversion. Exclusion criteria for differential genes were |Log FC| > 1.5 and ***P***.adj < 0.05. Benjamini-Hochberg method was employed for P-value correction.

### Gene Set Enrichment Analysis (GSEA)

In this study, GSEA analysis involved the cluster profiler package in R, a cluster profiler for visualization and its gene set database from MSigDB Collection for expression-related pathways. The target sites |NES(Normalize Enrichment Score) | > 1.6, false discovery rate (FDR) < 25%, and *P*.adj < 0.05 were considered of significant enrichment [28].

### Correlation analysis and PPI network

With gene co-expression analysis, which depends upon correlations in gene expression levels, we analyzed the correlation with POLG2 gene expression with the use of the stat package in R. The top genes (|Spearman’s r| > 0.7) were involved in co-expressing heat map, with the ggplot2 package employed for visualization. PPI analysis aimed to explore the potential relationships was queried from STRING database, and the analysis of results was downloaded as per the application directions.

### Cell culture

Androgen-insensitive prostate cancer cell lines PC-3 and DU-145, androgen-sensitive cell lines LNCaP and 22RV1, and WPMY-1 cells were obtained from Jiangsu Institute of Tumor Biotherapy. Cells were maintained in petri dishes in a humidified atmosphere of 5% CO2 at 37°C with RPMI-1640 medium supplemented with 10% (vol/vol) FBS and 1% (vol/vol) penicillin–streptomycin and mycoplasma scavenger for temporary disinfection.

### RNA interference assay

Small interfering RNAs (siRNAs) targeting of POLG2 were constructed from Jiangsu GENEWIZ Biotech Co., Ltd. Transient transfection was performed with PolyFast Transfection Reagent (MCE, HY-K1014) and siRNA NC, S1, S2, S3 according to the manufacturer’s brochure. Culture medium was replaced 6h after transient transfection to ensure transfection efficiency and cell viability. The profiles of oligonucleotides targeting POLG2 were as follows: S1 Antisense UAAUACGUUCUCAAGAAAUGCTT, Sense GCAUUUCUUGAGAACGUAUUATT. S2 Antisense AUUAACAUAGUGUUCCAAGGC TT, Sense GCCUUGGAACACUAUGUUAAUTT. S3 Antisense UAAUUUAGACACAU UGCCAGGTT, Sense CCUGGCAAUGUGUCUAAAUUATT. NC Antisense ACGUGAC ACGUUCGGAGAATT, Sense UUCUCCGAACGUGUCACGUTT. At designated time points, the cells were collected for subsequent use. The knockdown efficiency of POLG2 was confirmed by qPCR at day 2 after transient transfection.

Lentivirus (LV)-non-targeting small hairpin RNA (shRNA) control, LV-POLG2 shRNA (TSGR20220311003) in pL-U6-shRNA-ccdB-puro vector were purchased from Tsingke Technologies with concentration 1×10^8^TU/ml. PC-3 and 22RV1 cells were infected with shRNA lentivirus, with their respective multiplicity of infection (MOI): PC-3(50 MOI), 22RV1(5 MOI) according to Lentivirus User Manual. Puromycin was added to eliminate non-transduced cells. Cells were collected after 5–7 days of infection and split for further experiments. Efficiency of shRNA was confirmed by RT-qPCR.

### RNA extraction, cDNA synthesis, and RT-qPCR

Cells were lysed in situ with the addition of TRIzol reagent (Invitrogen, 15596026). With total RNA purified according to the manufacturer’s instructions, cDNA was synthesized with HiFi Script gDNA Removal RT Master Mix reagent kit (CWBIO, Jiangsu, China) as per the manufacturer’s instructions. RNA quantification and purification were conducted on a Thermos NanoDrop 2000c Spectrophotometer. Quantitative real-time PCR was performed with TransStart Top Green qPCR superMix kit (TransGen biotech, AQ131-03) in a 20 μl volume that included cDNA, primers and qPCR superMix. The corresponding protocol followed the manufacturer’s instructions. β-actin was normalized to an endogenous control. The related primers were as follows: ACTB, F: TGACGTGGACATCCGCAAAG, ACTB, R: CTGGAAGGTGGACAG CGAGG. SnRNA U6, F: CTCGCTTCGGCAGCACA, SnRNA U6 R: AACGCTTCACGA ATTTGCGT. POLG2, F: GTCTA AATTACATGGCCGAGA, POLG2, R: TTTCCAAAT AACCAGGCCACA. E-cadherin, F: AGGCCAAGCAGCAGTA CATT, E-cadherin, R: ATTCACATCCAGCACATCCA. Vimentin, F: AGCTAACCAACG ACAAAGCC, Vimentin, R: TCCACTTTGCGTTCAAGGTC. α-SMA, F: CAATGTCCTA TCAGGGGGCAC, α-SMA, R: CGG CTTCATCGTATTCCTGTT. ZEB-1, F: GGCATAC ACCTACTCAACTACGG, ZEB-1, R: TGGGCGGTGTAGAATCAGAGTC. ZEB-2, F: CAAGAGGCGCAAACAAGCC, ZEB-2, R: GGTTGGCAATACCGTCATCC. Cycle threshold (Ct) values were analyzed with LC96 software (Light Cycle 96; Roche, USA) and calculated by means of 2^−ΔΔCt^ method.

### Mitochondrial dysfunction assay

Cell viability assay was conducted in cell lines DU-145 and 22RV1, which were seeded in six-well tissue culture plates at a density of 1.5 ×10^5^ cells/well. According to the above transient transfection protocol, cells in groups NC, S1, and S2 were seeded and cultured for 5 consecutive days in 96-well plates with a number of 2000 cells per well. Thereafter, at 0h and every 24 hours, cells were subjected to cell viability assay via CCK8 reagent (VICMED, Jiangsu, China), and optical density values were measured on CYTATION 3 imaging reader (BioTek Inc., Winooski, VT, USA) at a wavelength of 450 nm 2h after the addition of reagent, with data analyzed by GraphPad Prism 9.0.

ATP content detection was conducted in cell lines DU-145 and 22RV1. DU-145,22RV1 cell lines were treated with NC, S1, and S2 siRNA as previously described. Cells were collected by centrifugation after cell counting, and the supernatant was removed. The amount of ATP in cells was measured with the ATP content detection kit (BC0300, Solarbio, China). ATP extraction steps were performed in accordance with the manufacturer’s instructions. Approximately 8*10^5^ cells for each treatment group were measured in ATP content experiment. The ratio of ATP extraction solution(ml) to the cells was 1:5million. ATP content was detected using CYTATION 3 microplate reader. Optical density value A1 was measured at a wavelength of 340nm within approximately 10 seconds of the initiation of mixing and optical density value A2 was measured 3min after incubation in 37°C. ΔOD(A2-A1) was proportional to the ATP content, and ΔOD of each group was included in the statistical analysis.

### DNA synthesis test

The cell proliferation experiment was verified by Cell-Light EdU Apollo 567 In Vitro kit (RIBO Biological company, Guangdong, China). 48h after transient transfection, 22RV1and DU-145 cells were labeled by EdU nucleoside analog at a concentration of 50 μM for 2h, followed by paraformaldehyde fixation, Apollo staining and Hoechst 33342 DNA staining. Fluorescence microscopy was performed immediately at the same parameters, with the final statistics acquired with ImageJ and analyzed with GraphPad Prism 9.0.

### Wound healing assay

Both cell lines in the logarithmic growth phase were seeded in 6-well plates. At the confluence of 70%, each cell line was subjected to the transfection of NC, S1, and S2 siRNA as per transfection protocol. At 48h, the cells reached 90% confluence. Three parallel traces per well were aspirated with a pipette tip, and the cells were rinsed with PBS twice, followed by incubation with serum-free medium at 37°C. The scratch "healing" case was observed under a phase contrast microscope (Olympus, Japan) at 0h, 24h, and 48h, respectively. The scratching widths were measured and quantified with ImageJ software, with the experiment procedures conducted in triplicate.

To eliminate the compensatory effect, we conducted wound healing assay between PC-3 and 22RV1 stable cell lines, which infected with control lentivirus and POLG2 shRNA lentivirus. Experimental procedure and analytical method were the same as above.

### Western blotting

Si RNA mix (Equivalent S1 and S2) was applied to DU-145 and 22RV1 RNAi in order to eliminate potential off-target effects. The protein of NC group and its RNAi group were extracted 72h after transient transfection using the RIPA lysis buffer supplemented with protease inhibitor Cocktail (both reagents are from VICMED, Jiangsu, China). The corresponding protein concentrations were measured using BCA protein concentration determination reagent (Beyotime, Shanghai, China). GSEA analysis of POLG2 for expression-related pathways indicated that this gene is highly related to EMT (epithelial to mesenchymal transitions). The EMT-related protein of four groups (DU-145 NC, DU-145 Si, 22RV1 NC, and 22RV1 Si) were probed with the following rabbit-derived primary antibodies: anti-POLG2 (Proteintech,10997-2-AP,1:3000 dilution), anti-E-cad (Proteintech,20874-1-AP,1:4000 dilution), anti-N-cad (Proteintech,22018-1-AP,1:4000 dilution), anti-α-SMA (Servicebio, GB111364, 1:3000 dilution) and anti-actin (Servicebio, GB15001, 1:2000 dilution). Anti-rabbit secondary antibody (Servicebio, GB23303, 1:5000 dilution). Gel image system (Tanon, ver. 4.2) was used for visualization.

### Cell invasion assay

Experiments were carried out using Corning Matrigel Basement Membrane Matrix coated 8 mm PET transwell chambers (Corning). PC-3, 22RV1 cells were seeded in duplicates onto the coated transwell filters at a density of 0.8–1×10^5^ cells per well in medium containing 1% FBS, and RPMI-1640 containing 20% FBS was placed in the lower chamber as chemoattractant. Cells were allowed to invade for 14–24 h, non-invading cells were removed with a cotton swab and the invasive cells on the transwell insert were fixed in 4% polyformaldehyde for 20 min, and then stained with 0.1% crystal violet for 15min. Images were captured and analyzed by fluorescence microscopy. Five random fields at ×20 magnification were collected for each membrane. Each field was manually counted and analyzed with GraphPad Prism 9.0.

### Statistical analysis

In TCGA Database, the values of transcripts per million (TPM) and Student’s *t*-test were employed to calculate the significance of between-category gene expression divergence, with the expression profiles of POLG2 mRNA between normal and cancerous tissues analyzed by Wilcoxon rank sum test. The One-Way ANOVA was adopted to calculate the significance of gene expression in GSE3325, GSE8511, and the experimental difference between different treatment groups. Chi-square test, Fisher exact test, and Wilcoxon rank sum test were adopted to analyze the clinical characteristics of the PRAD and IHC staining. Univariate and Multivariate Cox analysis were employed for analysis of the prognostic values of POLG2 mRNA expression. The correlation of the targeted gene was evaluated via Spearman’s correlation coefficient. Log-rank test was implemented for comparison of survival curves, which were displayed as Kaplan–Meier plot. All statistical methods were performed with R package by default. Experimental difference of groups was performed by GraphPad Prism 9.0. All statistical tests were two-sided and *P* < 0.05 was considered significant.

### Ethics approval and consent to participate

There are no animal experiments and the tissue array for IHC staining was approved by the Ethics Committee of Shanghai Outdo Biotech Co., Ltd.

## Results

### Relationship between POLG2 expression and clinical characteristics

**Fig 1:**
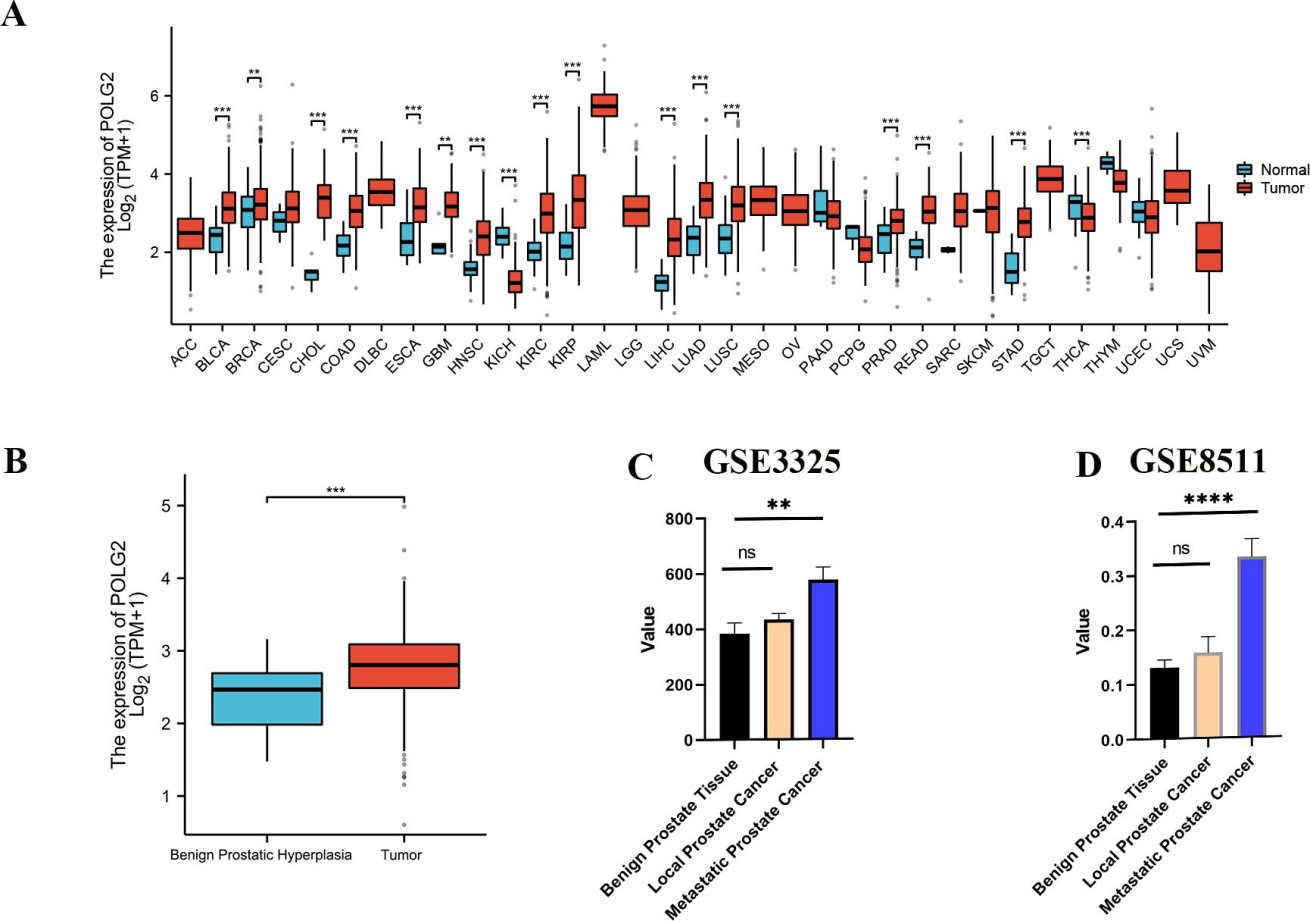
Relative expression levels of POLG2 in PRAD from TCGA and GEO database. **(A)** POLG2 expression levels in pan-cancers from TCGA database; **(B)** Box plot of POLG2 expression between benign prostatic hyperplasia tissues and tumor tissues in TCGA dataset (Normal = 52, Tumor = 499); (**C**, **D**) The POLG2 expression in GSE3325 and GSE8511 from GEO dataset (GSE3325 benign prostate tissue = 6, local tumor tissue = 7, metastatic tumor tissues = 6; GSE8511 benign prostate tissue = 16, local tumor tissue = 12, metastatic tumor tissue = 13). **, ***P*** < 0.01; ***, ***P*** < 0.001.

**Fig 2:**
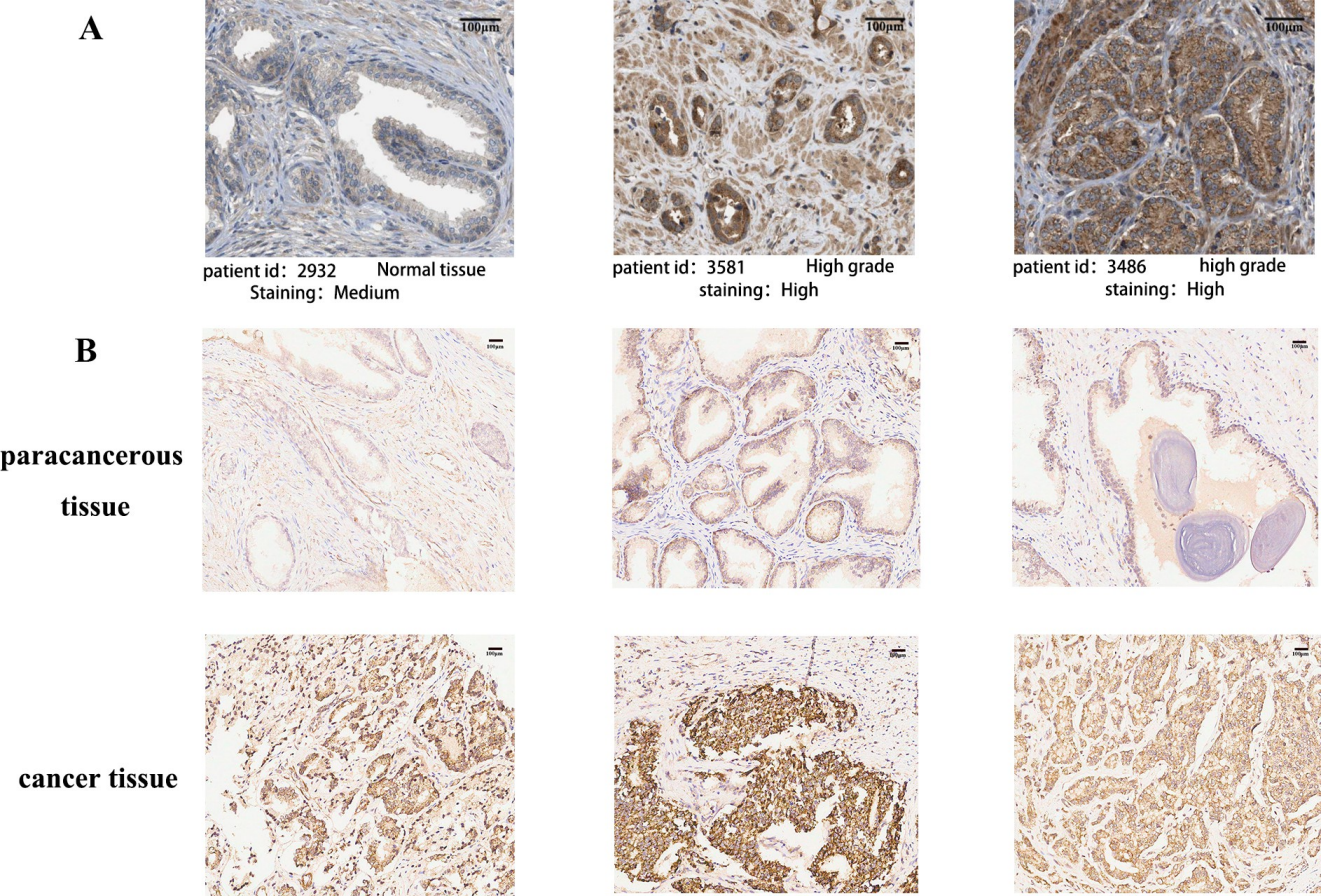
POLG2 immunohistochemical results from HPA database and tissue array. (**A**) POLG2 immunohistochemical results were consulted from HPA database (Normal tissue vs High grade tissue); (**B**)Tissue array for POLG2 immunohistochemical staining were displayed between para-cancerous tissues and cancerous tissues (different human pathologies). The bar was labeled in the upper right corner of each graph.

**Fig 3:**
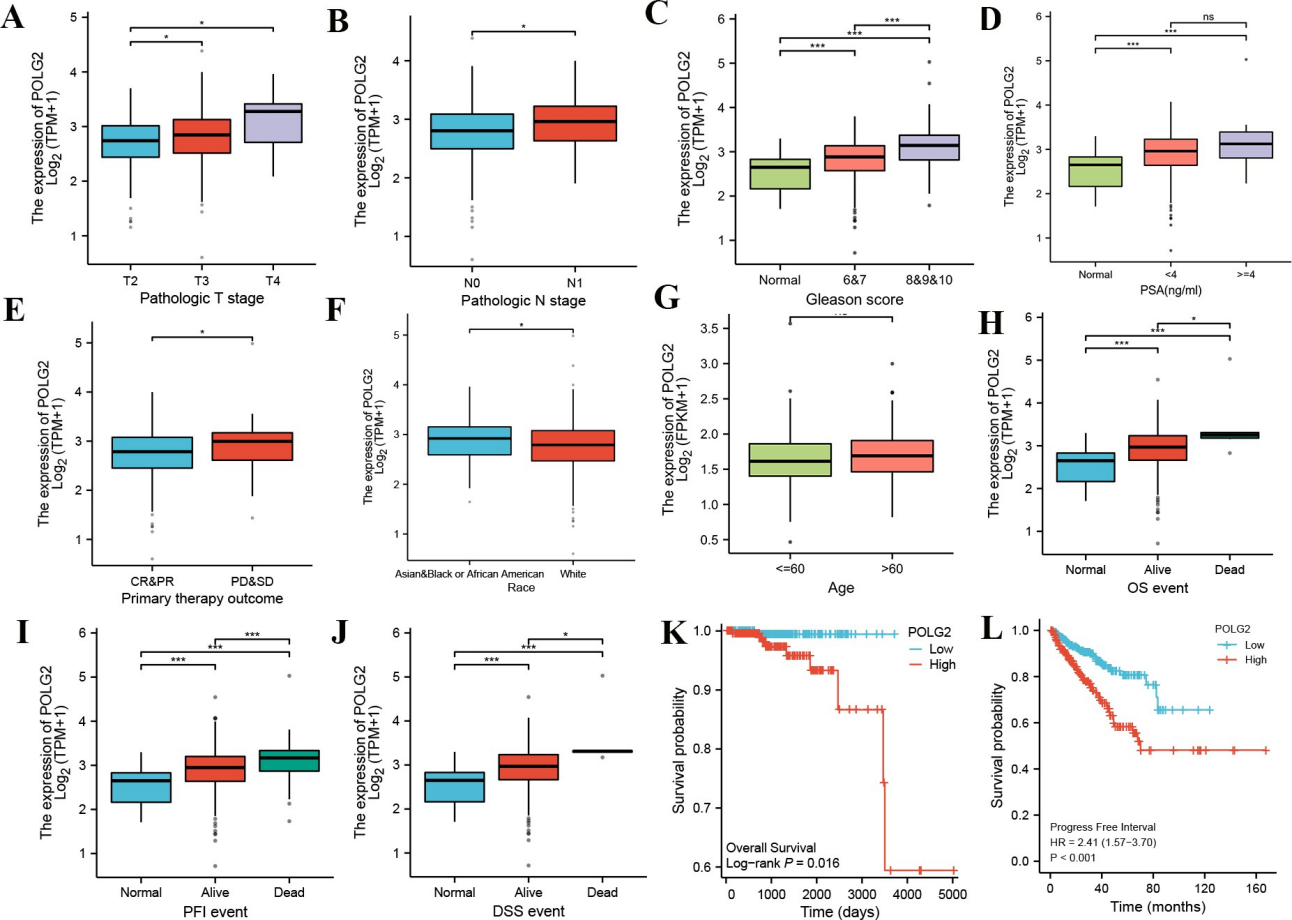
The expression of POLG2 was related to clinical characteristics in PRAD. **(A)** The expression of POLG2 in normal tissues and different pathologic T stages; (**B**) The expression of POLG2 in normal tissues and different pathologic N stages; (**C**) The expression levels of POLG2 in normal tissues and different Gleason scores; (**D**) The expression levels of POLG2 in normal tissues and different PSA levels; (**E**) The expression of POLG2 in normal tissues and different therapeutic outcomes; (**F**) The expression of POLG2 in different races; (**G**)The expression of POLG2 in different ages; (**H-J**) The expression levels of POLG2 among normal, alive, and dead in overall survival, progression-free interval, and disease-free survival; (**K, L**) K-M survival analysis of overall and progression-free interval in patients with low and high expression of POLG2. (ns: no significance; *, ***P*** < 0.05; ***, ***P*** < 0.001).

POLG2 was anomalously expressed in a wide range of tumors. The expression of POLG2 was analyzed in a variety of tumors using the data from TCGA. Significant differences were identified between quite a few cancer tissues and their normal counterparts, including BLCA (Bladder Cancer), BRCA (Breast Cancer), CHOL (Bile Duct Cancer), KIRC (Clear Cell Carcinoma), KIRP (Papillary Cell Carcinoma), READ (Rectal Cancer), STAD (Stomach Cancer), etc. (**Fig 1A**). POLG2 over-expression was observed in PRAD versus corresponding benign prostatic hyperplasia tissues (**Fig 1B**). The difference of POLG2 expression was also verified between GSE3325 and GES8511 at different stages of the disease. (**Fig 1C and 1D**). Meanwhile, the results of IHC in HPA and tissue array further confirmed that the expression of POLG2 was higher in cancerous tissue compared with normal adjacent tissues (**Fig 2A and 2B, Table 1, *p*** = 0.033) and POLG2 expression was related to malignancy of prostate cancer (**Table 2, *p*** = 0.038). Further clinical characterization in PRAD was performed, with the results that the over-expression of POLG2 was related to pathologic T, pathologic N stage, Gleason score, PSA values, primary therapy outcome and clinical survival (**Fig 3A–3E, 3H–3J**). Analysis results also indicated that demographic profile of patients such as ethnicity was also related to the expression of POLG2**(Fig 3F)**. Moreover, Kaplan-Meier survival curves revealed that over-expression of POLG2 actively had a significant impact on the survival probability of patients (**Fig 3K and 3L**). These results suggested that POLG2 expression was significantly correlated with clinical characteristics, but not with age (**Fig 3G**).

**Table 1:** Positive scores in para-cancerous tissues and prostate cancer tissues.

Prostate tissues	POLG2-positive score		Positivity rate (%)	*p*
	+—++	+++		
	Negative	Positive		
Para-cancerous tissues of prostate	6	2	25.0%	
Prostate cancer tissues	16	30	65.2%	**0.033**

POLG2 positivity is defined as a POLG2 IHC 3+.

**Table 2:** Clinical characteristics and immunohistochemical scores in tissue array.

Characteristic	N (46)	POLG2 positive score		*p*
		+—++	+++	
		Negative	Positive	
Age, n (%)				
≤ 65	15	7	8	
> 65	31	14	17	ns
Gleason score, n (%)				
6,7	30	19	11	
≥ 8	16	5	11	**0.038**
TNM				
Ⅰ, Ⅱ	35	20	15	
Ⅲ	11	4	7	ns

### GO/KEGG enrichment analysis and GSEA analysis

**Fig 4:**
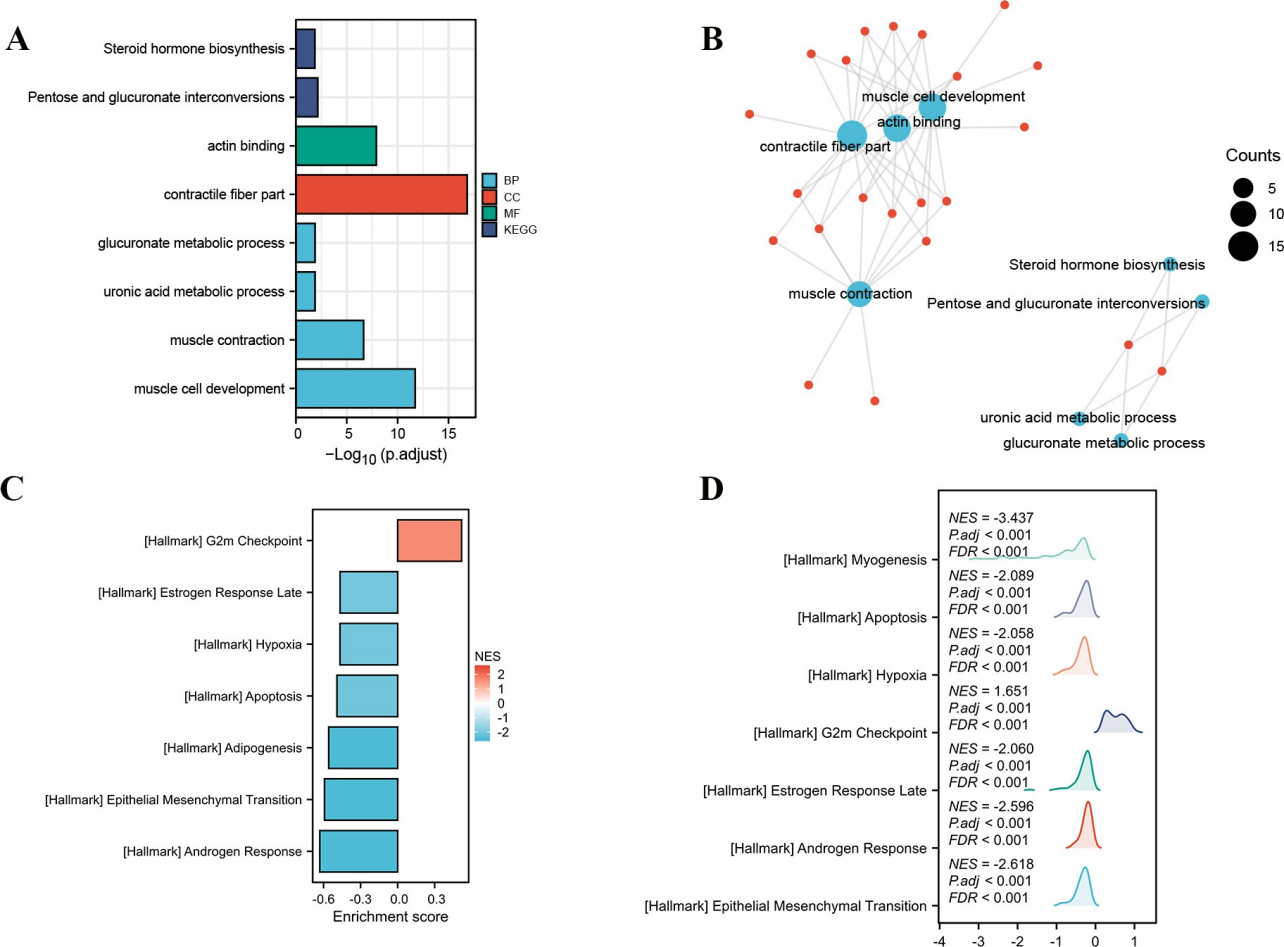
Functions and biological pathways of POLG2 were presented by GO/KEGG and GSEA. (**A, B**) GO/KEGG analysis was performed in histogram and was delineated in a visual network. The results revealed that POLG2 had functions of steroid hormone biosynthesis, muscle cell development and so on; (**C, D**) GSEA depicted biological pathways, and was visualized with a mountain map. The results indicated that POLG2 had biological pathways of epidermal mesenchymal transition, androgen response and so on.

The gene expression profiling analysis related to POLG2 was performed to further explore the biological function of POLG2 in PRAD. A total of 30 down-regulated genes and 16 up-regulated genes were considered significantly associated with POLG2 expression (|Log FC| > 1.5 and ***P***.adj < 0.05). GO/KEGG enrichment analysis was performed between the top 16 up-regulated genes and 30 down-regulated genes, the biological functions of these genes are associated mainly with pentose and glucuronate interconversions, steroid hormone biosynthesis, ascorbate and aldehyde acid metabolism, glucuronate metabolic process, etc. **(Fig 4A and 4B**).

GSEA analysis was conducted to identify functional and biological pathways in the genes for POLG2 differential analysis. As per the exclusion criteria described above, the enrichment signaling pathway with respect to POLG2 gene expression was selected. GSEA analysis results revealed that the over-expressed POLG2 phenotypes were concentrated mainly in (A) epithelial mesenchymal transition, (B) estrogen response late, (C) androgen response, (D) G2M checkpoint (**Fig 4C**), and the profiles was visualized with GSEA mountain map with ggplot2 package in R (**Fig 4D**).

### Relationship between POLG2 expression and PPI

**Fig 5:**
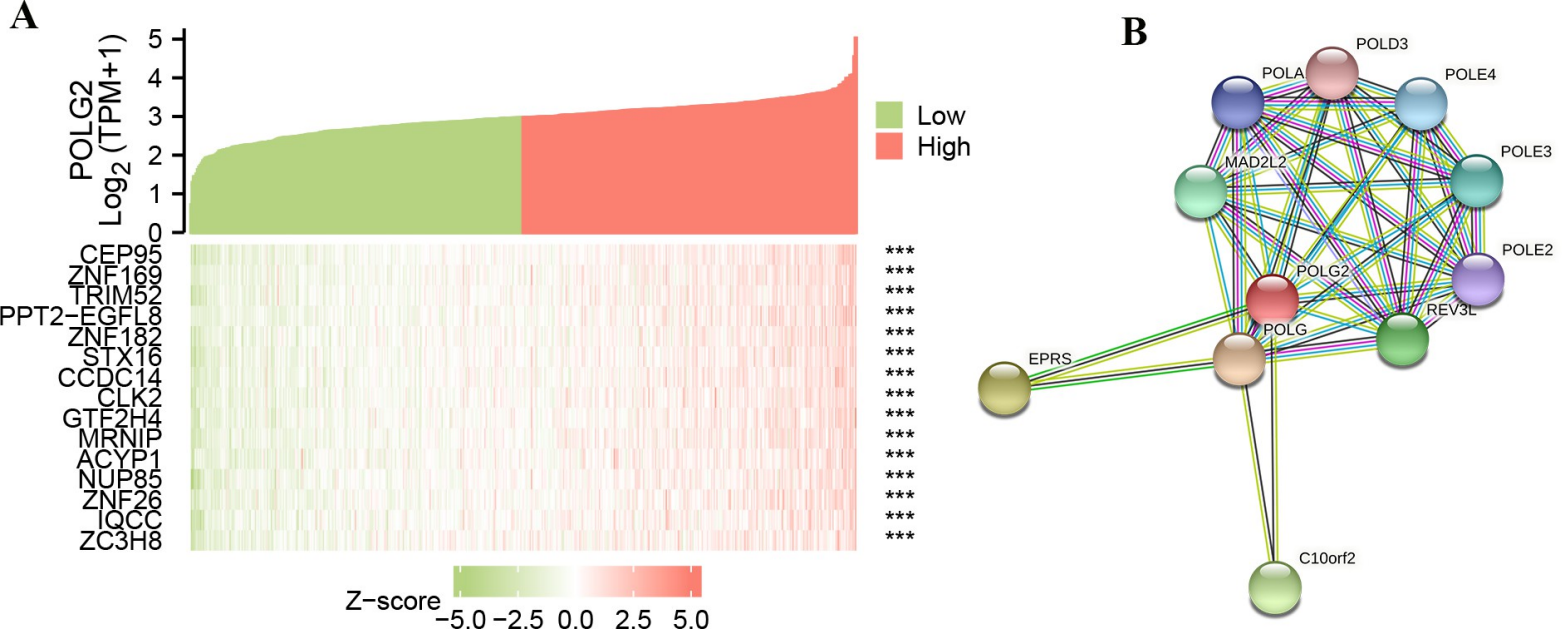
Genes related to POLG2 expression and PPI. (**A**) The top 15 genes were involved in the analysis of correlation with POLG2 gene expression; (**B**) The PPI network was displayed in STRING database. The functions of these genes were retrieved from NCBI (National Center for Biotechnology Information) database.

We performed a correlation analysis with POLG2 gene expression. The top 15 genes (|Spearman’s-r| > 0.7) were revealed in co-expression heat map (**Fig 5A**). The results indicated that these co-expression genes were related to cell cycle arrest, formation of spliceosomes, regulation of transcription, DNA binding activity, and tumor transformation and progression of several malignancies (Queried on NCBI website), which might be involved in its prognostic role in PRAD patients. The PPI network was analyzed based on STRING database and a small scale of genes with functions of DNA replication and mitotic spindle assembly checkpoint were identified (**Fig 5B**).

### Correlation between clinical characteristics and POLG2 expression in PRAD

We analyzed the correlations between clinical characteristics and POLG2 expression in PRAD. POLG2 mRNA expression was divided into high or low groups based on medium values. Survival analysis revealed that POLG2 over-expression was correlated with poor OS (***P*** = 0.016) and PFI (***P*** < 0.001). The analysis outcomes showed that high POLG2 mRNA expression was associated with T stage (***P*** = 0.013), OS event (***P*** = 0.02), Gleason scores (***P*** < 0.001), and races (***P*** = 0.028), respectively (**Table 3**). The univariate analysis revealed significant contributors to the overall survival of PRAD were M stage (***P*** < 0.001), Gleason scores (***P*** = 0.019), primary therapy (***P*** = 0.007), PSA values (***P*** = 0.001) and POLG2 mRNA expression (***P*** = 0.002), respectively. Multivariate analysis showed that POLG2 expression was an independent risk factor for overall survival in PRAD **(HR** = 7.23, ***P*** = 0.041, **Table 4**).

**Table 3:** The correlation between expression levels of POLG2 and clinical characteristics in PRAD.

Characteristic	Low expression of POLG2	High expression of POLG2	*p*
n	247	248	
T stage, n (%)			0.015
T2	108 (22.1%)	79 (16.2%)	
T3	133 (27.3%)	158 (32.4%)	
T4	3 (0.6%)	7 (1.4%)	
N stage, n (%)			0.119
N0	173 (41%)	171 (40.5%)	
N1	31 (7.3%)	47 (11.1%)	
M stage, n (%)			0.623
M0	225 (49.3%)	228 (50%)	
M1	2 (0.4%)	1 (0.2%)	
Age, n (%)			0.163
≤ 60	119 (24%)	103 (20.8%)	
> 60	128 (25.9%)	145 (29.3%)	
Race, n (%)			0.033
Asian	2 (0.4%)	10 (2.1%)	
Black or African American	24 (5%)	32 (6.7%)	
White	212 (44.2%)	200 (41.7%)	
PSA (ng/ml), n (%)			0.197
< 4	213 (48.6%)	198 (45.2%)	
≥ 4	10 (2.3%)	17 (3.9%)	
Gleason score, n (%)			< 0.0001
6,7	172 (34.75%)	119 (24.04%)	
8,9,10	75 (15.15%)	129 (26.06%)	
OS event, n (%)			0.020
Alive	246 (49.7%)	239 (48.3%)	
Dead	1 (0.2%)	9 (1.8%)	

**Table 4:** Clinical and pathological characteristics were performed using the cox proportional regression model.

Characteristics	Total (N)	Univariate analysis		Multivariate analysis	
		Hazard ratio (95% CI)	*P* value	Hazard ratio (95% CI)	*P* value
T stage	492				
T2	189	Reference			
T3&T4	303	3.294 (0.612–17.727)	0.165		
N stage	426				
N0	347	Reference			
N1	79	3.516 (0.778–15.896)	0.102		
M stage	458				
M0	455	Reference			
M1	3	59.383 (6.520–540.817)	**< 0.001**	9.292 (0.156–553.111)	0.285
Primary therapy outcome	438				
CR&SD	370	Reference			
PD&PR	68	8.683 (2.071–36.409)	**0.003**	5.004 (0.894–28.003)	0.067
Race	484				
White	415	Reference			
Black or African American & Asian	69	0.619 (0.118–3.244)	0.570		
PSA (ng/ml)	442				
<4	415	Reference			
≥ 4	27	10.479 (2.471–44.437)	**0.001**	1.552 (0.204–11.795)	0.671
Gleason score	499				
6&7	293	Reference			
8&9&10	206	6.664 (1.373–32.340)	**0.019**	1.828 (0.271–12.322)	0.535
POLG2	499	10.136 (2.279–45.079)	**0.002**	7.234 (1.087–48.140)	**0.041**

### Effect of POLG2 knockdown on prostate cancer cells with respect to proliferation, migration, and invasion

**Fig 6:**
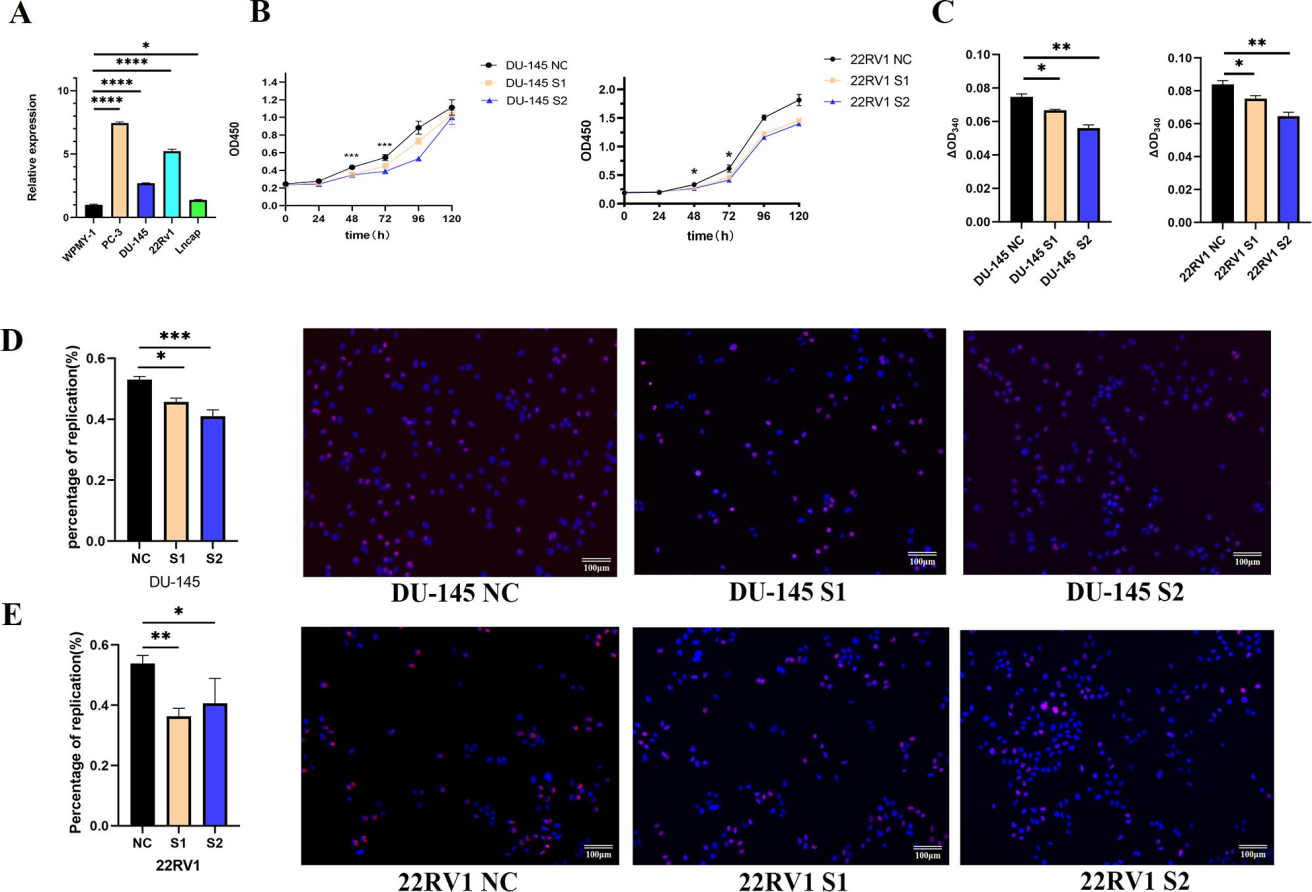
The effect of POLG2 knockdown on Mitochondrial function and proliferation in prostate cancer cells. (**A**) The expression of POLG2 in WPMY-1, PC-3, DU-145, 22RV1, and LNCaP, with the normal prostate cells (WPMY-1) were employed as a reference; (**B, C**) Cell viability test and ATP content detection were conducted between 22RV1 and DU-145 cell lines. Both cell lines were transfected with NC, S1, and S2 siRNA. In ATP content detection assay. Optical density value A1 was measured at a wavelength of 340nm within approximately 10 seconds of the initiation of mixing and optical density value A2 was measured 3min after incubation in 37°C at the same wavelength. ΔOD(A2-A1) of each group was included in the statistical analysis; (**D, E**) DNA synthesis test was validated in 22RV1, DU-145 cell lines. The number of red nuclei (proliferation cells) and blue nuclei (total cells) were quantified. The proportion of red nuclei /blue nuclei was included in the statistical analysis. (*, ***P*** < 0.05; ***, ***P*** < 0.001; ****, ***P*** < 0.0001. T-test was used for data analysis and plotted by GraphPad Prism 9.0).

The expression of POLG2 was verified in prostate cell lines. It was identified that the cell lines 22RV1 and PC-3 had relatively higher POLG2 expression than that in LNCaP cell line (**Fig 6A**). Cell viability assay was conducted every 24h from the beginning of POLG2 knockdown. S1, S2 siRNA were selected as the appropriate interference oligos due to the previous interference experiment based on qPCR verification. Knockdown effect showed significant difference compared with NC group after 2–4 days of transient transfection (**Fig 6B**). Next, we measured the ATP content experimentally between DU-145 and 22RV1 cell lines, a more intuitive representation of its knockdown effect. Results for S1, S2 groups were statistically significantly different than for the NC-treated group (**Fig 6C**). These results showed that POLG2 knockdown could interfere with mitochondrial function and impact ATP production. In order to estimate and validate mitochondrial dysfunction on cell proliferation, we conducted DNA synthesis test for verification. The results suggested evident proliferative inhibition of POLG2 knockdown in prostate cancer cells (**Fig 6D and 6E**).

**Fig 7:**
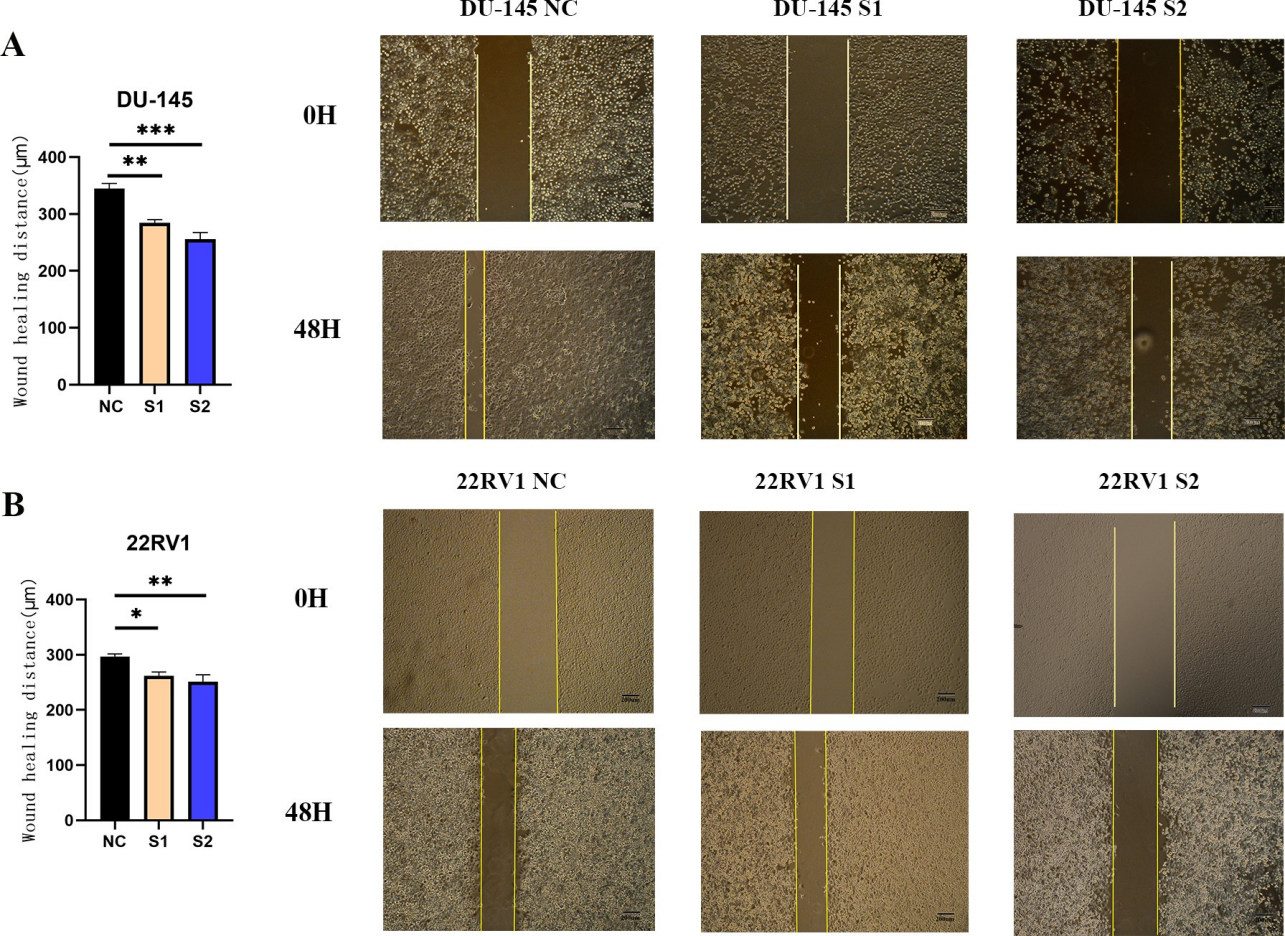
Cell migration assay was conducted in DU-145 and 22RV1. (**A**) The migration of DU-145 was displayed at 0h and 48h with the treatment of NC, S1and S2 siRNA. Wound-healing distances were analyzed within 48 hours; (**B**) The migration of 22RV1 was shown at 0h and 48h with treatment of NC, S1 and S2 siRNA. Wound-healing distances were analyzed within 48 hours. (**, ***P*** < 0.01; ***, ***P*** < 0.001. T-test was used for data analysis and plotted by GraphPad Prism 9.0).

The wound healing was a reaction of the migration ability of cancer cells. The 22RV1 and DU-145 cell lines underwent siRNA knockdown and treatment. The wound-healing distances were measured with the assistance of ImageJ software (**Fig 7A** and **7B**). The data presented a significant difference in migration between NC group and S1, S2 knockdown groups.

**Fig 8:**
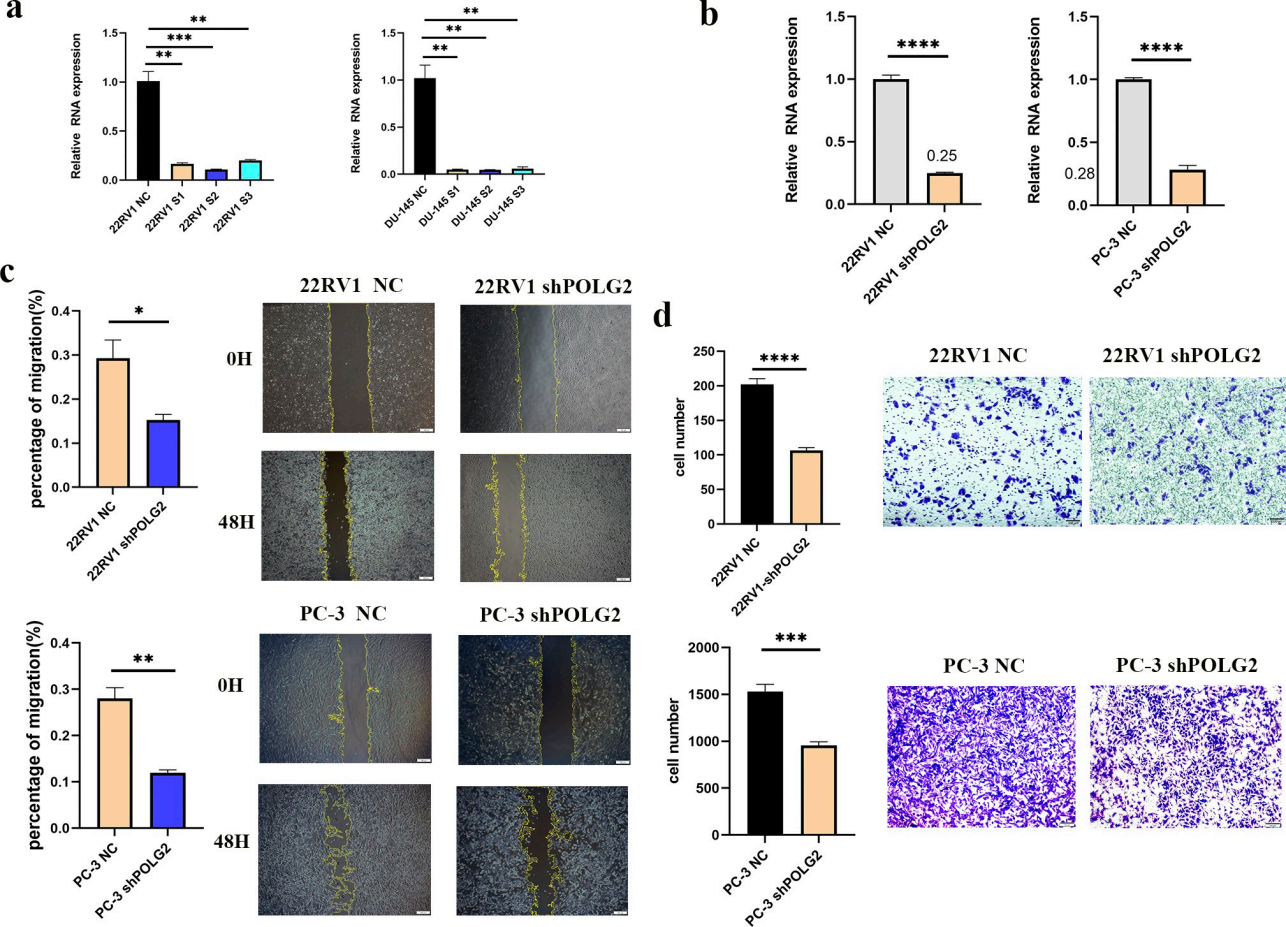
The migration and invasion assays in 22RV1 and PC-3 stable cell lines. (**A, B**) The efficiency of siRNAs and lentivirus shPOLG2 were assessed by RT-qPCR; (**C**) The migration of 22RV1 and PC-3 stable cell lines were shown at 0h and 48h. The percentage of wound closure was analyzed within 48 hours; (**D**) 22RV1 and PC-3 stable cell lines invasion experiments were conducted. The results of migration and invasion assays were analyzed using independent samples ***t***-test. (*, ***P*** < 0.05; **, ***P*** < 0.01; ***, ***P*** < 0.001. T-test was used for data analysis and plotted by GraphPad Prism 9.0).

The knockdown efficiency of POLG2 was validated in 22RV1 and DU-145 cell lines via S1, S2 and S3 siRNA (**Fig 8A**). Lentiviruses expressing shPOLG2 targeting S2 sense strand sequence were generated in 22RV1and PC-3. Knock-down efficiency was verified by qPCR (**Fig 8B**). To eliminate the impact of compensation effect on migration, PC-3, 22RV1 stable cell lines which had undergone lentivirus infection and selection were included in migration assay. The results show that POLG2 knockdown had a stable inhibitory effect on migration (**Fig 8C**). These data hinted that POLG2 knockdown had an inhibitory effect on mitochondrial function and motility in PCa cells. In order to understand whether POLG2 knockdown could affect the invasion of prostate cancer and exclude chronic compensatory response, we conducted cell invasion assay between PC-3, 22RV1 stable cell lines. The experimental results suggested that stable cell lines PC-3, 22RV1 showed obvious inhibition in invasion compared with NC group (**Fig 8D**).

### Effect of POLG2 knockdown on prostate cancer cells with respect to EMT

**Fig 9:**
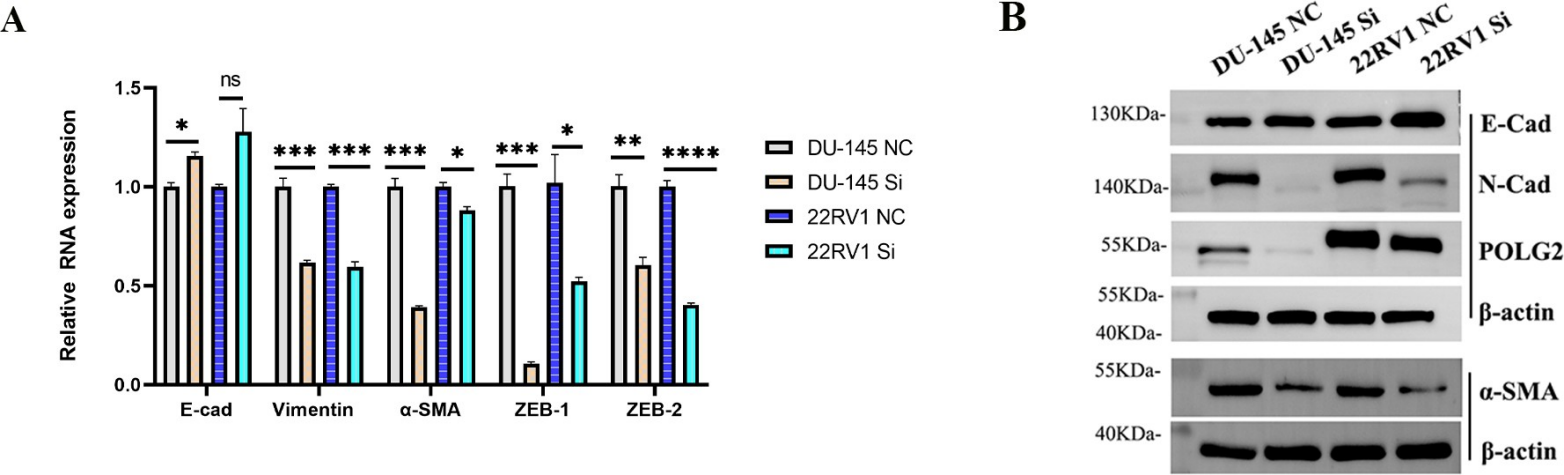
EMT-related genes and proteins expression were verified. (**A**) Effect of POLG2 knockdown by Si RNA mix (S1, S2) on the mRNA expression of EMT markers. E-cadherin, Vimentin, α-SMA, and upstream transcription factor ZEB-1, ZEB-2. Gene mRNA levels were normalized to U6 and expressed as fold changes with respect to the control group. Data were represented as mean ± SEM and the statistical analysis was performed using ANOVA. (ns: no significance; *, ***P****<* 0.05; **, ***P****<* 0.01; ***, ***P****<* 0.001; ****, ***P****<* 0.0001); (**B**) Western blotting for EMT markers in four groups (DU-145 NC, DU-145 Si, 22RV1 NC, and 22RV1 Si) was presented, α-SMA and β-actin below were complemented by experimental results based on the same protein sample. β-actin was used as a loading control.

Epithelial-mesenchymal transition (EMT) is an essential process and is often involved in tumor metastasis during development; Gene Set Enrichment Analysis (GSEA) revealed that high expression of POLG2 was related to EMT progression. To verify whether POLG2 knockdown could affect the expression of EMT-related genes, we conducted transient transfection in DU-145 and 22RV1 cell lines. The mRNA expression of EMT markers were assayed by RT-qPCR. We discovered that EMT marker genes such as E-cadherin, Vimentin, α-SMA caused expression variation (**Fig 9A**), and conventional Western blotting was applied to confirm the variation of protein expression (**Fig 9B**). Consistent with RT-qPCR and Western blotting assay, we observed that POLG2 knockdown elevated E-Cadherin but reduced N-Cadherin and α-SMA protein levels. Additionally, the upstream transcription factors ZEB-1, and ZEB-2 caused a substantial reduction in mRNA expression after POLG2 knockdown. These results were consistent with the results of GSEA’s EMT analysis.

## Discussion and conclusions

Currently, the treatments used for PCa cannot meet clinical needs [29]. AR signaling pathway reportedly plays a pivotal role in prostate cancer, both in hormone-sensitive prostate cancer or CRPC [29–31]. Meanwhile, AR gene amplification, locus mutation and AR splice variants (AR-Vs) have been implicated as mediators of resistance to AR-targeted therapy and progression of CRPC [32, 33]. Altered expression of AR coactivators and crosstalk between the AR and other signaling pathways render more complexity and difficulty to the treatment of prostate cancer [34].

However, due to the heterogeneity of prostate cell metabolism, the current studies mainly focus on the abnormalities of enzymes, pathways in aerobic glycolysis, and oncogenic signaling pathways [35], the progression of prostate cancer in mitochondrial metabolism has been virtually ignored.

In our study, we found that POLG2 was over-expressed in most cancer types, including in prostate cancer. Our results confirmed POLG2 expression was correlated with clinical stage, survival status, PSA, and Gleason scores, etc. In addition, the expression of POLG2 was correlated with the PCa progression and suggested poor OS and PFI. Multivariate analysis showed that POLG2 might be an independent prognostic factor of prostate cancer. We also performed GO/KEGG, GSEA analysis, co-expression genes, and PPI, and observed the genetic alterations related to POLG2 in PCa. In our in vitro experiments, we verified the expression of POLG2 in PCa cell lines, and the results suggested that the expression level of POLG2 was correlated with the degree of malignancy. POLG2 knockdown had an interference with mitochondrial function, impacted ATP production, and had potential inhibitory effect on proliferation, cell motility, and invasion; we also affirmed POLG2 could affect the prognosis of advanced prostate cancer via EMT. It is reported that any perturbation to key metabolic pathways in an organ is often strongly compensated by rewiring of metabolic fluxes in the same organ or systemically [36]. 22RV1 and PC-3 stable cell lines were involved in migration and invasion assays to eliminate interference caused by metabolic compensation. POLG2 has characteristics of enhancing mitochondrial DNA replication, affecting the material and energy metabolism and will ultimately have a great influence on cancer cells.

However, our research is not deep enough. Interference with mitochondrial metabolism mainly affects cell energy production. It is reported that AMP-activated protein kinase (AMPK) is an energy-sensing protein kinase which may regulate and control energy metabolism. AMPK is activated by various cellular stresses, for example, under energy-shortage condition, increased AMPK activity leads to a decrease in mTOR activity [37, 38]. Lower mTOR activity leads to inhibition of cell growth and protein synthesis. There is evidence that AMPK controls basic cellular functions by regulating microtubule dynamics through CLIP-170 phosphorylation [39], meanwhile, microtubule (MT) and actin filament networks cooperate functionally in directed cell and nuclear migration [40]. More and more cellular factors linking these cytoskeletal systems that had been identified in the past several years. These include myosin–CLIP170 complexes [41], dynein and the dynactin complex [42]. However, it should be noted that AMPK has also been reported to regulate multiple growth-related pathways, including Hedgehog signaling [43], the Hippo pathway [44, 45], the JAK–STAT signaling [46], and so on.

In summary, our study is the first work to explore the relationship between POLG2 expression and prostate cancer. We not only provide a molecular marker for advanced prostate cancer, but also the possibility of treating advanced prostate cancer via interfering with mitochondrial metabolism, which could serve as a therapeutic method for advanced prostate cancer.

## Supporting information

S1 File (ZIP)

## References

[1] Sung H, Ferlay J, Siegel RL, Laversanne M, Soerjomataram I, Jemal A, et al. Global Cancer Statistics 2020: GLOBOCAN Estimates of Incidence and Mortality Worldwide for 36 Cancers in 185 Countries. CA Cancer J Clin. 2021;71(3):209–49. Epub 2021/02/05. doi: 10.3322/caac.21660 . 33538338

[2] Massie CE, Lynch A, Ramos-Montoya A, Boren J, Stark R, Fazli L, et al. The androgen receptor fuels prostate cancer by regulating central metabolism and biosynthesis. EMBO J. 2011;30(13):2719–33. Epub 2011/05/24. doi: 10.1038/emboj.2011.158 ; PubMed Central PMCID: PMC3155295. 21602788 PMC3155295

[3] Siegel RL, Miller KD, Jemal A. Cancer statistics, 2020. CA Cancer J Clin. 2020;70(1):7–30. Epub 2020/01/09. doi: 10.3322/caac.21590 . 31912902

[4] Lu-Yao G, Nikita N, Keith SW, Nightingale G, Gandhi K, Hegarty SE, et al. Mortality and Hospitalization Risk Following Oral Androgen Signaling Inhibitors Among Men with Advanced Prostate Cancer by Pre-existing Cardiovascular Comorbidities. Eur Urol. 2020;77(2):158–66. Epub 2019/08/20. doi: 10.1016/j.eururo.2019.07.031 ; PubMed Central PMCID: PMC6980462. 31420248 PMC6980462

[5] Tran C, Ouk S, Clegg NJ, Chen Y, Watson PA, Arora V, et al. Development of a second-generation antiandrogen for treatment of advanced prostate cancer. Science. 2009;324(5928):787–90. Epub 2009/04/11. doi: 10.1126/science.1168175 ; PubMed Central PMCID: PMC2981508. 19359544 PMC2981508

[6] Fizazi K, Scher HI, Molina A, Logothetis CJ, Chi KN, Jones RJ, et al. Abiraterone acetate for treatment of metastatic castration-resistant prostate cancer: final overall survival analysis of the COU-AA-301 randomised, double-blind, placebo-controlled phase 3 study. The Lancet Oncology. 2012;13(10):983–92. doi: 10.1016/S1470-2045(12)70379-0 22995653

[7] Boysen G, Rodrigues DN, Rescigno P, Seed G, Dolling D, Riisnaes R, et al. SPOP-Mutated/CHD1-Deleted Lethal Prostate Cancer and Abiraterone Sensitivity. Clin Cancer Res. 2018;24(22):5585–93. Epub 2018/08/03. doi: 10.1158/1078-0432.CCR-18-0937 ; PubMed Central PMCID: PMC6830304. 30068710 PMC6830304

[8] Henning GM, Barashi NS, Smith ZL. Advances in Biomarkers for Detection, Surveillance, and Prognosis of Bladder Cancer. Clin Genitourin Cancer. 2021;19(3):194–8. Epub 2021/03/31. doi: 10.1016/j.clgc.2020.12.003 . 33781702

[9] Pavlova NN, Thompson CB. The Emerging Hallmarks of Cancer Metabolism. Cell Metab. 2016;23(1):27–47. Epub 2016/01/16. doi: 10.1016/j.cmet.2015.12.006 ; PubMed Central PMCID: PMC4715268. 26771115 PMC4715268

[10] DeBerardinis RJ, Chandel NS. Fundamentals of cancer metabolism. Sci Adv. 2016;2(5). ARTN e1600200 WOS:000380073000032. doi: 10.1126/sciadv.1600200 27386546 PMC4928883

[11] Costello LC, Franklin RB. The clinical relevance of the metabolism of prostate cancer; zinc and tumor suppression: connecting the dots. Mol Cancer. 2006;5:17. Epub 2006/05/17. doi: 10.1186/1476-4598-5-17 ; PubMed Central PMCID: PMC1481516. 16700911 PMC1481516

[12] Weinberg F, Hamanaka R, Wheaton WW, Weinberg S, Joseph J, Lopez M, et al. Mitochondrial metabolism and ROS generation are essential for Kras-mediated tumorigenicity. Proc Natl Acad Sci U S A. 2010;107(19):8788–93. Epub 2010/04/28. doi: 10.1073/pnas.1003428107 ; PubMed Central PMCID: PMC2889315. 20421486 PMC2889315

[13] Costello LC, Franklin RB. Novel role of zinc in the regulation of prostate citrate metabolism and its implications in prostate cancer. Prostate. 1998;35(4):285–96. Epub 1998/06/03. doi: 10.1002/(sici)1097-0045(19980601)35:4&lt;285::aid-pros8&gt;3.0.co;2-f . 9609552

[14] Longley MJ, Clark S, Yu Wai Man C, Hudson G, Durham SE, Taylor RW, et al. Mutant POLG2 disrupts DNA polymerase gamma subunits and causes progressive external ophthalmoplegia. Am J Hum Genet. 2006;78(6):1026–34. Epub 2006/05/11. doi: 10.1086/504303 ; PubMed Central PMCID: PMC1474082. 16685652 PMC1474082

[15] Anderson S, Bankier AT, Barrell BG, de Bruijn MH, Coulson AR, Drouin J, et al. Sequence and organization of the human mitochondrial genome. Nature. 1981;290(5806):457–65. Epub 1981/04/09. doi: 10.1038/290457a0 . 7219534

[16] Johnson AA, Tsai Y, Graves SW, Johnson KA. Human mitochondrial DNA polymerase holoenzyme: reconstitution and characterization. Biochemistry. 2000;39(7):1702–8. Epub 2000/02/26. doi: 10.1021/bi992104w . 10677218

[17] Carrodeguas JA, Bogenhagen DF. Protein sequences conserved in prokaryotic aminoacyl-tRNA synthetases are important for the activity of the processivity factor of human mitochondrial DNA polymerase. Nucleic Acids Res. 2000;28(5):1237–44. Epub 2000/02/10. doi: 10.1093/nar/28.5.1237 ; PubMed Central PMCID: PMC102604. 10666468 PMC102604

[18] Lim SE, Longley MJ, Copeland WC. The mitochondrial p55 accessory subunit of human DNA polymerase gamma enhances DNA binding, promotes processive DNA synthesis, and confers N-ethylmaleimide resistance. J Biol Chem. 1999;274(53):38197–203. Epub 1999/12/23. doi: 10.1074/jbc.274.53.38197 . 10608893

[19] Farge G, Pham XH, Holmlund T, Khorostov I, Falkenberg M. The accessory subunit B of DNA polymerase gamma is required for mitochondrial replisome function. Nucleic Acids Res. 2007;35(3):902–11. Epub 2007/01/26. doi: 10.1093/nar/gkl1116 ; PubMed Central PMCID: PMC1807957. 17251196 PMC1807957

[20] Copeland WC. Inherited mitochondrial diseases of DNA replication. Annu Rev Med. 2008;59:131–46. Epub 2007/09/26. doi: 10.1146/annurev.med.59.053006.104646 ; PubMed Central PMCID: PMC2271032. 17892433 PMC2271032

[21] Datta S, Ray A, Roy R, Roy B. Association of DNA sequence variation in mitochondrial DNA polymerase with mitochondrial DNA synthesis and risk of oral cancer. Gene. 2016;575(2 Pt 3):650–4. Epub 2015/09/26. doi: 10.1016/j.gene.2015.09.039 . 26403317

[22] Bailey MH, Tokheim C, Porta-Pardo E, Sengupta S, Bertrand D, Weerasinghe A, et al. Comprehensive Characterization of Cancer Driver Genes and Mutations. Cell. 2018;173(2):371–85 e18. Epub 2018/04/07. doi: 10.1016/j.cell.2018.02.060 ; PubMed Central PMCID: PMC6029450. 29625053 PMC6029450

[23] Liu J, Lichtenberg T, Hoadley KA, Poisson LM, Lazar AJ, Cherniack AD, et al. An Integrated TCGA Pan-Cancer Clinical Data Resource to Drive High-Quality Survival Outcome Analytics. Cell. 2018;173(2):400–16 e11. Epub 2018/04/07. doi: 10.1016/j.cell.2018.02.052 ; PubMed Central PMCID: PMC6066282. 29625055 PMC6066282

[24] Wickham H. (2009). ggplot2: Elegant Graphics for Data Analysis. Use R!Springer Science & Business Media, Heidelberg, Berlin, Germany

[25] Uhlen M, Fagerberg L, Hallstrom BM, Lindskog C, Oksvold P, Mardinoglu A, et al. Proteomics. Tissue-based map of the human proteome. Science. 2015;347(6220):1260419. Epub 2015/01/24. doi: 10.1126/science.1260419 . 25613900

[26] Hulsegge I, Kommadath A, Smits MA. Globaltest and GOEAST: two different approaches for Gene Ontology analysis. BMC Proc. 2009;3 Suppl 4:S10. Epub 2009/07/21. doi: 10.1186/1753-6561-3-S4-S10 ; PubMed Central PMCID: PMC2712740. 19615110 PMC2712740

[27] Love MI, Huber W, Anders S. Moderated estimation of fold change and dispersion for RNA-seq data with DESeq2. Genome Biol. 2014;15(12):550. Epub 2014/12/18. doi: 10.1186/s13059-014-0550-8 ; PubMed Central PMCID: PMC4302049. 25516281 PMC4302049

[28] Yu G, Wang LG, Han Y, He QY. clusterProfiler: an R package for comparing biological themes among gene clusters. OMICS. 2012;16(5):284–7. Epub 2012/03/30. doi: 10.1089/omi.2011.0118 ; PubMed Central PMCID: PMC3339379. 22455463 PMC3339379

[29] Hu C, Xu H, Li Z, Liu D, Zhang S, Fang F, et al. Juglone promotes antitumor activity against prostate cancer via suppressing glycolysis and oxidative phosphorylation. Phytother Res. 2022. Epub 2022/10/26. doi: 10.1002/ptr.7631 . 36281060

[30] van der Kwast TH, Schalken J, Ruizeveld de Winter JA, van Vroonhoven CC, Mulder E, Boersma W, et al. Androgen receptors in endocrine-therapy-resistant human prostate cancer. Int J Cancer. 1991;48(2):189–93. Epub 1991/05/10. doi: 10.1002/ijc.2910480206 . 1708363

[31] Visakorpi T, Hyytinen E, Koivisto P, Tanner M, Keinanen R, Palmberg C, et al. In vivo amplification of the androgen receptor gene and progression of human prostate cancer. Nat Genet. 1995;9(4):401–6. Epub 1995/04/01. doi: 10.1038/ng0495-401 . 7795646

[32] Lev A, Lulla AR, Ross BC, Ralff MD, Makhov PB, Dicker DT, et al. ONC201 Targets AR and AR-V7 Signaling, Reduces PSA, and Synergizes with Everolimus in Prostate Cancer. Mol Cancer Res. 2018;16(5):754–66. Epub 2018/03/29. doi: 10.1158/1541-7786.MCR-17-0614 ; PubMed Central PMCID: PMC5932216. 29588330 PMC5932216

[33] Swinnen JV, Heemers H, van de Sande T, de Schrijver E, Brusselmans K, Heyns W, et al. Androgens, lipogenesis and prostate cancer. J Steroid Biochem Mol Biol. 2004;92(4):273–9. Epub 2005/01/25. doi: 10.1016/j.jsbmb.2004.10.013 . 15663990

[34] Berger A, Rickman DS. The Role of Androgen Receptor in Prostate Cancer. Precision Molecular Pathology of Prostate Cancer. Molecular Pathology Library 2018. p. 345–65.

[35] Lin C, Salzillo TC, Bader DA, Wilkenfeld SR, Awad D, Pulliam TL, et al. Prostate Cancer Energetics and Biosynthesis. Adv Exp Med Biol. 2019;1210:185–237. Epub 2020/01/05. doi: 10.1007/978-3-030-32656-2_10 ; PubMed Central PMCID: PMC8096614. 31900911 PMC8096614

[36] Yang L, Li P, Yang W, Ruan X, Kiesewetter K, Zhu J, et al. Integrative Transcriptome Analyses of Metabolic Responses in Mice Define Pivotal LncRNA Metabolic Regulators. Cell Metab. 2016;24(4):627–39. Epub 2016/09/27. doi: 10.1016/j.cmet.2016.08.019 ; PubMed Central PMCID: PMC5181118. 27667668 PMC5181118

[37] Inoki K, Zhu T, Guan KL. TSC2 mediates cellular energy response to control cell growth and survival. Cell. 2003;115(5):577–90. Epub 2003/12/04. doi: 10.1016/s0092-8674(03)00929-2 . 14651849

[38] Gwinn DM, Shackelford DB, Egan DF, Mihaylova MM, Mery A, Vasquez DS, et al. AMPK phosphorylation of raptor mediates a metabolic checkpoint. Mol Cell. 2008;30(2):214–26. Epub 2008/04/29. doi: 10.1016/j.molcel.2008.03.003 ; PubMed Central PMCID: PMC2674027. 18439900 PMC2674027

[39] Nakano A, Kato H, Watanabe T, Min KD, Yamazaki S, Asano Y, et al. AMPK controls the speed of microtubule polymerization and directional cell migration through CLIP-170 phosphorylation. Nat Cell Biol. 2010;12(6):583–90. Epub 2010/05/25. doi: 10.1038/ncb2060 . 20495555

[40] Goode BL, Drubin DG, Barnes G. Functional cooperation between the microtubule and actin cytoskeletons. Current Opinion in Cell Biology. 2000;12(1):63–71. doi: 10.1016/s0955-0674(99)00058-7 WOS:000085475200008. 10679357

[41] Benashski SE, Harrison A, Patel-King RS, King SM. Dimerization of the highly conserved light chain shared by dynein and myosin V. J Biol Chem. 1997;272(33):20929–35. Epub 1997/08/15. doi: 10.1074/jbc.272.33.20929 . 9252421

[42] Karki S, Holzbaur EL. Cytoplasmic dynein and dynactin in cell division and intracellular transport. Curr Opin Cell Biol. 1999;11(1):45–53. Epub 1999/02/27. doi: 10.1016/s0955-0674(99)80006-4 . 10047518

[43] Li YH, Luo J, Mosley YY, Hedrick VE, Paul LN, Chang J, et al. AMP-Activated Protein Kinase Directly Phosphorylates and Destabilizes Hedgehog Pathway Transcription Factor GLI1 in Medulloblastoma. Cell Rep. 2015;12(4):599–609. Epub 2015/07/21. doi: 10.1016/j.celrep.2015.06.054 ; PubMed Central PMCID: PMC4521589. 26190112 PMC4521589

[44] Mo JS, Meng Z, Kim YC, Park HW, Hansen CG, Kim S, et al. Cellular energy stress induces AMPK-mediated regulation of YAP and the Hippo pathway. Nat Cell Biol. 2015;17(4):500–10. Epub 2015/03/10. doi: 10.1038/ncb3111 ; PubMed Central PMCID: PMC4380774. 25751140 PMC4380774

[45] Wang W, Xiao ZD, Li X, Aziz KE, Gan B, Johnson RL, et al. AMPK modulates Hippo pathway activity to regulate energy homeostasis. Nat Cell Biol. 2015;17(4):490–9. Epub 2015/03/10. doi: 10.1038/ncb3113 ; PubMed Central PMCID: PMC4380807. 25751139 PMC4380807

[46] Rutherford C, Speirs C, Williams JJ, Ewart MA, Mancini SJ, Hawley SA, et al. Phosphorylation of Janus kinase 1 (JAK1) by AMP-activated protein kinase (AMPK) links energy sensing to anti-inflammatory signaling. Sci Signal. 2016;9(453):ra109. Epub 2016/12/06. doi: 10.1126/scisignal.aaf8566 . 27919027

