# Supplementary material for: Interference with mitochondrial metabolism could serve as a potential therapeutic strategy for advanced prostate cancer
Source: PLoS One. 2024 Apr 10;19(4):e0290753. doi: 10.1371/journal.pone.0290753 (PMC11006138; doi:10.1371/journal.pone.0290753)
Supplement: S1 File — (ZIP) [file pone.0290753.s001.zip › CofA for 80-727472163.pdf]

GENEWIZ LLC
115 Corporate Boulevard
South Plainfield, NJ 07080
p (877) GENEWIZ (436-3949)
f (908) 333-4511
www.genewiz.com

GWCN/III-03-DR01-R1

Certificate of Analysis

Customer	Quote #	GENEWIZ Clone ID #
吴闯	80-727472163	RN270

Gene Name	Gene ID

Purification	Date

Name	Sequence (5'→3')	mer	total nmols	nmol/ tube	MW (Dalton)	Tm (℃)	GC (%)	OD/ tube	add x ul to 100 uM	Modification
hPOLG2 si-1 sense	GCAUUUCUUGAGAACGUAUUATT	23	5.00	5.00	7267.4	53.00	30.40	1.17	50.00	
hPOLG2 si-1 antisense	UAAUACGUUCUCAAGAAAUGCTT	23	5.00	5.00	7273.5	53.00	30.40	1.17	50.00	
hPOLG2 si-2 sense	GCCUUGGAACACUAUGUUAAUTT	23	5.00	5.00	7266.4	54.80	34.80	1.14	50.00	
hPOLG2 si-2 antisense	AUUAACAUAGUGUUCCAAGGCTT	23	5.00	5.00	7289.5	54.80	34.80	1.17	50.00	
hPOLG2 si-3 sense	CCUGGCAAUGUGUCUAAAUUATT	23	5.00	5.00	7266.4	54.80	34.80	1.14	50.00	
hPOLG2 si-3 antisense	UAAUUUAGACACAUUGCCAGGTT	23	5.00	5.00	7289.5	54.80	34.80	1.17	50.00	
d-NC sense			2.50	2.50			0		25	
d-NC antisense			2.50	2.50			0		25	
d-NC-FAM sense			2.50	2.50			0		25	5`6-FAM
d-NC-FAM antisense			2.50	2.50			0		25	5`6-FAM
hACTB sense			2.50	2.50			0		25	
hACTB antisense			2.50	2.50			0		25	
