# Supplementary material for: Interference with mitochondrial metabolism could serve as a potential therapeutic strategy for advanced prostate cancer
Source: PLoS One. 2024 Apr 10;19(4):e0290753. doi: 10.1371/journal.pone.0290753 (PMC11006138; doi:10.1371/journal.pone.0290753)
Supplement: S1 File — (ZIP) [file pone.0290753.s001.zip › grey value analysis.docx]

| 灰度值(integrated density val) | | | | | |
| --- | --- | --- | --- | --- | --- |
| 编号 | E-Cad | N-Cad | Vimentin | POLG2 | ACTIN |
| Du-145 | 382314 | 361344 | 429358 | 275625 | 764315 |
| Du-145 Si | 495805 | 82450 | 150959 | 83069 | 751066 |
| 22RV1 | 548330 | 487698 | 233587 | 805211 | 754563 |
| 22RV1 Si | 755522 | 173999 | 231849 | 608972 | 750320 |

| 指标灰度值/内参灰度值（ratio） | | | | | |
| --- | --- | --- | --- | --- | --- |
|  | E-CAD | N-CAD | Vimentin | POLG2 |  |
| Du-145 | 0.50 | 0.47 | 0.56 | 0.36 |  |
| Du-145 Si | 0.66 | 0.11 | 0.20 | 0.11 |  |
| 22RV1 | 0.73 | 0.65 | 0.31 | 1.06 |  |
| 22RV1 Si | 1.00 | 0.23 | 0.31 | 0.81 |  |

| 灰度值(integrated density val) | | |
| --- | --- | --- |
| 编号 | α-SMA | ACTIN |
| Du-145 | 307518 | 473442 |
| Du-145 Si | 197778 | 476011 |
| 22RV1 | 279539 | 483554 |
| 22RV1 Si | 146979 | 489336 |

| 指标灰度值/内参灰度值（ratio） | |
| --- | --- |
|  | α-SMA |
| Du-145 | 0.650 |
| Du-145 Si | 0.415 |
| 22RV1 | 0.578 |
| 22RV1 Si | 0.301 |
