# Supplementary material for: Interference with mitochondrial metabolism could serve as a potential therapeutic strategy for advanced prostate cancer
Source: PLoS One. 2024 Apr 10;19(4):e0290753. doi: 10.1371/journal.pone.0290753 (PMC11006138; doi:10.1371/journal.pone.0290753)

GENEWIZ LLC
115 Corporate Boulevard
South Plainfield, NJ 07080
p (877) GENEWIZ (436-3949)
f (908) 333-4511
www.genewiz.com



# Certificate of Analysis

**Customer**
吴闯

**Quote #**
80-727472163

**Name**
hPOLG2 si-3 antisense

**Sequence（5'→3'）**
UAAUUUAGACACAUUGCCAGGTT

| | |
|---|---|
| **Length:** | 23 |
| **nmoles:** | 5.00 |
| **TM(℃)：** | 54.80 |
| **MV(target)：** | 7289.452 |
| **GC(%)** | 34.80% |
| **Purification:** | HPLC |
| **Modification(5'→3'):** | |
| **add water to 100 uM** | 50.00 |

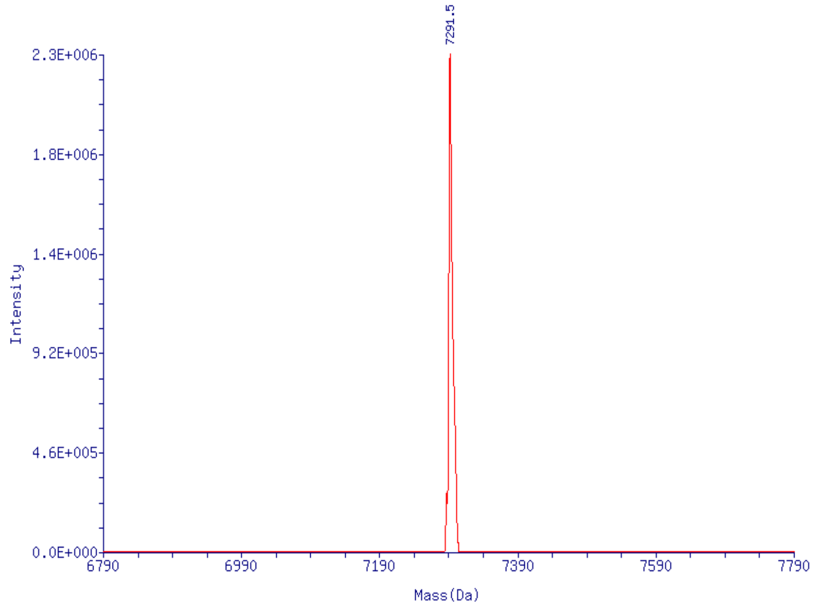

Supplement: S1 File — (ZIP) [file pone.0290753.s001.zip › renamed_66f35.pdf]
