# Supplementary material for: Interference with mitochondrial metabolism could serve as a potential therapeutic strategy for advanced prostate cancer
Source: PLoS One. 2024 Apr 10;19(4):e0290753. doi: 10.1371/journal.pone.0290753 (PMC11006138; doi:10.1371/journal.pone.0290753)
Supplement: S1 File — (ZIP) [file pone.0290753.s001.zip › renamed_ab5a8.pdf]

苏州金唯智生物科技有限公司
中国江苏省苏州市工业园区星湖街 218 生物纳米园 C3 栋
电话：0512-68731001 传真：0512-62629530
genewiz.com.cn

化学合成 siRNA oligo 使用说明

一、 金唯智 siRNA 产品

产品类型	纯化方式	规格
普通 siRNA	HPLC	5nmol/10nmol
修饰 siRNA	HPLC	5nmol/10nmol
阴性对照（siRNA N-CTL）	HPLC	2.5nmol
siRNA 人、大鼠、小鼠阳性对照	HPLC	2.5nmol
转染对照（siRNA FAM-N-CTL）	HPLC	2.5nmol
siRNA 三保一套餐<br>（3 个靶点、阴性对照、阳性对照、转染对照）	HPLC	5nmol/10nmol

二、 siRNA 产品运输与保存

- 产品经低温真空抽干以后以粉状态在常温下运输，收到产品后请置于-20℃~-80℃保存，干粉可以稳定保存一年，短期内若无实验计划，建议干粉保存。
- RNA 以膜或干粉状态附着于管壁，开盖之前务必请瞬时离心，使用前加 RNase Free Water 溶解成 20μM 的储存液，分装后置于-20℃~-80℃保存，以利于后续实验，且避免反复冻融（最多 5 次），溶解后 siRNA 在-80℃可保存 6 个月。
- siRNA 容易降解，所有操作需严格遵循 RNA 操作规则，实验结束后剩余储存液请及时保存于-20℃~-80℃。

三、 使用前说明

化学合成 siRNA 一般为 19nt + 3’-overhangs 的互补双链，其中 19nt 是由 RNA 碱基组成的 siRNA 靶序列，此区域为互补双链，3’-overhangs 是悬垂于正反义链 3’-端的两个碱基，一般是由 DNA 碱基组成（也可以是 RNA 碱基），化学合成时分别合成正反义链，然后等摩尔量混合经 PCR 仪退火形成双链。

- 正义链（Sense），或称 Passenger Strand，与靶基因上设计的靶点序列相同（U 取代 T），3’-端有两个悬垂 DNA 碱基；反义链（Antisense），或称 Guide Strand，序列与靶点序列完全互补（U 取代 T），3’-端有两个悬垂 DNA 碱基；

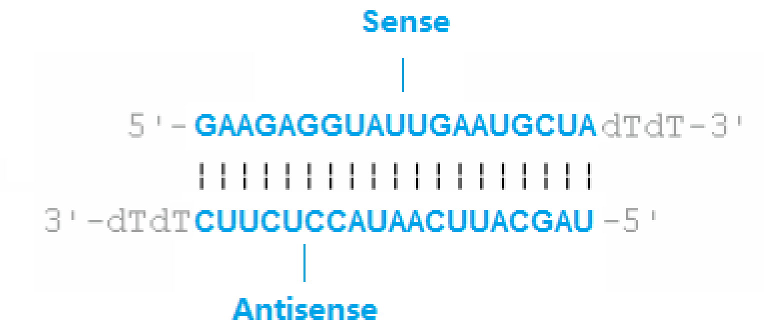

- 本公司所有 siRNA 合成之后均经过 RP-HPLC 纯化，纯度在 90%以上，且经过质谱质检，分子量正确；
- 本公司提供的 siRNA 均以摩尔为单位，一是便于退火时可以等量混合，二是转染时方便计算。且 nmol、OD 和μg 之间可以互相转换，一般来说，一对 21bp 的 siRNA 平均分子量约为 13300，不同单位之间转换有如下关系：

1OD 双链≈2.5nmol≈33μg

- siRNA 产品溶解为 20μM 储存液浓度所需的加水量，可简单参考下表：

siRNA（nmol）	2.5（1OD）	5（2OD）	10（4OD）
溶解体积（μl）	125	250	500

四、 细胞使用说明

siRNA 是通过转染的方式进入细胞，适用于磷酸钙共沉淀、电穿孔法、DEAE-葡聚糖和 polybrene、机械法和阳离子脂质体试剂（如 Lipofectamine2000）等多种转染方法，严格按照各方法的操作步骤即可。一般根据细胞来选择具体的转染试剂和转染方法，对于容易转染的细胞，阳离子脂质体是较为常用的转染方法。

为了保证实验的可重复性与可靠性，实验过程中需严格控制细胞密度、试剂用量、转染效率等因素对实验结果的影响，一般建议实验中每组至少 3 重复，同一批次实验所有孔内细胞密度保持一致且在孔内均匀分布，转染后检测转染效率，最好不要低于 70%。

五、 转染浓度选择与优化

在接种细胞之前，确保细胞状态良好。贴壁细胞转染密度推荐在 50%左右；悬浮细胞推荐常规培养细胞数的 1/3 进行转染。转染时培养基中不能加抗生素，会降低转染效率和导致细胞死亡；为了获得更好的结果，可以使用 Invitrogen 的 Opti-Medium 低血清培养基在形成复合物前稀释 Lipofectamin 2000 和 siRNA。可以使用荧光标记的 siRNA 帮助优化细胞系的转染条件。一旦确定转染的最佳条件，可在每一次实验都包括荧光标记 siRNA，作为转染效率的指示剂。

由于细胞类型及实验目的的不同，siRNA 最佳的工作浓度也会有所变化，为获得有效的目的基因 Knockdown 效果，初次实验强烈建议设置不同的浓度梯度进行优化，优化范围一般在 10-100nM。若不做梯度摸索，初次实验转染浓度一般推荐用 50nM，但不能保证是最佳浓度，通常基因沉默分析至少在转染后的 24-72h 检测。

为了达到高的转染效率，在实验过程中，需要注意以下几点：1）实验要在无 RNase 的环境下进行；2）细胞状态保持良好，建议使用对数生长期的细胞进行实验；3）避免使用抗生素；3）选择合适的转染试剂；4）通过荧光标记的转染对照分析 siRNA 的稳定性及转染效率进而优化实验。

六、 实验对照设计

对于一个严谨的实验来说，除了敲低靶基因的实验组之外，还需要设计多组实验对照以确定实验不同环节的可靠性，常用的有以下几种对照组：

- 阴性对照组：用非特异性的 siRNA 序列证明 RNA 干扰作用的序列特异性，一般是与目的细胞无同源性的通用阴性对照 siRNA 序列，也可以是目的 siRNA 序列打乱（scramble）的普通阴性对照，阴性对照是实验必需的；
- 转染试剂对照组：转染过程中不加任何 siRNA，用来排除转染试剂对细胞毒性或细胞成活率的影响；
- 空白对照组：转染过程中不加任何试剂，单独用来监测实验过程中细胞的生长状态；
- 阳性对照组：阳性对照作为一个实验系统检查很重要，可以用来确认 siRNA 干扰实验的转染、RNA 提取和基因表达检测方法是可靠的；

- 转染对照组：用 FAM 荧光标记的通用阴性对照，用于优化转染条件和监测转染效率，FAM 标记与 GFP 的荧光波长范围相差不大，可以直接以检测 GFP 的通道观察，需要注意的是 FAM 标记在转染之后荧光会逐渐淬灭，因此最佳的观察时间建议在转染之后的 8h 左右。

七、 siRNA 干扰效果检测

根据细胞种类、转染方法和检测手段的不同，最佳检测时间会有所差异，一般在转染后 24-72h 进行检测。

- qRT-PCR 检测靶基因 mRNA 水平，检测 siRNA 干扰效果最直接的方法，siRNA 进入细胞后在 RISC 系统下直接作用于靶细胞的 mRNA，导致其被降解，因此通过 qRT-PCR 检测靶基因的 mRNA 水平可直接反映 siRNA 是否起作用，一般在转染后的 24-48 小时检测，与对照相比可以看到靶基因 mRNA 水平明显下调。注：qRT-PCR 引物的设计和质量很重要。
- Western Blot 检测靶基因蛋白表达水平，对于蛋白编码功能基因来说，利用 siRNA 干扰的目的就是降低基因的蛋白表达水平从而研究该基因的功能，因此需要同时检测靶基因的蛋白水平。但由于蛋白表达水平受到多个过程调控，因此会有靶基因 mRNA 水平下调而蛋白水平无明显变化的情况出现。
- 细胞功能学实验检测，对于已知功能的靶基因来说，还可以通过细胞功能学实验来检测 siRNA 的干扰效果，常规的比如细胞的增殖、凋亡、侵袭和迁移。

八、 转染步骤举例

由于阳离子脂质体试剂的 Lipofectamine2000 目前使用广泛，且效果良好，故在此以 Lipofectamine 的转染为例。下表为使用 Invitrogen 的 Lipofectamine 2000 转染时的转染试剂及 siRNA 用量，siRNA 初始储存液浓度为 20μM，仅供参考：

苏州金唯智生物科技有限公司
中国江苏省苏州市工业园区星湖街 218 生物纳米园 C3 栋
电话：0512-68731001 传真：0512-62629530
genewiz.com.cn

培养板	每孔总体积（V1+V2+V2)	终浓度	siRNA/孔	Lipo2000/孔
96 孔板	100μl (50μl+25μl+25μl)	10nM	0.05μl	0.3μl
	100μl (50μl+25μl+25μl)	20nM	0.1μl	0.3μl
	100μl (50μl+25μl+25μl)	50nM	0.25μl	0.3μl
	100μl (50μl+25μl+25μl)	100nM	0.5μl	0.3μl
24 孔板	500μl (400μl+50μl+50μl)	10nM	0.25μl	1.2μl
	500μl (400μl+50μl+50μl)	20nM	0.5μl	1.2μl
	500μl (400μl+50μl+50μl)	50nM	1.25μl	1.2μl
	500μl (400μl+50μl+50μl)	100nM	2.5μl	1.2μl
12 孔板	1mL (800μl+100μl+100μl)	10nM	0.5μl	2.4μl
	1mL (800μl+100μl+100μl)	20nM	1μl	2.4μl
	1mL (800μl+100μl+100μl)	50nM	2.5μl	2.4μl
	1mL (800μl+100μl+100μl)	100nM	5μl	2.4μl
6 孔板	2mL (1500μl+250μl+250μl)	10nM	1μl	6μl
	2mL (1500μl+250μl+250μl)	20nM	2μl	6μl
	2mL (1500μl+250μl+250μl)	50nM	5μl	6μl
	2mL (1500μl+250μl+250μl)	100nM	10μl	6μl

其中 V1 为完全或不完全培养基，铺细胞时使用，V2 为 Opti-Medium，转染专用，无血清、无抗生素，一部分加转染试剂，一部分加 siRNA。 转染步骤（以 24 孔板为例）：

准备细胞：

- 贴壁细胞：转染前一天，在 400μl 无抗培养基中接种 0.5-2×10^5 个细胞，转染时细胞融合度为 50%。(注：铺板时要将细胞消化完全混匀，避免细胞堆积生长)
- 悬浮细胞：转染前一天，在 400μl 无抗培养基中接种 0.5-2×10^5 个细胞，转染时细胞数量应在 4-8×10^5/孔。

转染过程：

- 用 50μl Opti-Medium 稀释 siRNA (转染细胞的终浓度为 50nM)，轻轻吹吸 3-5 次混匀。
- 轻轻颠倒混匀转染试剂，用 50μl Opti-Medium 稀释 1.2μl Lipofectamine 2000，轻轻吹打 3-5 次混匀，室温下静置 5min。

苏州金唯智生物科技有限公司
中国江苏省苏州市工业园区星湖街 218 生物纳米园 C3 栋
电话：0512-68731001 传真：0512-62629530
genewiz.com.cn

- 混合转染试剂和 siRNA 稀释液，轻轻吹吸 3-5 次混匀，室温下静置 20min。
- 转染复合物加入到 24 孔细胞板中，100μl/孔，前后轻摇细胞板混合均匀。
- 细胞培养板置于 37℃、5% CO_2 培养箱中培养 24-48h。转染 4-6h 后可换新鲜培养基。
