# Supplementary material for: Interference with mitochondrial metabolism could serve as a potential therapeutic strategy for advanced prostate cancer
Source: PLoS One. 2024 Apr 10;19(4):e0290753. doi: 10.1371/journal.pone.0290753 (PMC11006138; doi:10.1371/journal.pone.0290753)

GENEWIZ LLC
115 Corporate Boulevard
South Plainfield, NJ 07080
p (877) GENEWIZ (436-3949)
f (908) 333-4511
www.genewiz.com

GWCN/III-03-DR01-R1

Certificate of Analysis

Customer	Quote #
吴闯	80-727472163

Name	Sequence（5'→3'）
hPOLG2 si-3 sense	CCUGGCAAUGUGUCUAAAUUATT

**Length:**	23	**Purification:**	HPLC
**nmoles:**	5.00	**Modification(5'→3'):**	
**TM(℃)：**	54.80	**add water to 100 uM**	50.00
**MV(target)：**	7266.412		
**GC(%)**	34.80%		

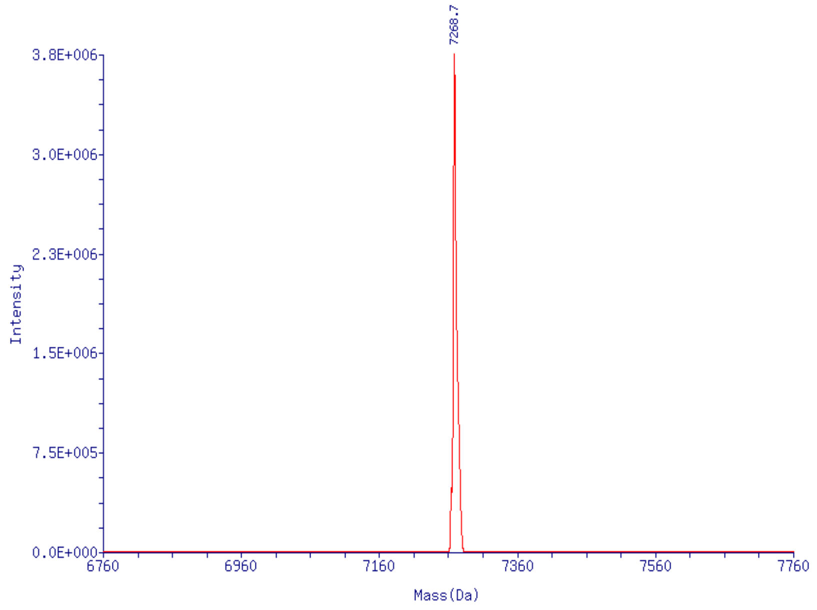

Supplement: S1 File — (ZIP) [file pone.0290753.s001.zip › renamed_ac487.pdf]
