# Supplementary material for: Interference with mitochondrial metabolism could serve as a potential therapeutic strategy for advanced prostate cancer
Source: PLoS One. 2024 Apr 10;19(4):e0290753. doi: 10.1371/journal.pone.0290753 (PMC11006138; doi:10.1371/journal.pone.0290753)

GENEWIZ LLC
115 Corporate Boulevard
South Plainfield, NJ 07080
p (877) GENEWIZ (436-3949)
f (908) 333-4511
www.genewiz.com

GWCN/III-03-DR01-R1

Certificate of Analysis

**Customer**	**Quote #**
吴闯	80-727472163
**Name**	**Sequence（5'→3'）**
hPOLG2 si-1 sense	GCAUUUCUUGAGAACGUAUUATT

**Length:**	23	**Purification:**	HPLC
**nmoles:**	5.00	**Modification(5'→3'):**	
**TM(℃)：**	53.00	**add water to 100 uM**	50.00
**MV(target)：**	7267.397		
**GC(%)**	30.40%		

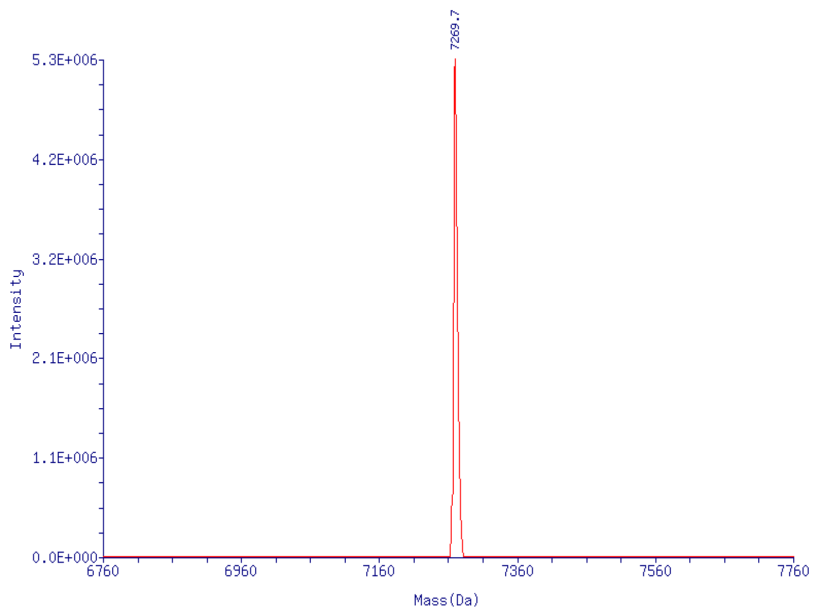

Supplement: S1 File — (ZIP) [file pone.0290753.s001.zip › renamed_ea32b.pdf]
