# Supplementary material for: Interference with mitochondrial metabolism could serve as a potential therapeutic strategy for advanced prostate cancer
Source: PLoS One. 2024 Apr 10;19(4):e0290753. doi: 10.1371/journal.pone.0290753 (PMC11006138; doi:10.1371/journal.pone.0290753)
Supplement: S1 File — (ZIP) [file pone.0290753.s001.zip › renamed_f4c5f.pdf]

GENEWIZ LLC
115 Corporate Boulevard
South Plainfield, NJ 07080
p (877) GENEWIZ (436-3949)
f (908) 333-4511
www.genewiz.com

GWCN/III-03-DR01-R1

# Certificate of Analysis

| Customer | Quote # |
|---|---|
| 吴闯 | 80-727472163 |

| Name | Sequence（5'→3'） |
|---|---|
| hPOLG2 si-2 sense | GCCUUGGAACACUAUGUUAAUTT |

| | | | |
|---|---|---|---|
| **Length:** | 23 | **Purification:** | HPLC |
| **nmoles:** | 5.00 | **Modification(5'→3'):** | |
| **TM(℃)：** | 54.80 | **add water to 100 uM** | 50.00 |
| **MV(target)：** | 7266.412 | | |
| **GC(%)** | 34.80% | | |

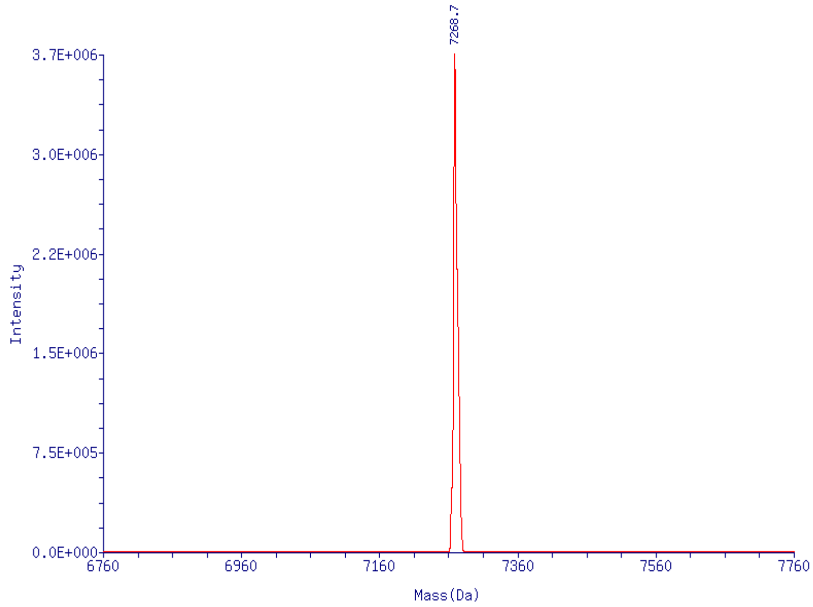


South Plainfield, NJ • Boston, MA • Washington, DC Metro • Research Triangle Park, NC • San Diego, CA • Los Angeles, CA • San Francisco, CA • Seattle, WA • London, UK • Langen, DE • Beiging, China • Suzhou, China • Tokyo, Japan
