# Supplementary figures and images for: Interference with mitochondrial metabolism could serve as a potential therapeutic strategy for advanced prostate cancer
Source: PLoS One. 2024 Apr 10;19(4):e0290753. doi: 10.1371/journal.pone.0290753 (PMC11006138; doi:10.1371/journal.pone.0290753)

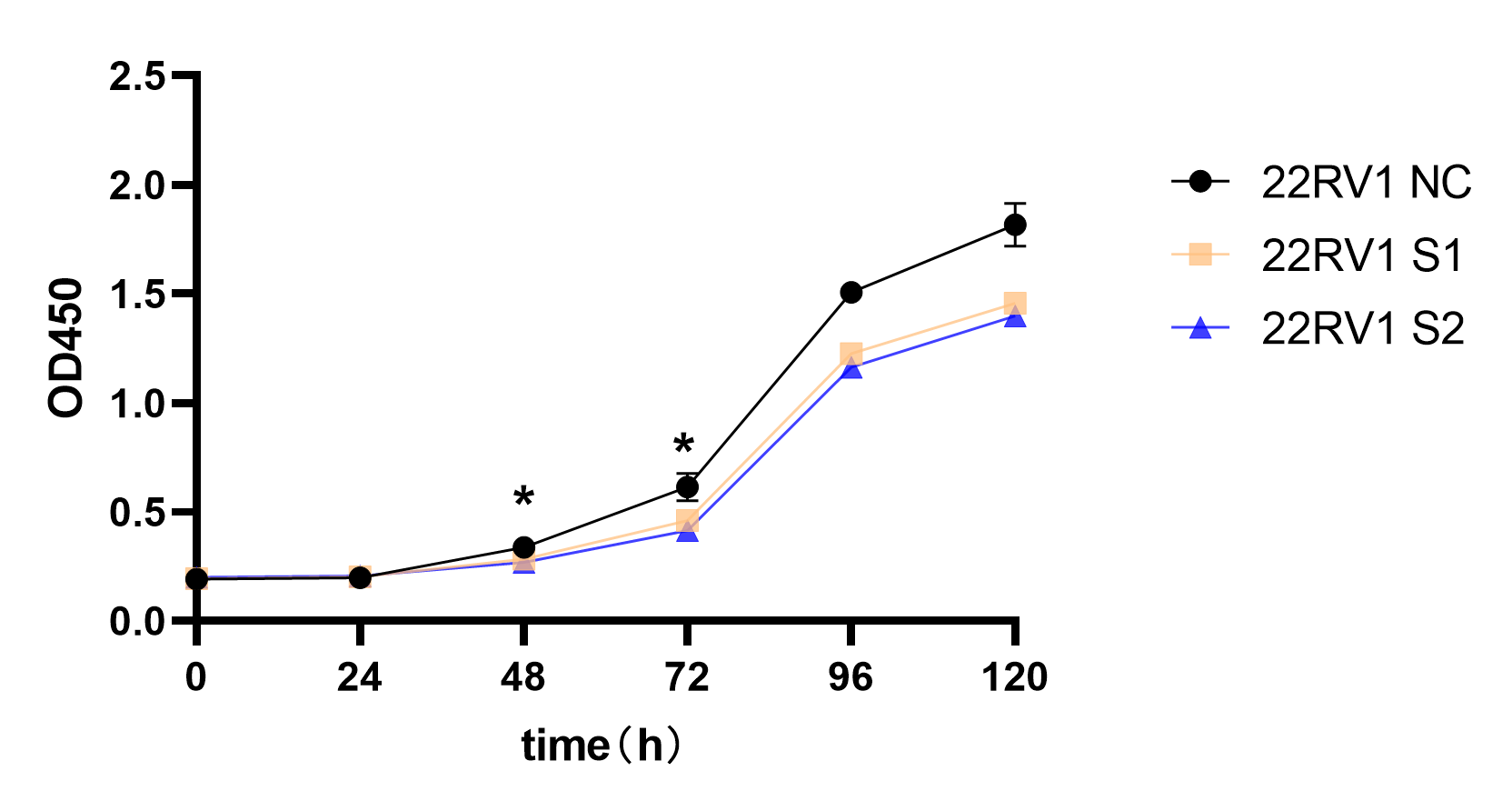

Supplement: S1 File — (ZIP) [file pone.0290753.s001.zip › 22rv1 CCK-8.tif]

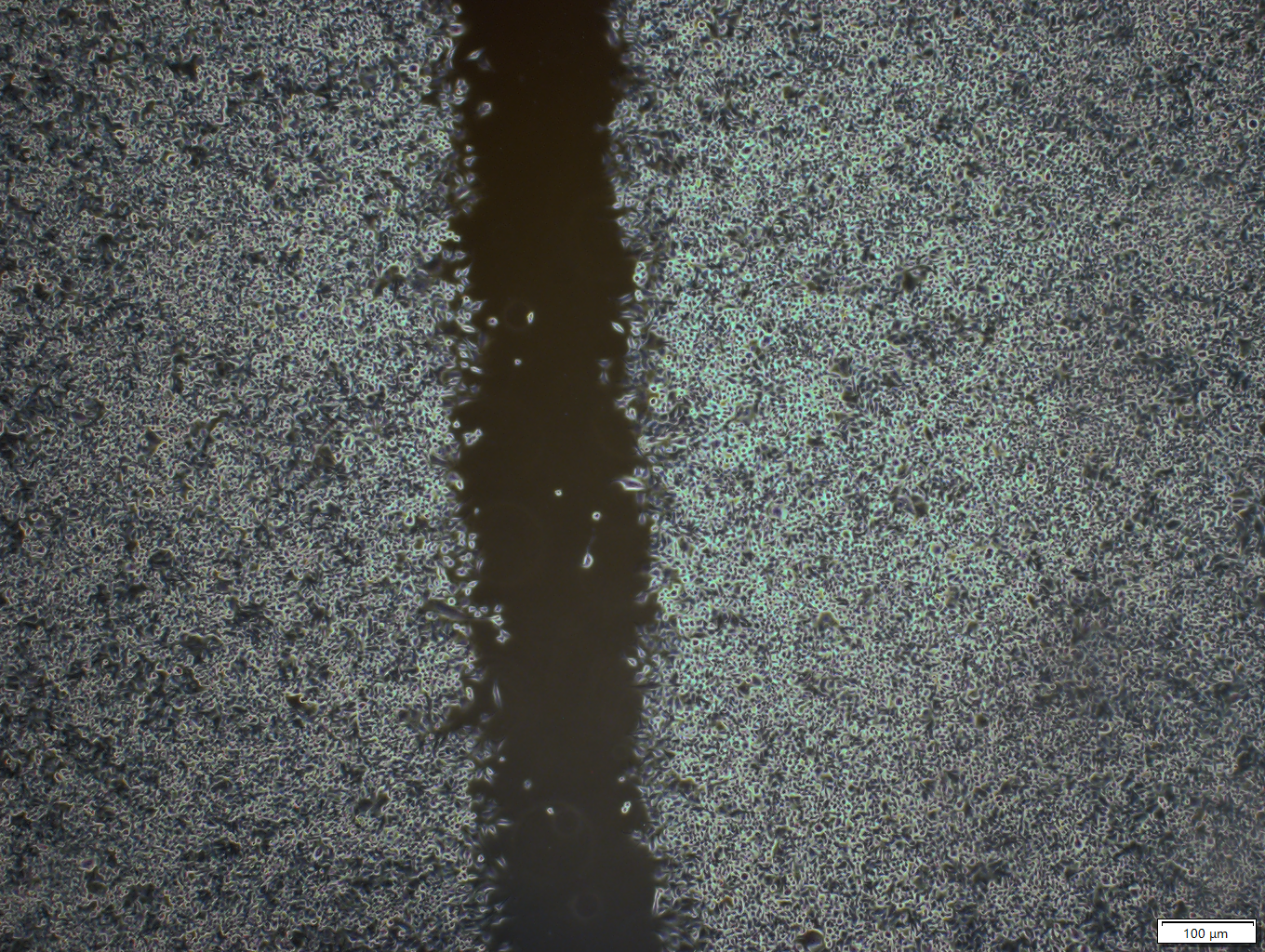

Supplement: S1 File — (ZIP) [file pone.0290753.s001.zip › 22RV1 LV NC 48h.tif]

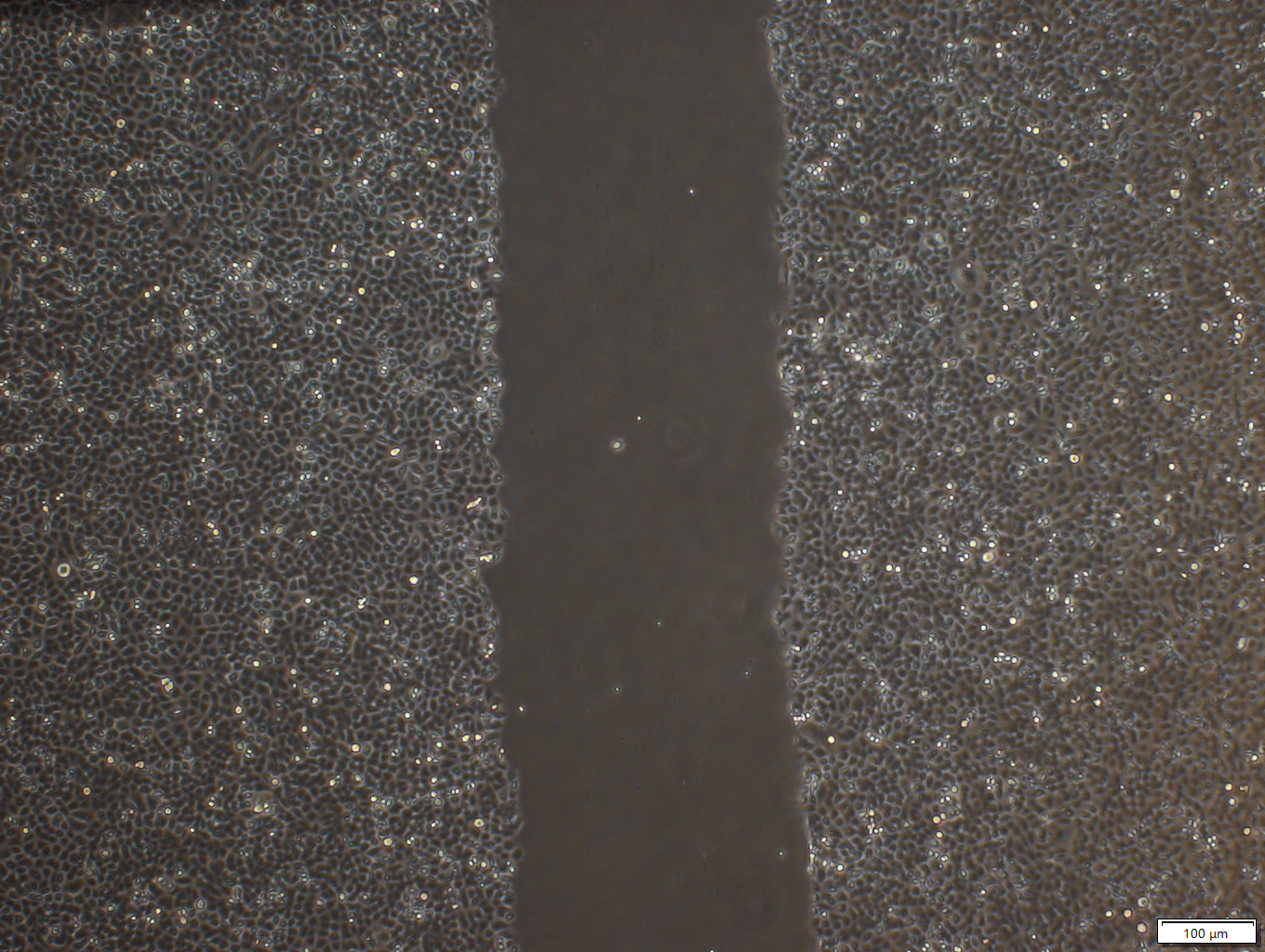

Supplement: S1 File — (ZIP) [file pone.0290753.s001.zip › 22RV1 LV NC.tif]

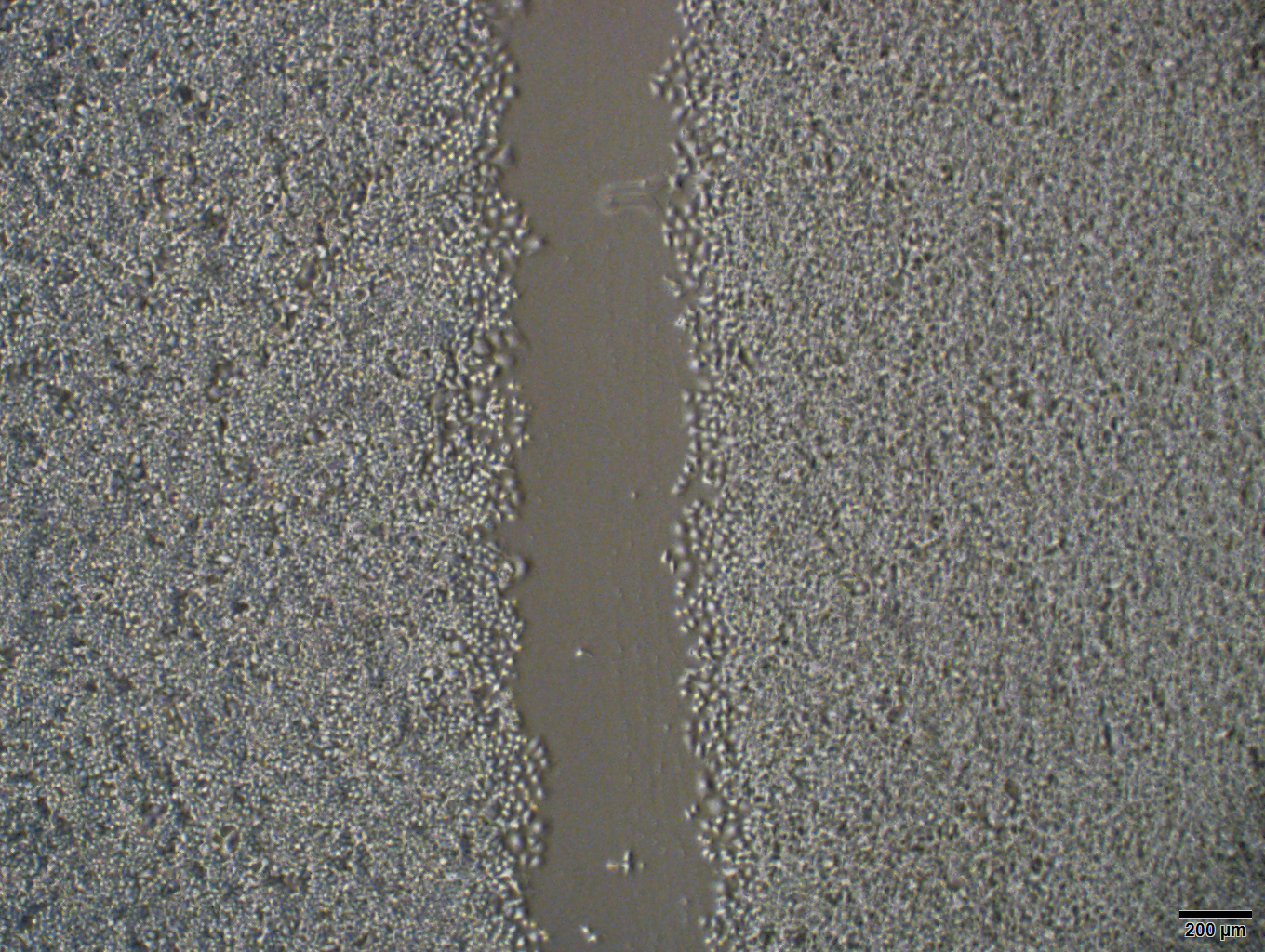

Supplement: S1 File — (ZIP) [file pone.0290753.s001.zip › 22RV1 NC 48H.tif]

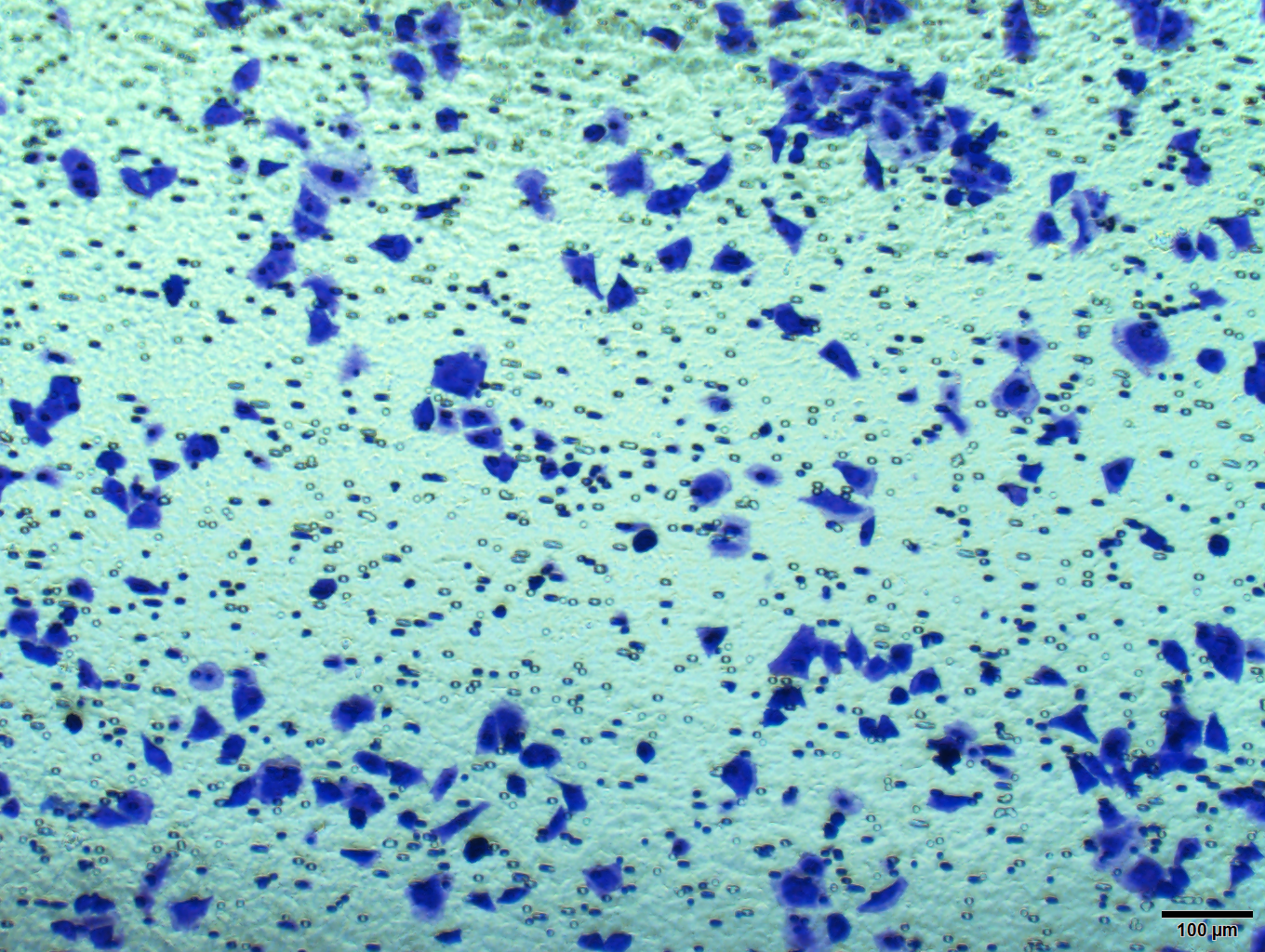

Supplement: S1 File — (ZIP) [file pone.0290753.s001.zip › 22rv1 nc invasion.tif]

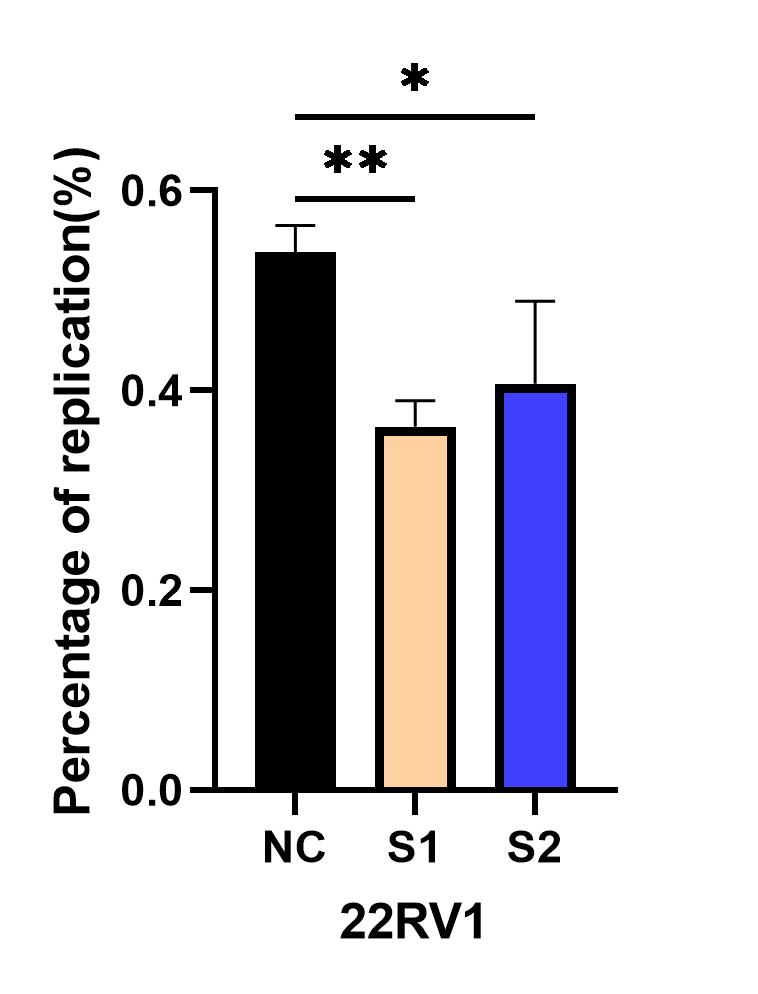

Supplement: S1 File — (ZIP) [file pone.0290753.s001.zip › 22RV1 NC S1 S2 statistical analysis.tif]

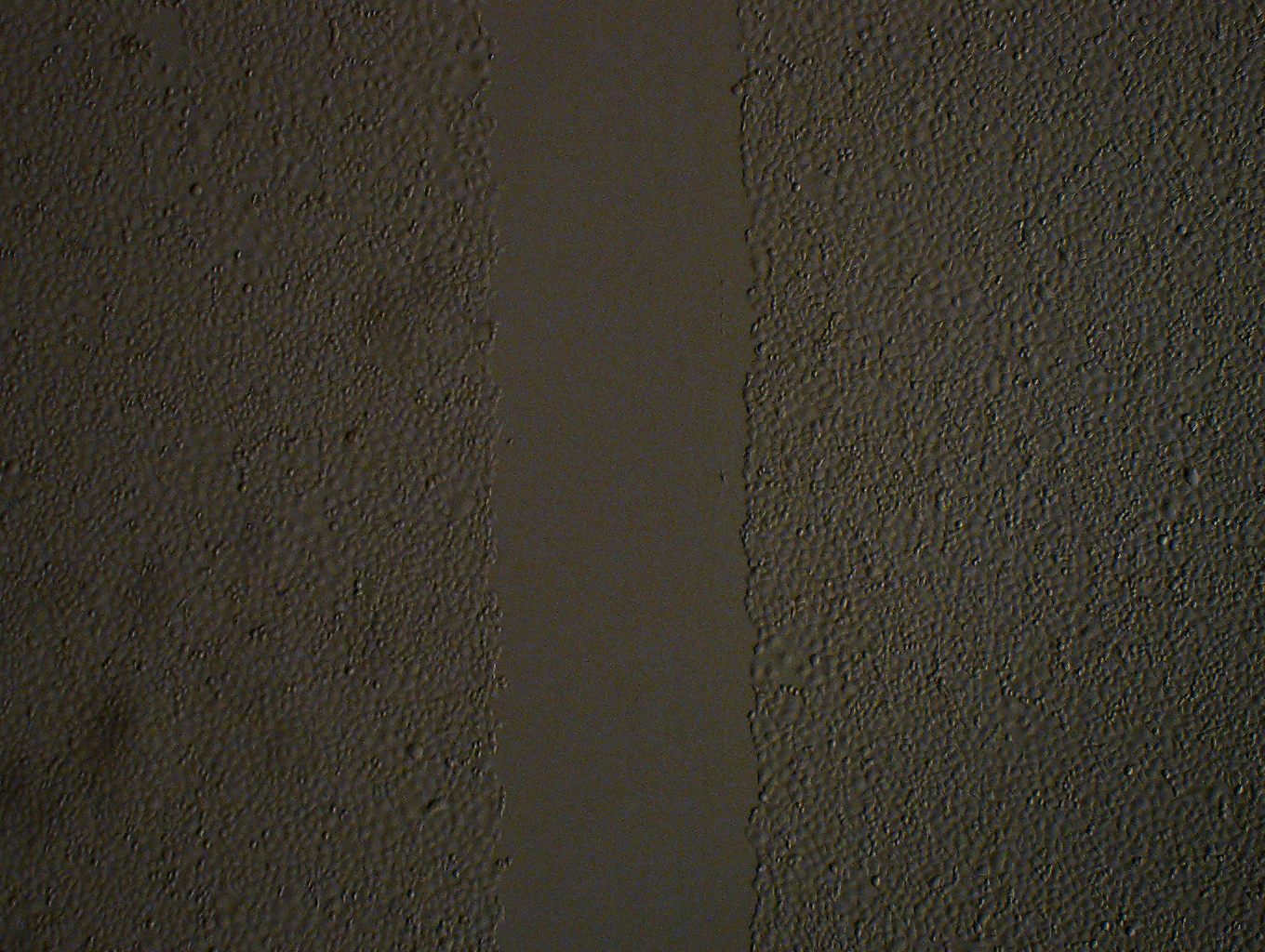

Supplement: S1 File — (ZIP) [file pone.0290753.s001.zip › 22RV1 NC.tif]

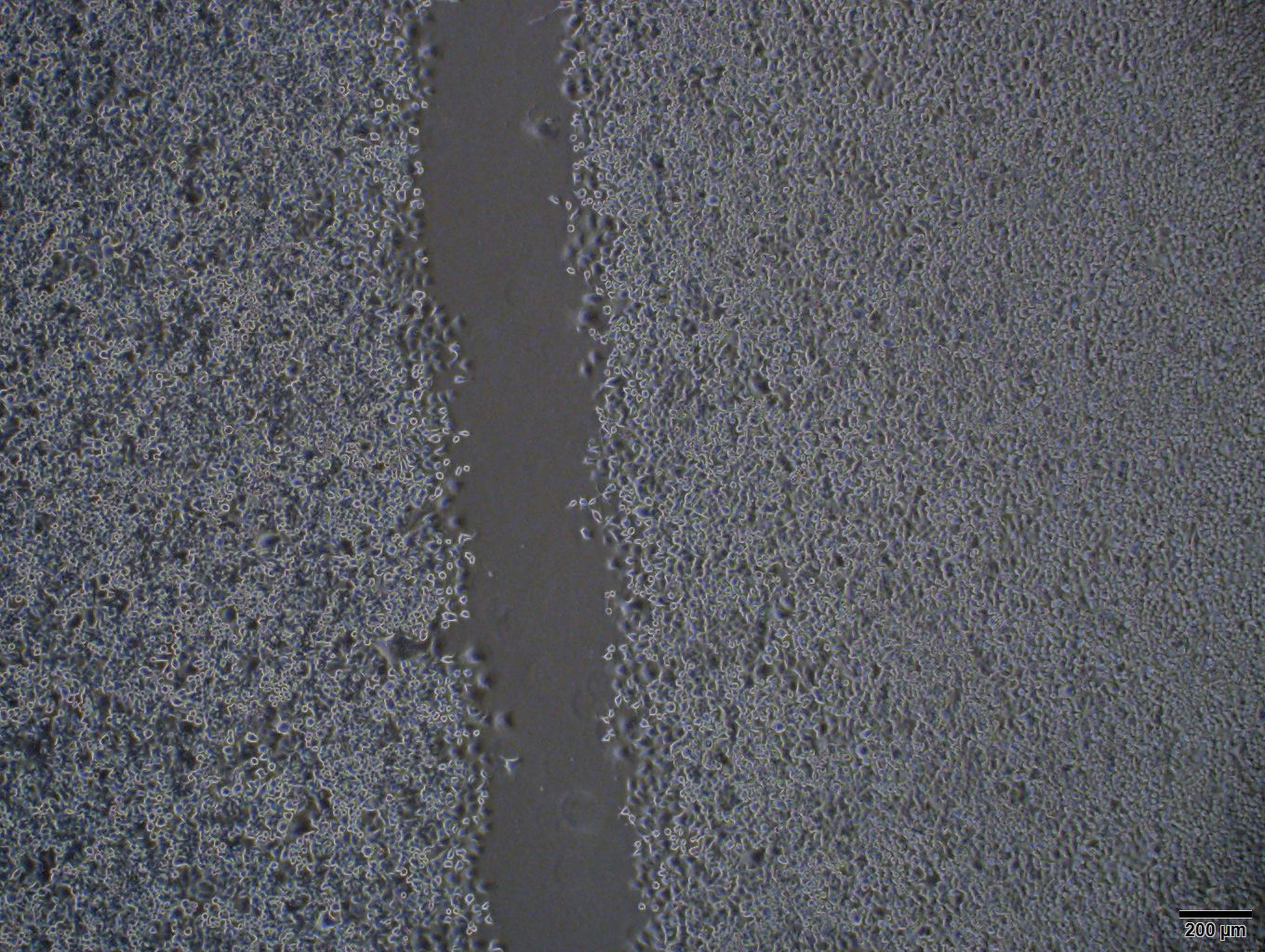

Supplement: S1 File — (ZIP) [file pone.0290753.s001.zip › 22RV1 S1 48H.tif]

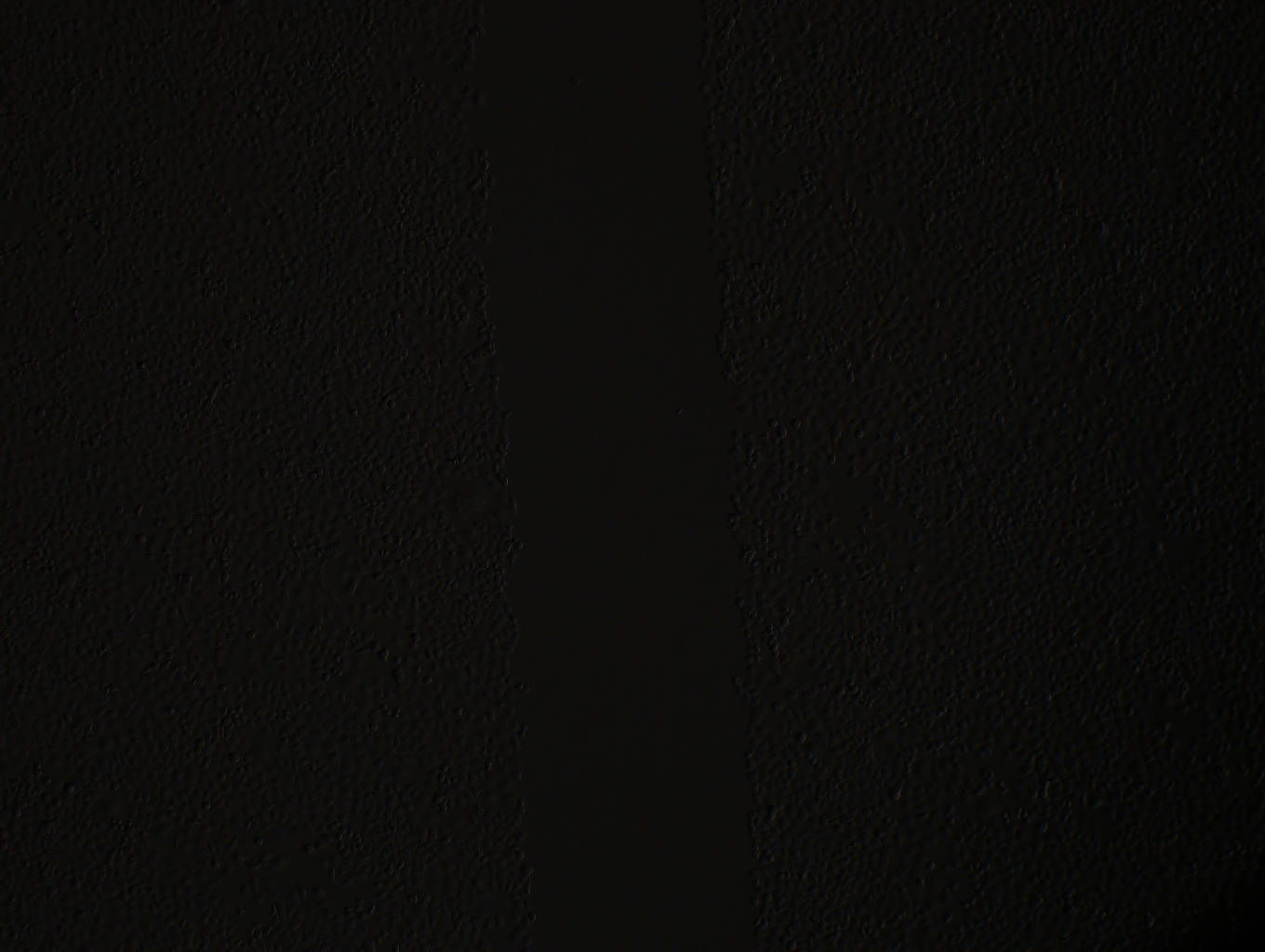

Supplement: S1 File — (ZIP) [file pone.0290753.s001.zip › 22RV1 S1.tif]

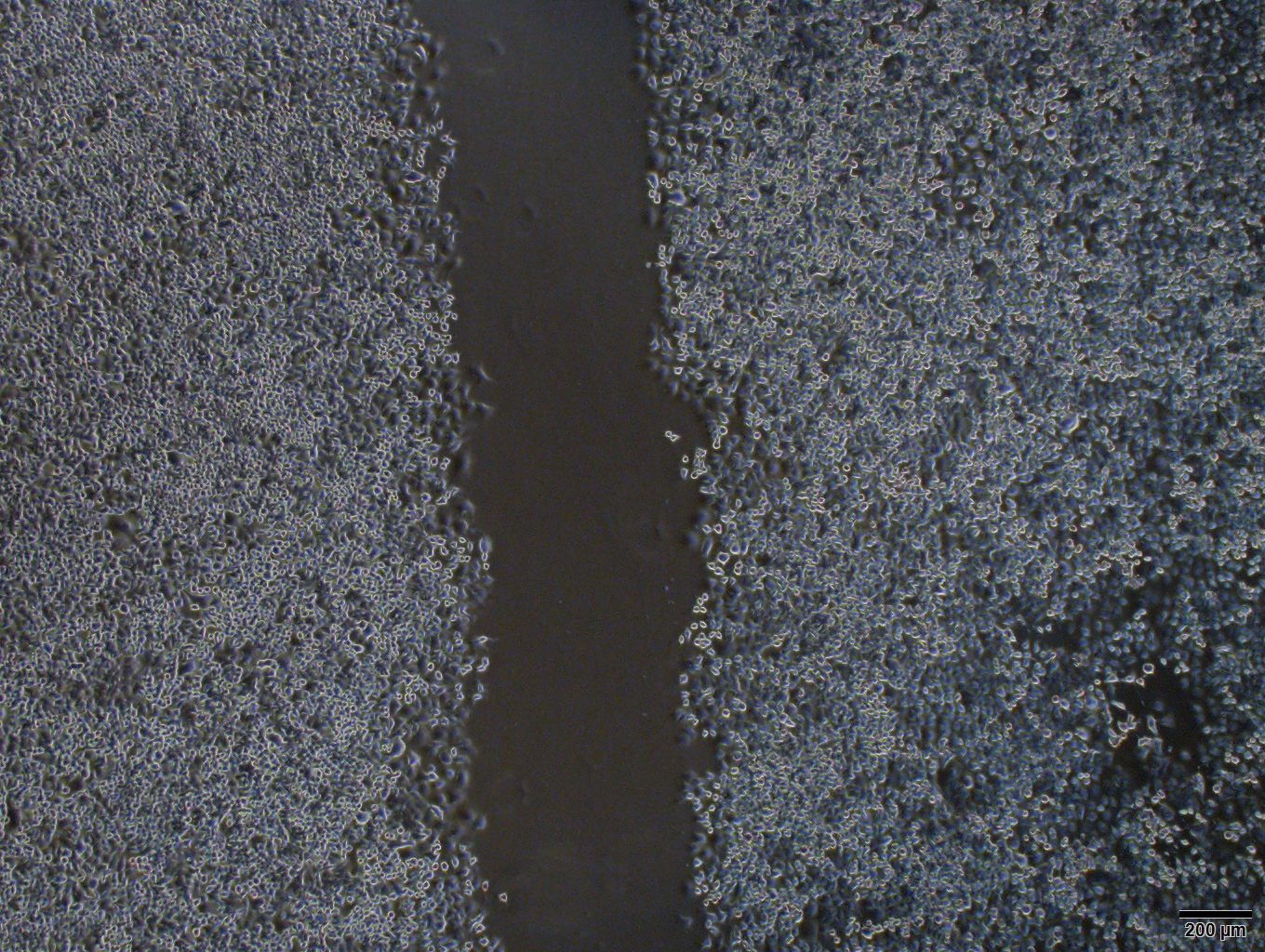

Supplement: S1 File — (ZIP) [file pone.0290753.s001.zip › 22RV1 S2 48H.tif]

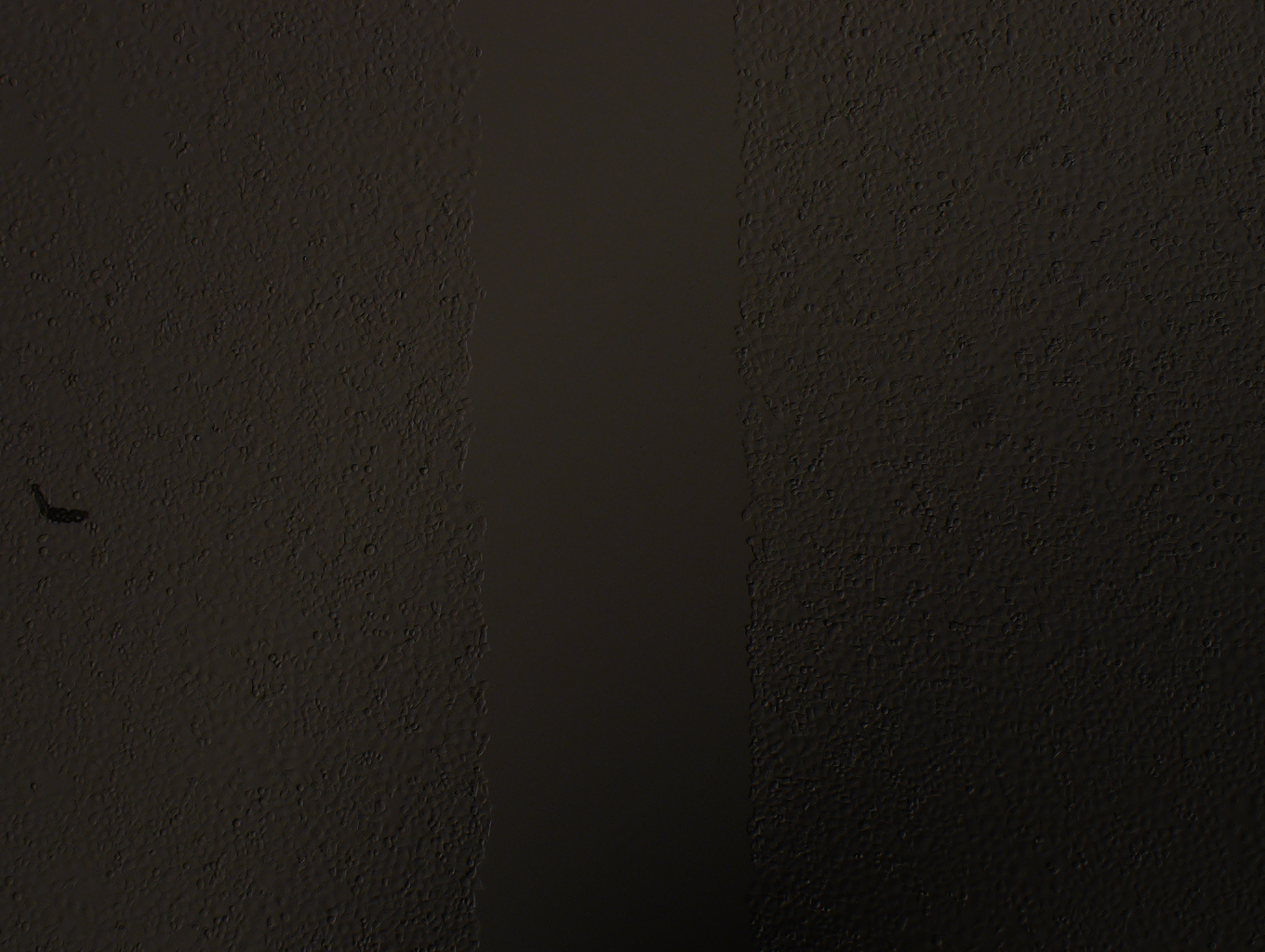

Supplement: S1 File — (ZIP) [file pone.0290753.s001.zip › 22RV1 S2.tif]

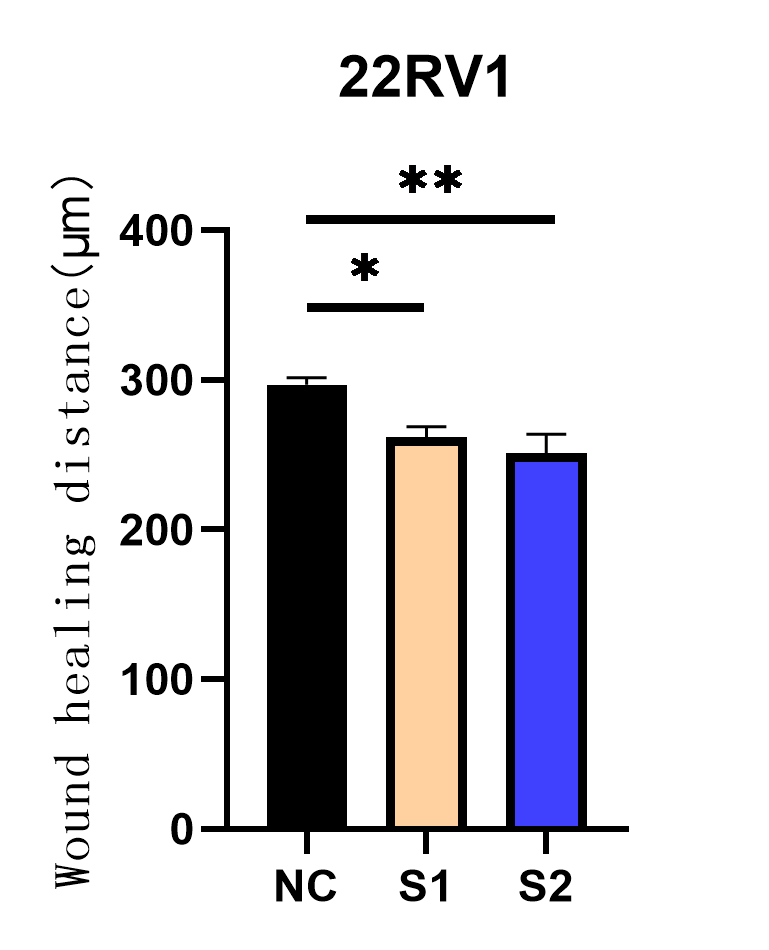

Supplement: S1 File — (ZIP) [file pone.0290753.s001.zip › 22RV1 scratch statistical analysis.tif]

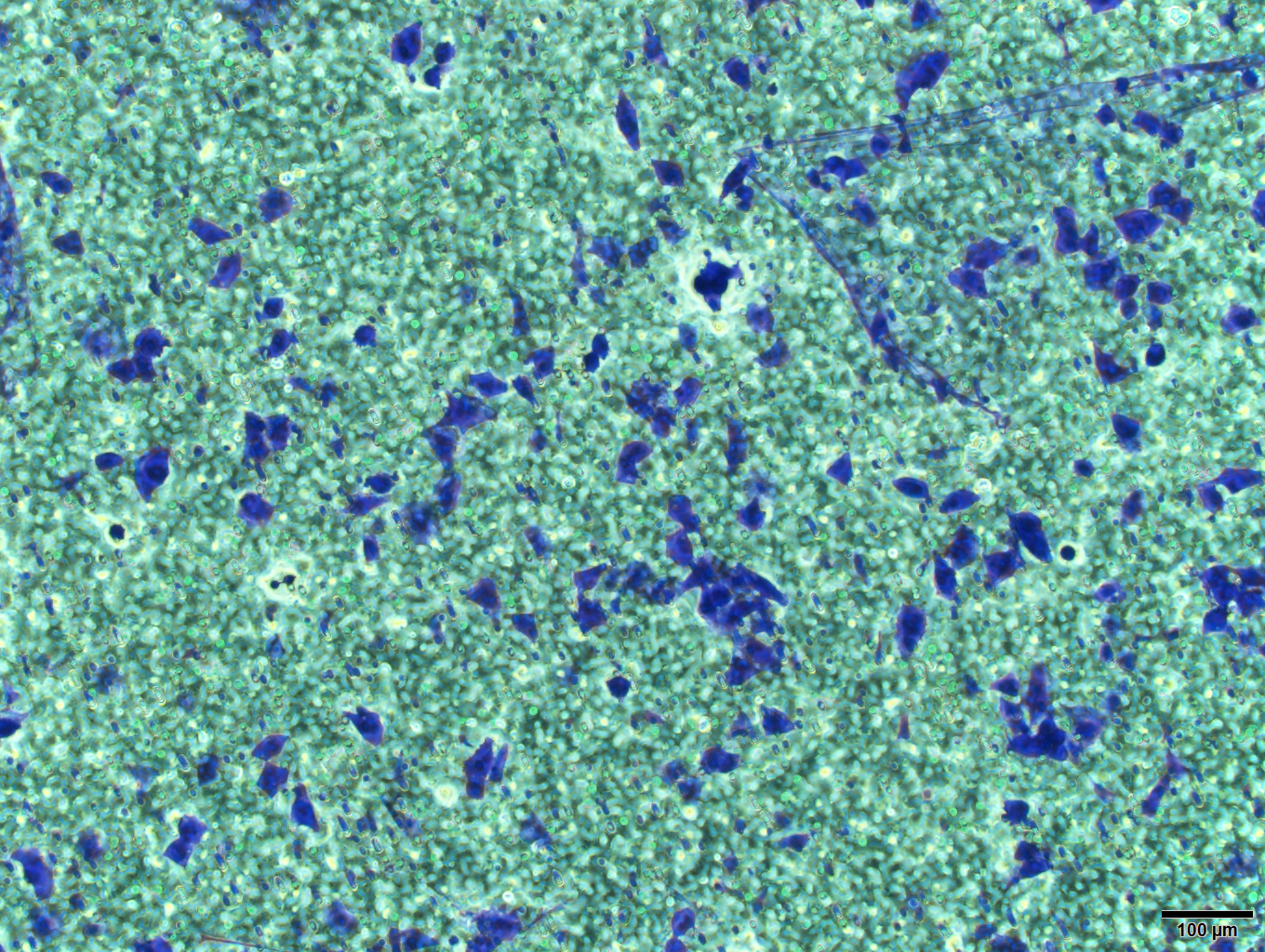

Supplement: S1 File — (ZIP) [file pone.0290753.s001.zip › 22RV1 shPOLG2 invasion.tif]

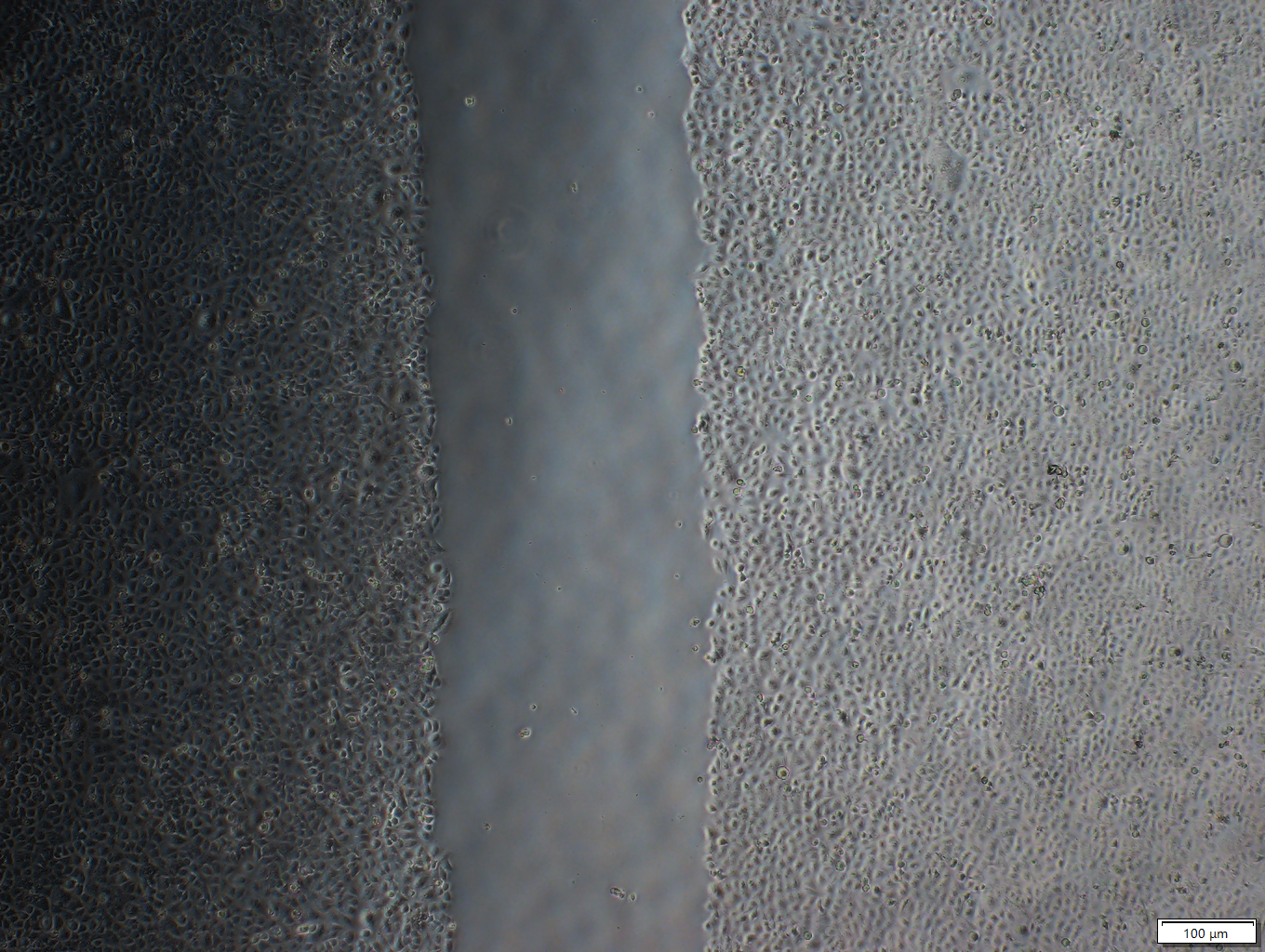

Supplement: S1 File — (ZIP) [file pone.0290753.s001.zip › 22RV1 shPOLG2.tif]

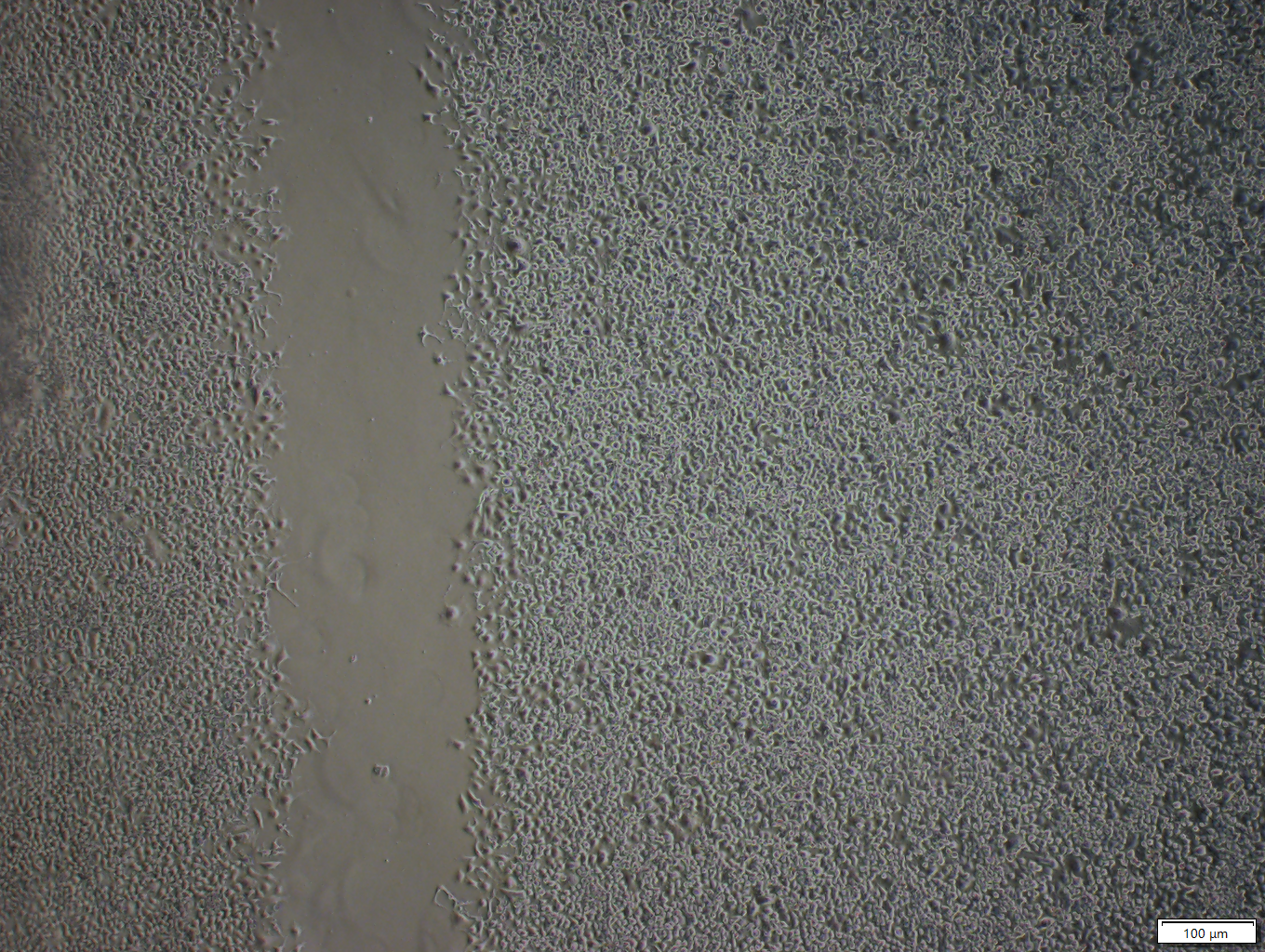

Supplement: S1 File — (ZIP) [file pone.0290753.s001.zip › 22RV1shPOLG2 48h.tif]

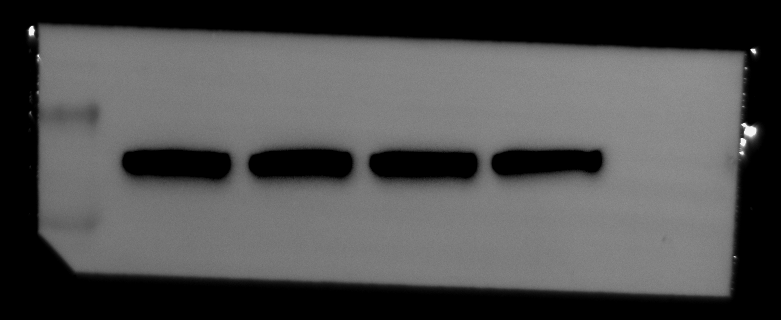

Supplement: S1 File — (ZIP) [file pone.0290753.s001.zip › ACTIN.tif]

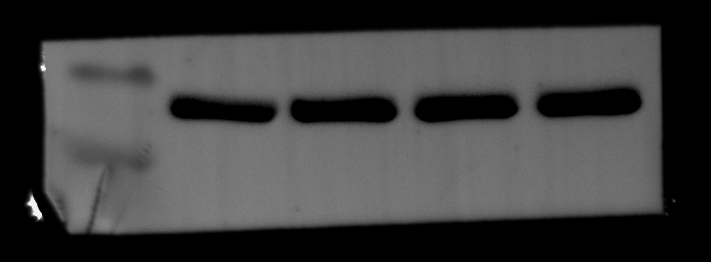

Supplement: S1 File — (ZIP) [file pone.0290753.s001.zip › ACTIN2.tif]

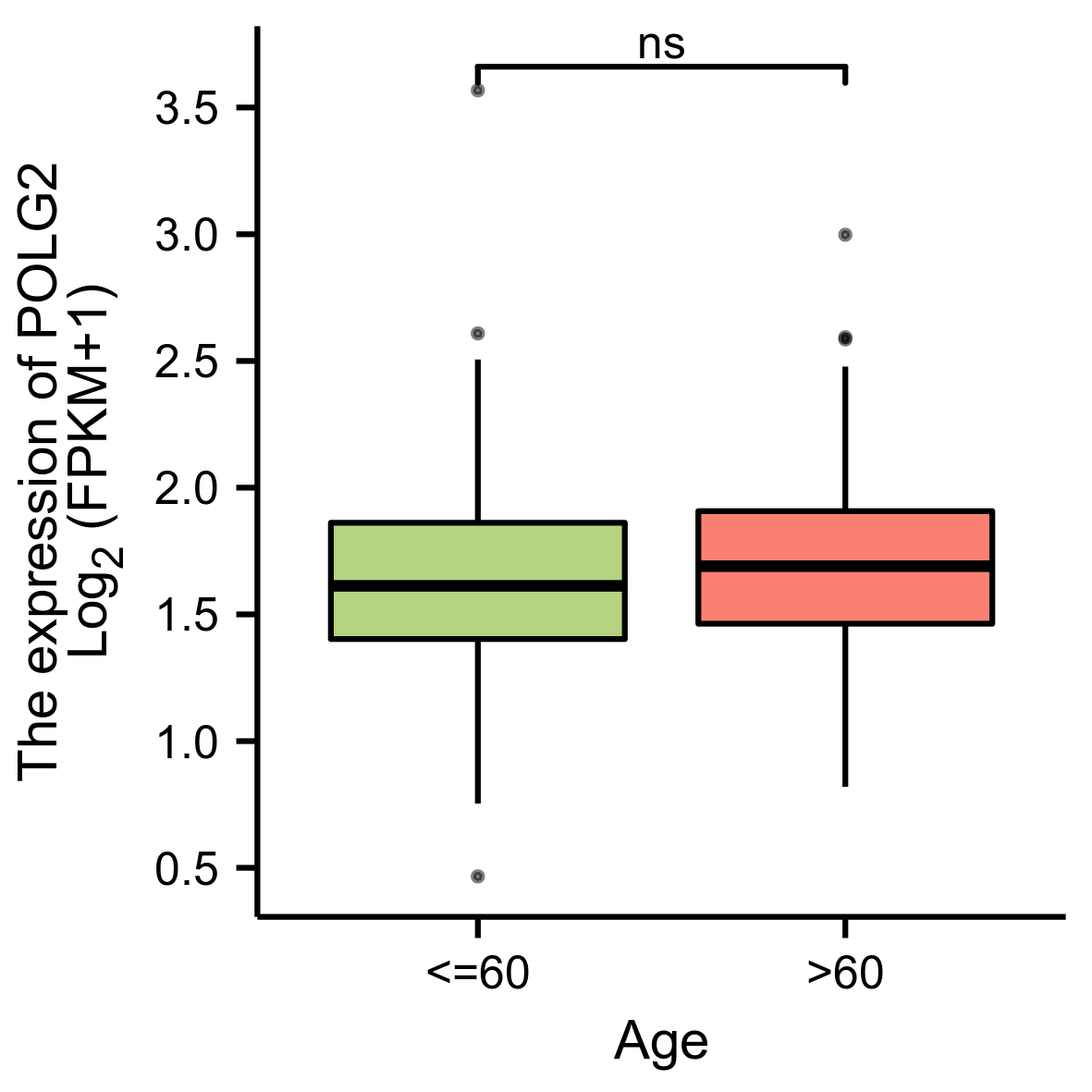

Supplement: S1 File — (ZIP) [file pone.0290753.s001.zip › age .tiff]

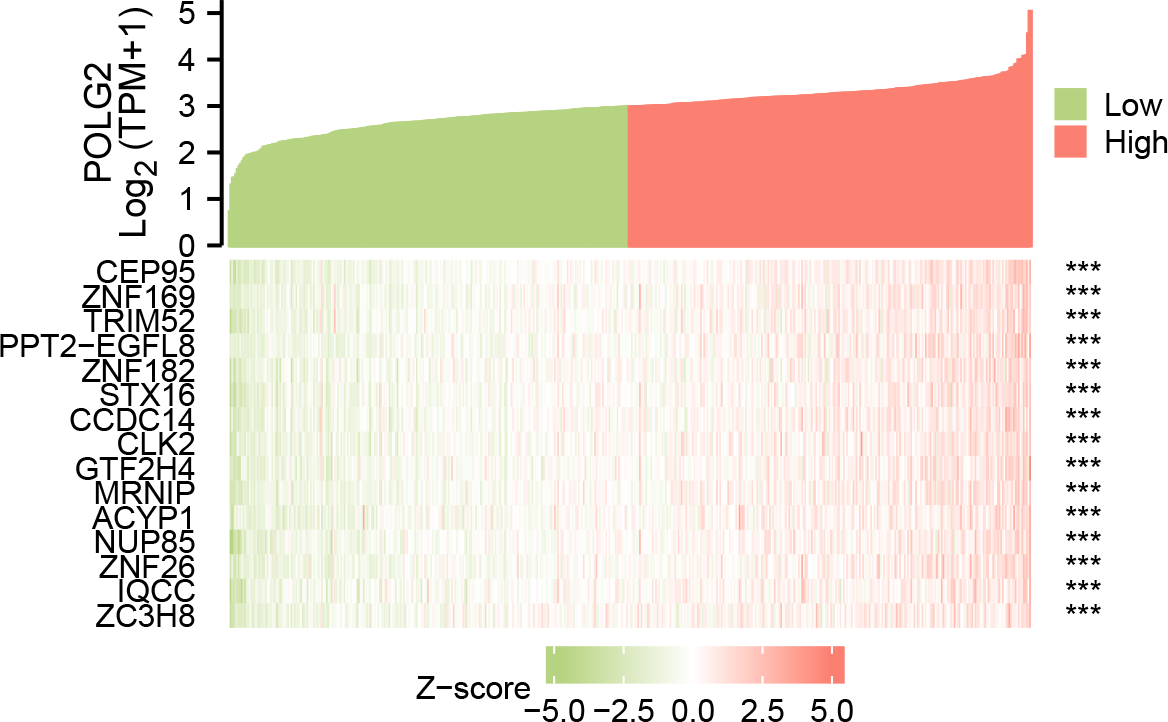

Supplement: S1 File — (ZIP) [file pone.0290753.s001.zip › Co-expression heat map.tif]

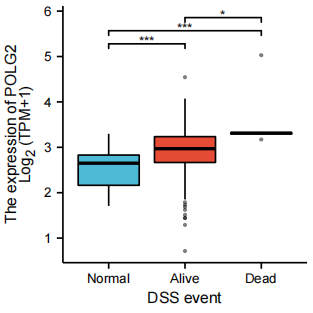

Supplement: S1 File — (ZIP) [file pone.0290753.s001.zip › DSS .tif]

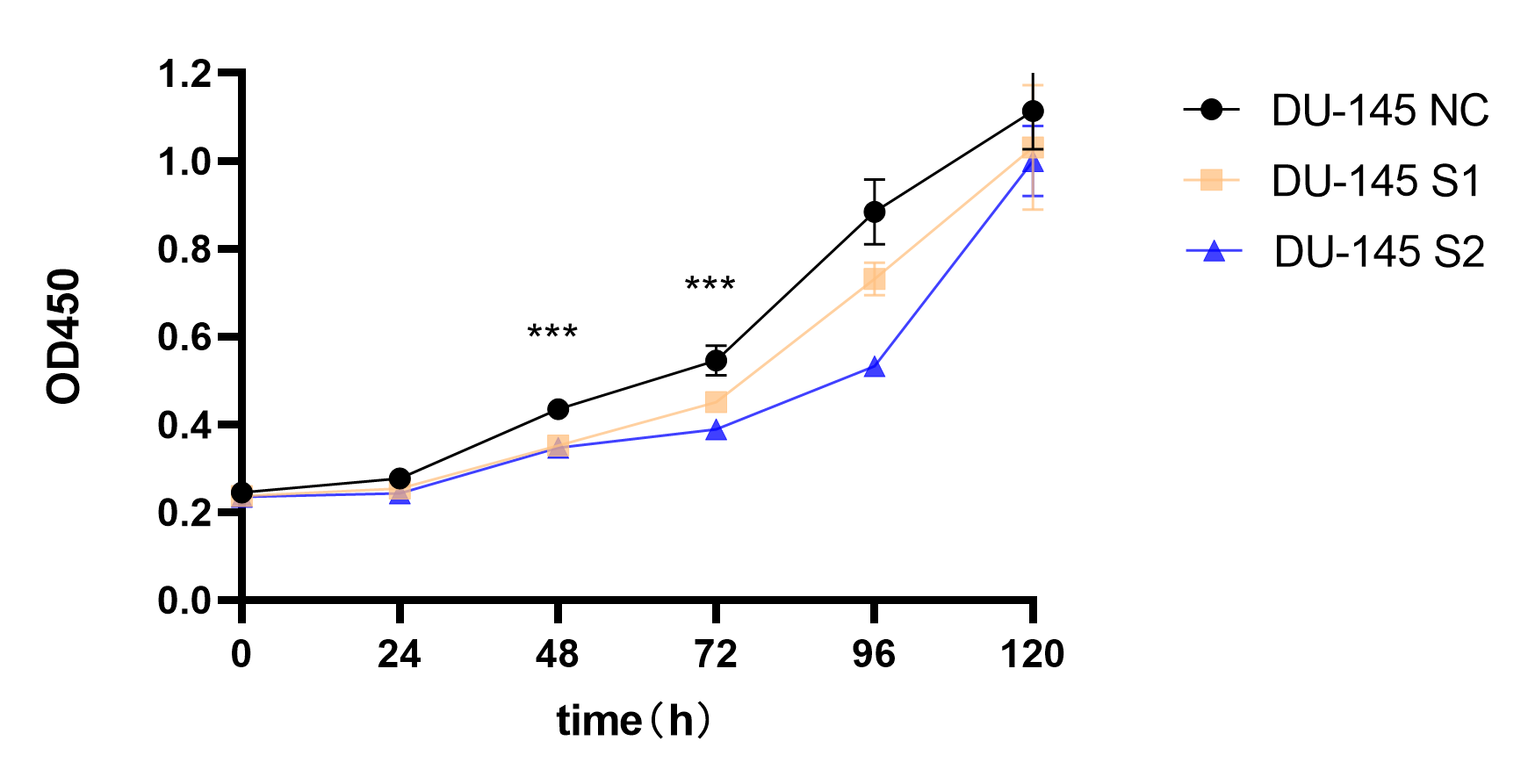

Supplement: S1 File — (ZIP) [file pone.0290753.s001.zip › DU-145 CCK-8.tif]

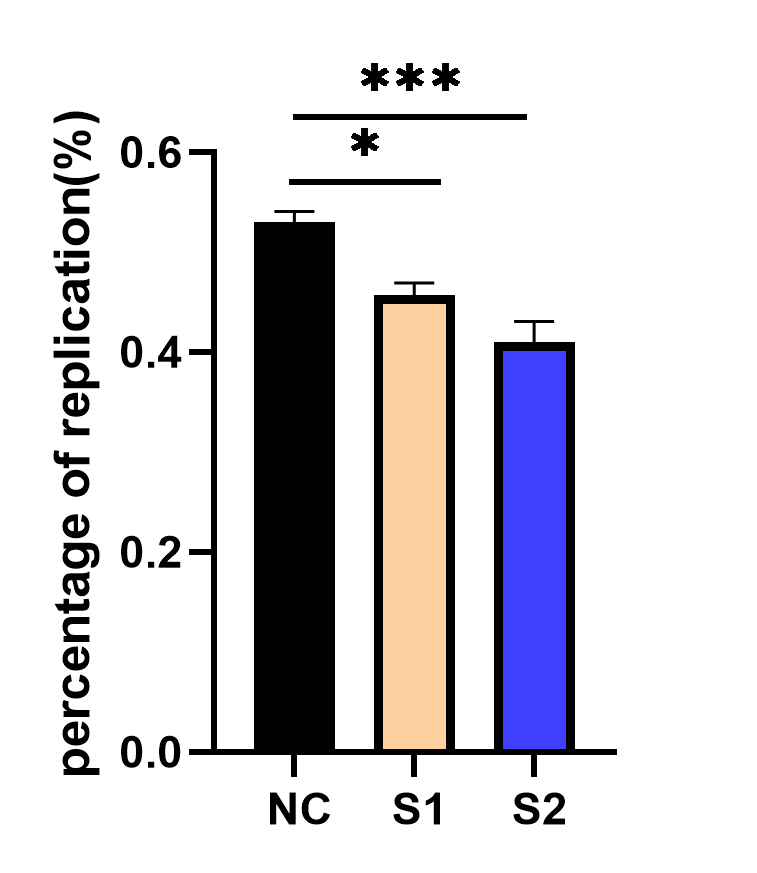

Supplement: S1 File — (ZIP) [file pone.0290753.s001.zip › DU-145 EDU NC S1 S2 statistical analysis.tif]

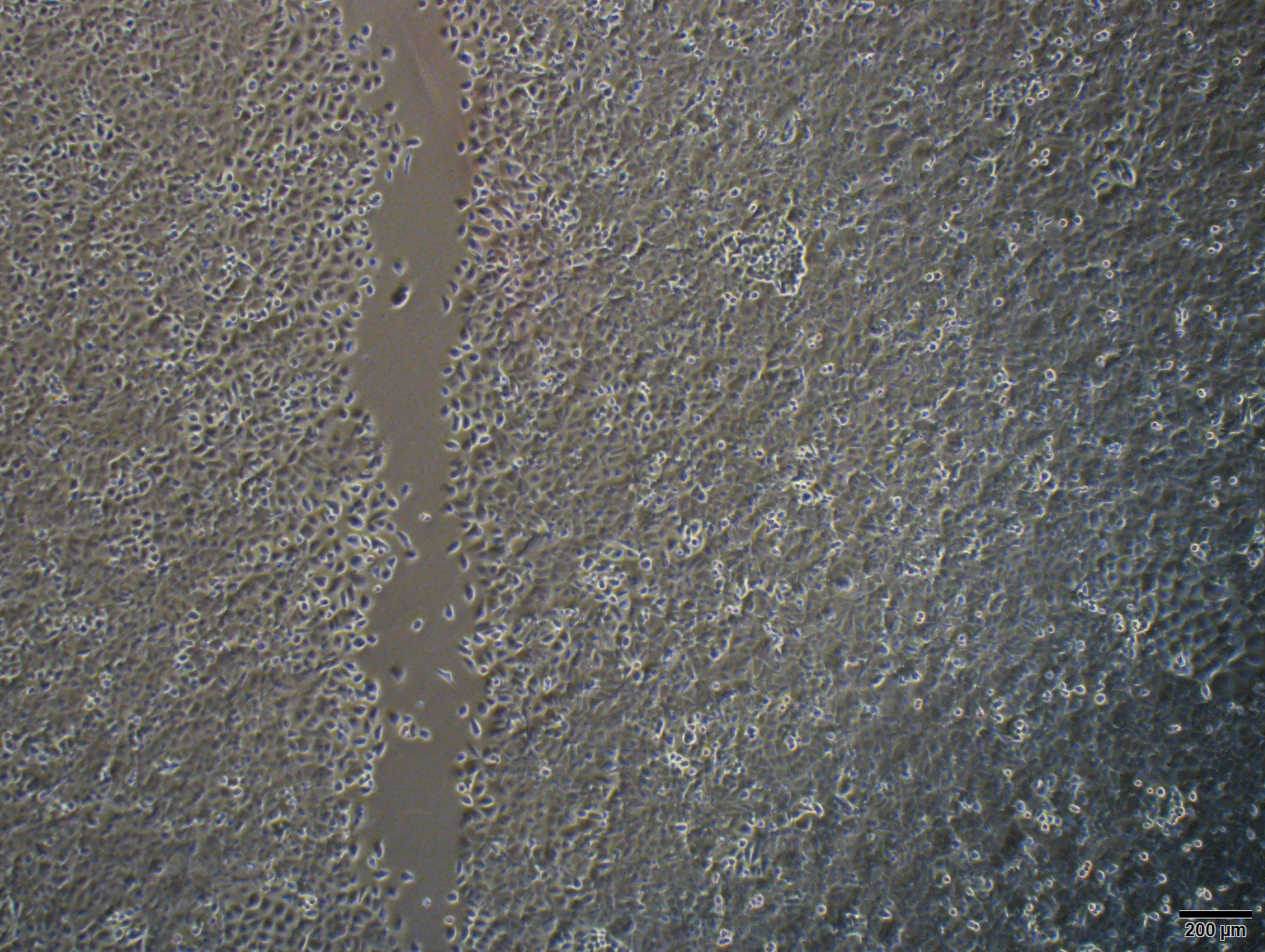

Supplement: S1 File — (ZIP) [file pone.0290753.s001.zip › DU-145 NC 48H.tif]

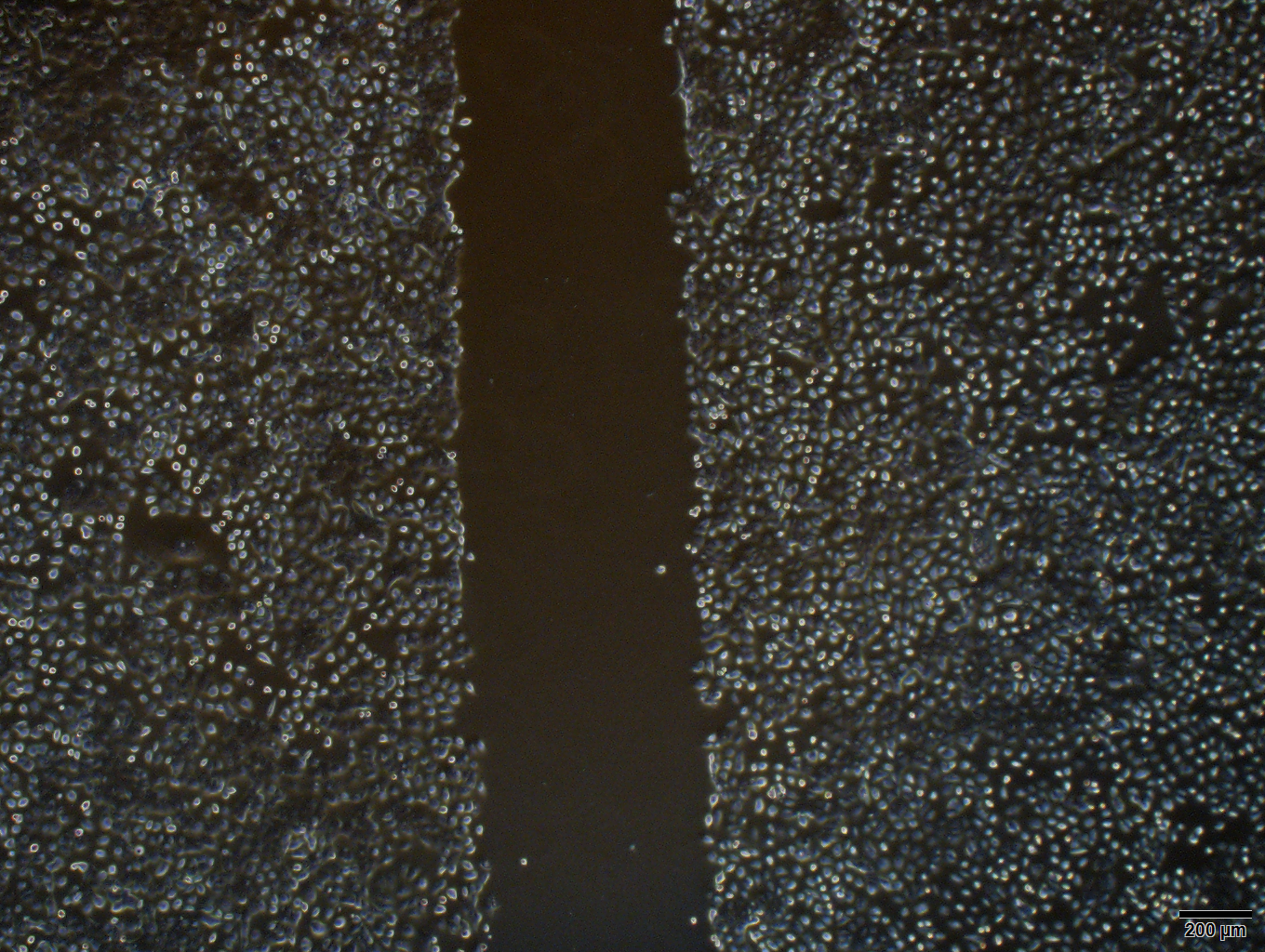

Supplement: S1 File — (ZIP) [file pone.0290753.s001.zip › DU-145 NC.tif]

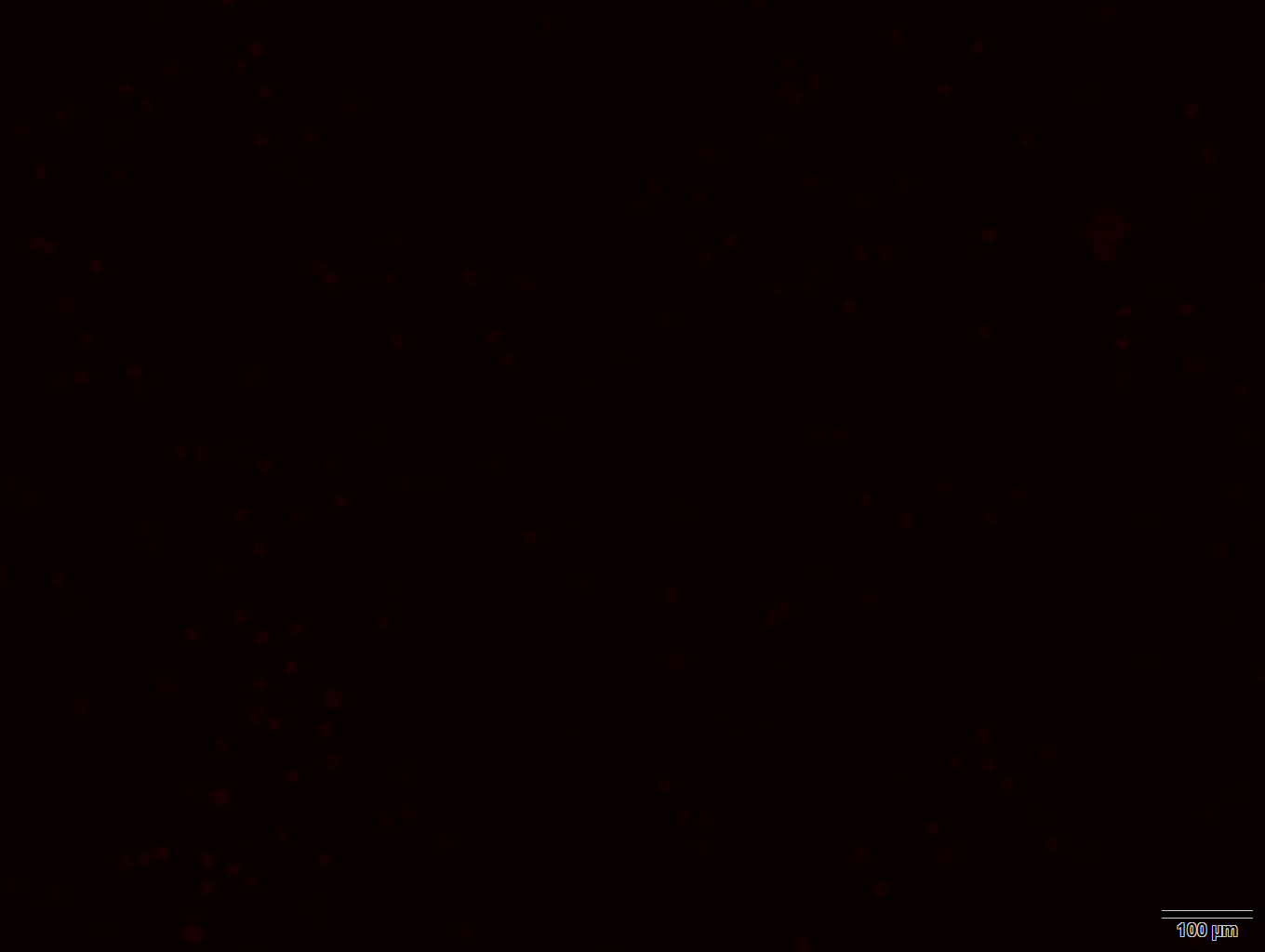

Supplement: S1 File — (ZIP) [file pone.0290753.s001.zip › du-145 NC_0001.tif]

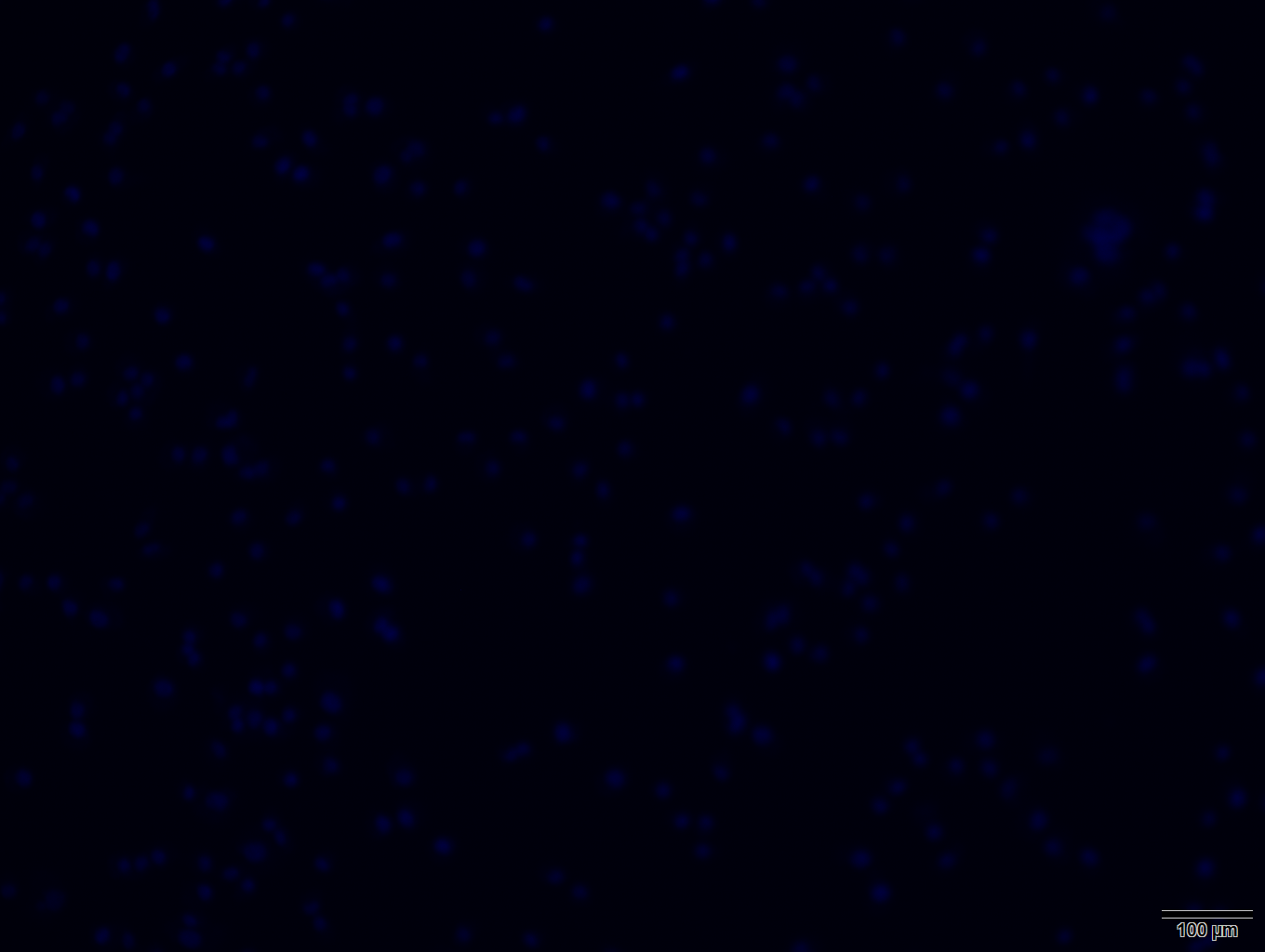

Supplement: S1 File — (ZIP) [file pone.0290753.s001.zip › du-145 NC_0002.tif]

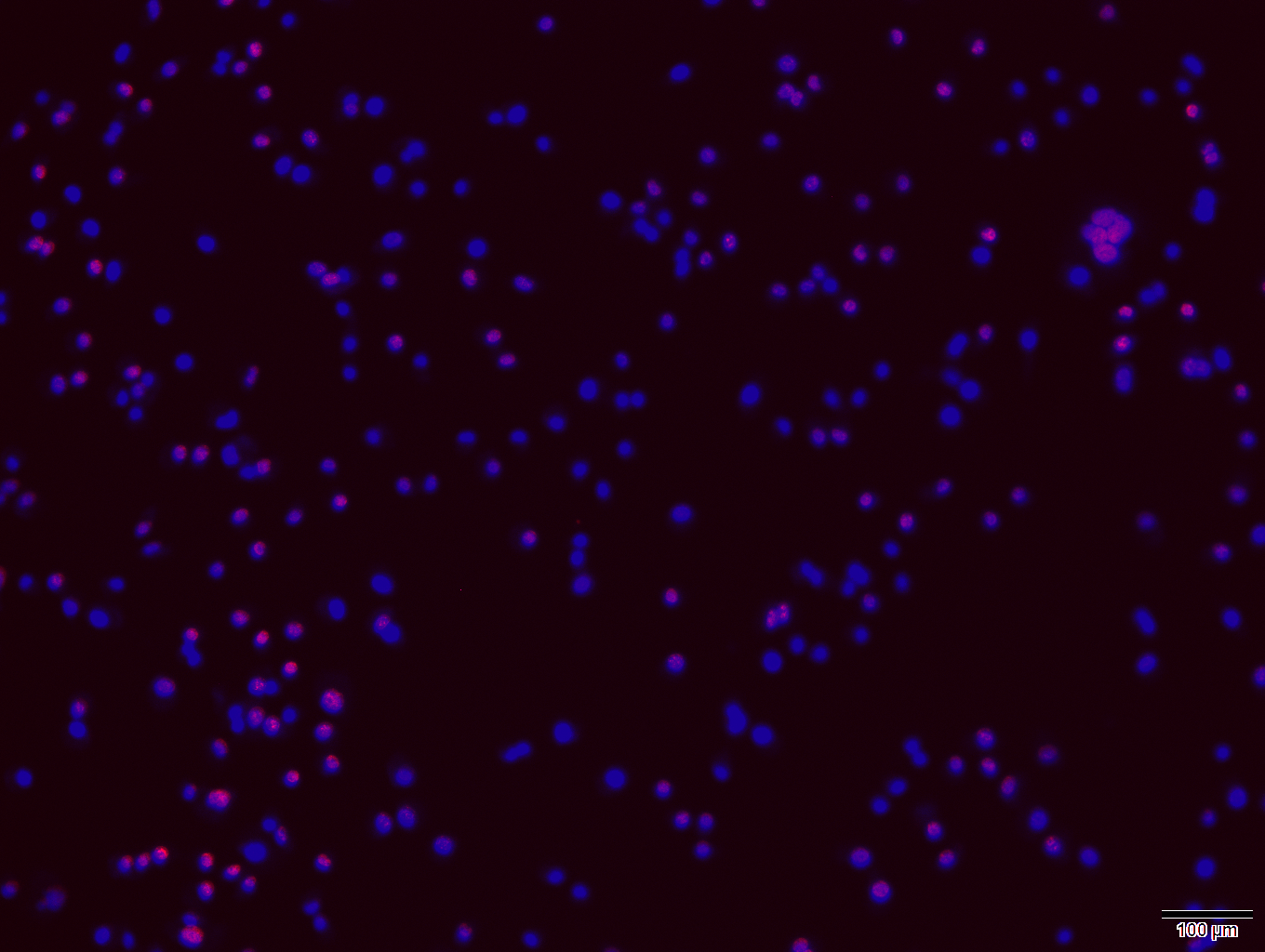

Supplement: S1 File — (ZIP) [file pone.0290753.s001.zip › du-145 NC_1 2 merge.tif]

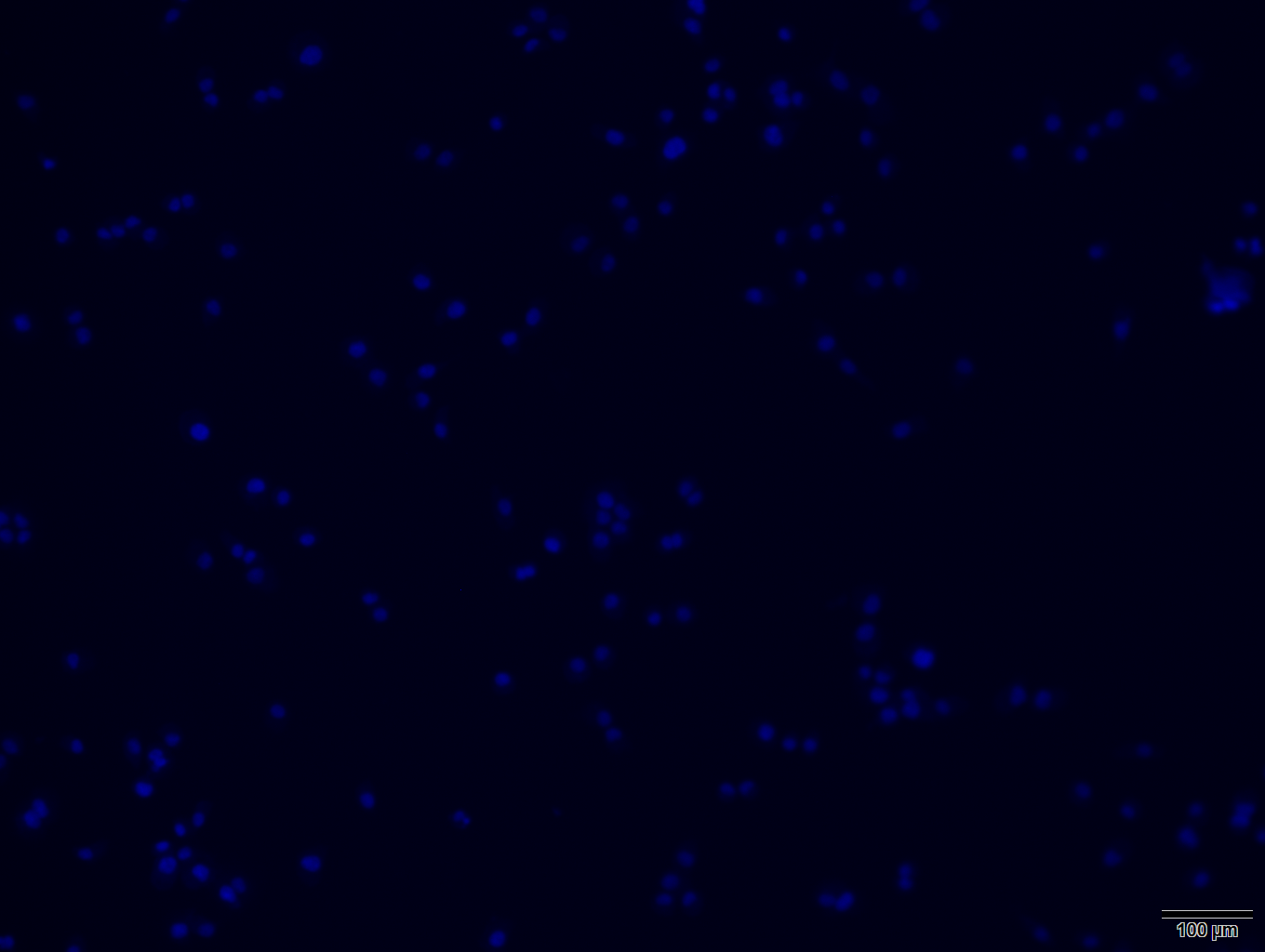

Supplement: S1 File — (ZIP) [file pone.0290753.s001.zip › du-145 S1 0011.tif]

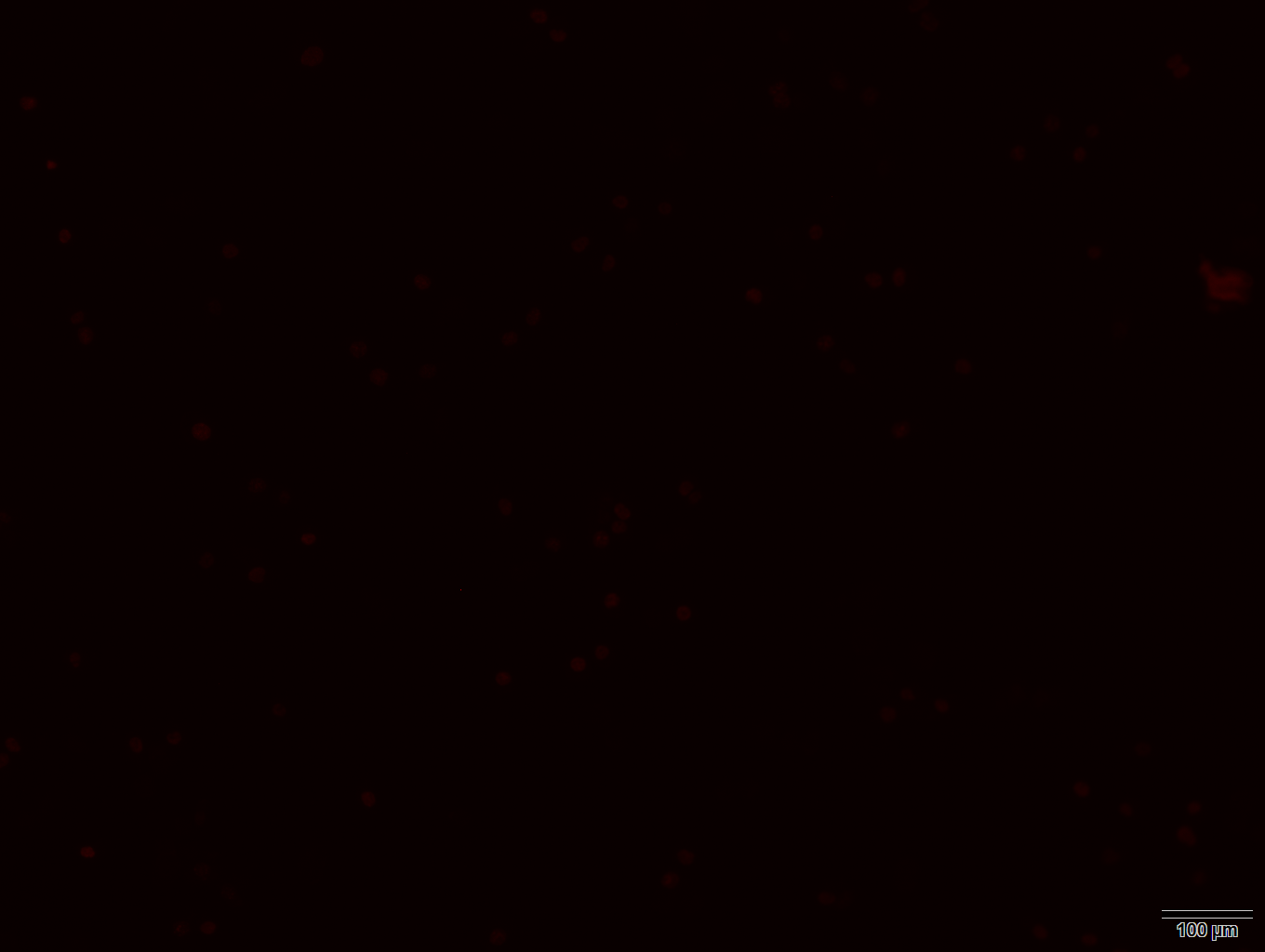

Supplement: S1 File — (ZIP) [file pone.0290753.s001.zip › du-145 S1 0012.tif]

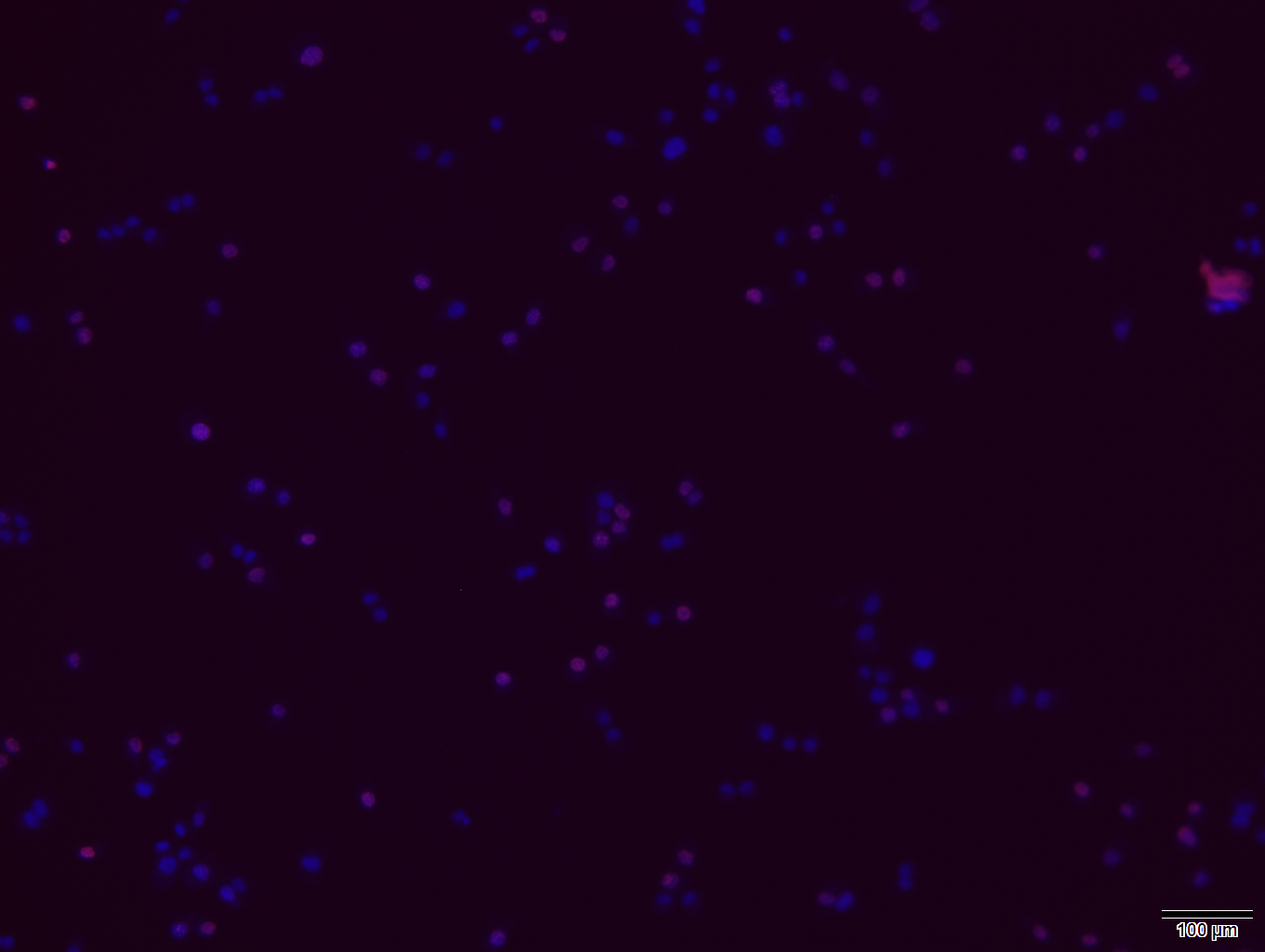

Supplement: S1 File — (ZIP) [file pone.0290753.s001.zip › du-145 S1 11 12merge .tif]

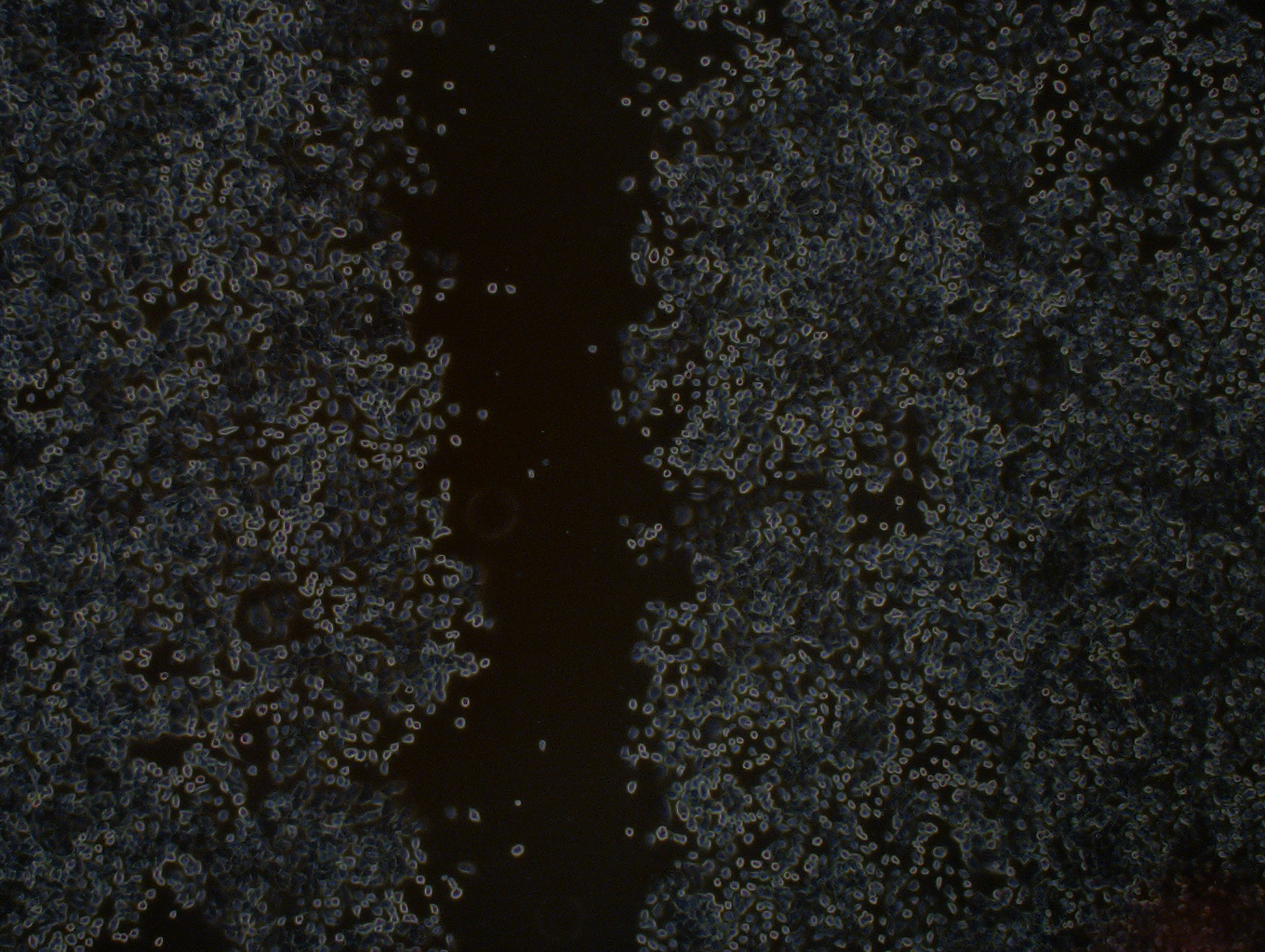

Supplement: S1 File — (ZIP) [file pone.0290753.s001.zip › DU-145 S1 48H.tif]

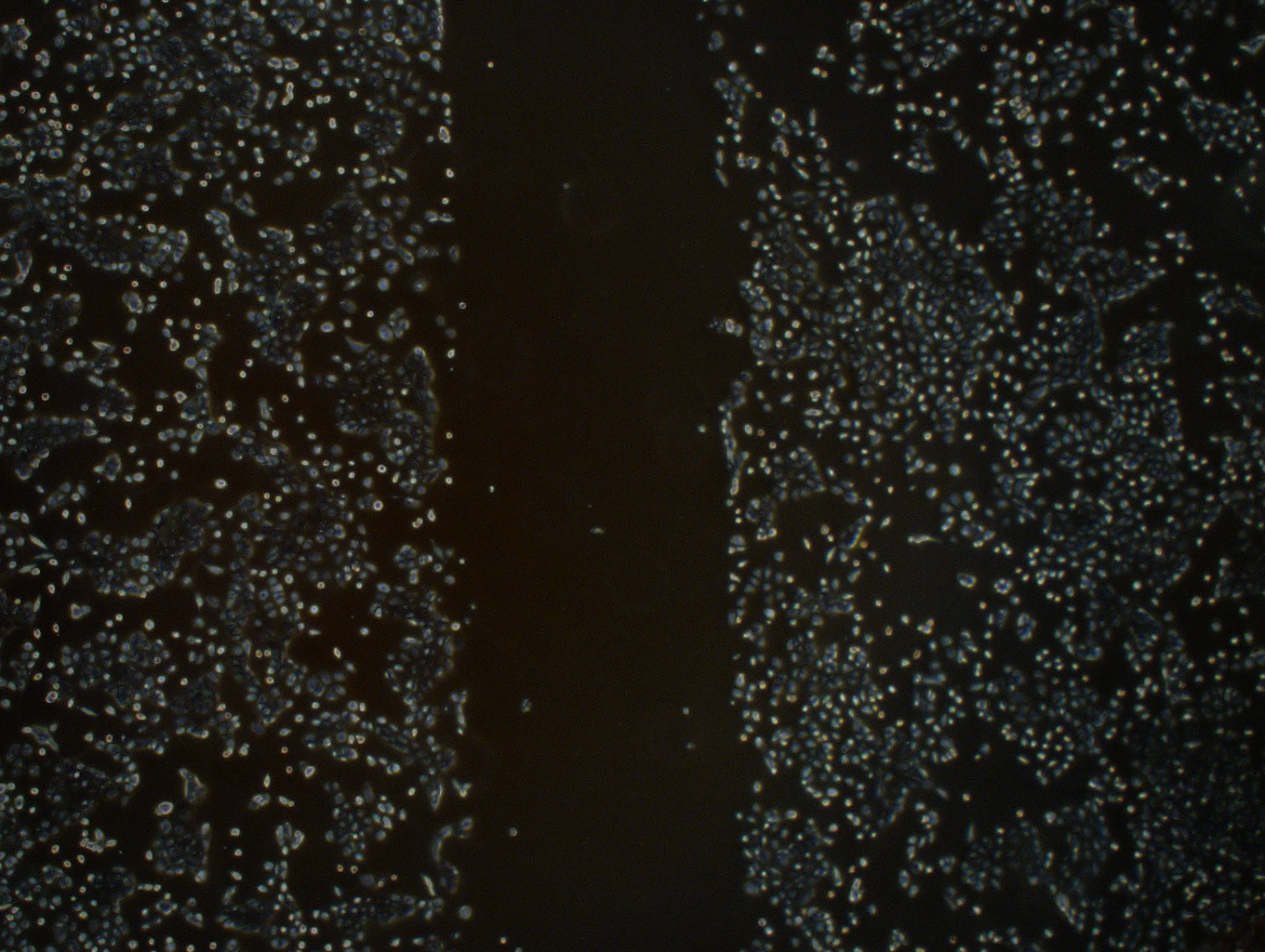

Supplement: S1 File — (ZIP) [file pone.0290753.s001.zip › DU-145 S1.tif]

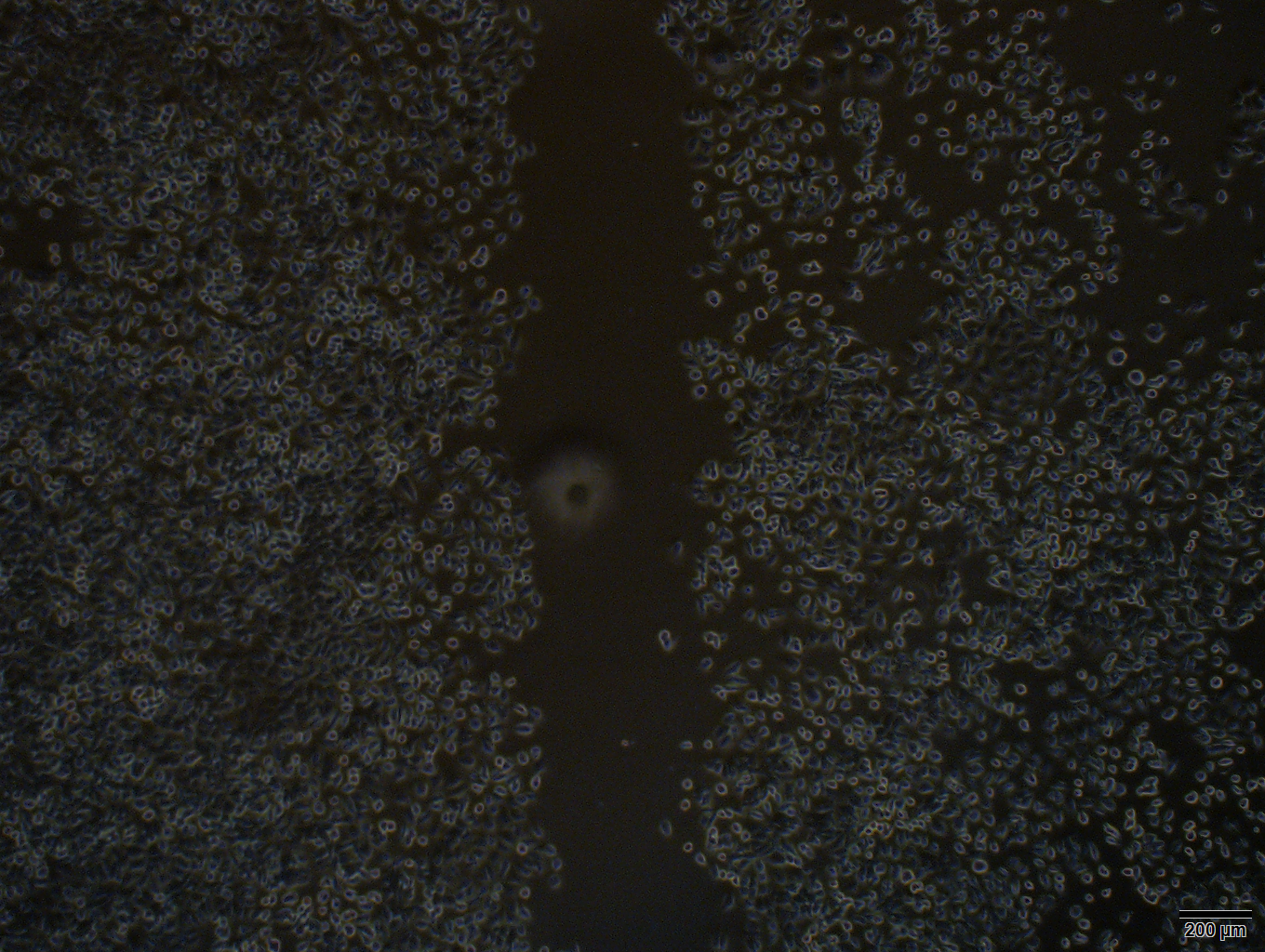

Supplement: S1 File — (ZIP) [file pone.0290753.s001.zip › DU-145 S2 48H.tif]

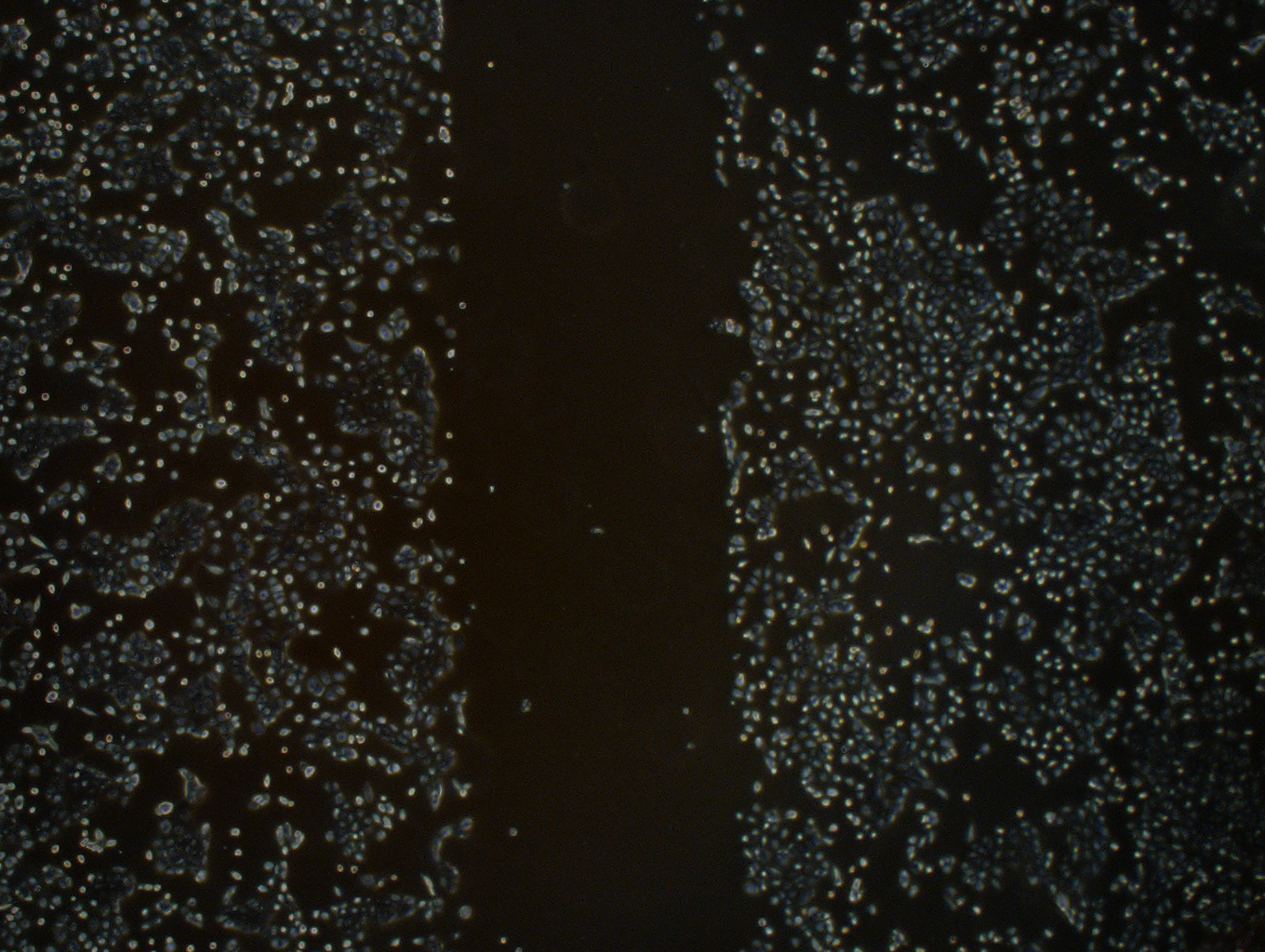

Supplement: S1 File — (ZIP) [file pone.0290753.s001.zip › DU-145 S2.tif]

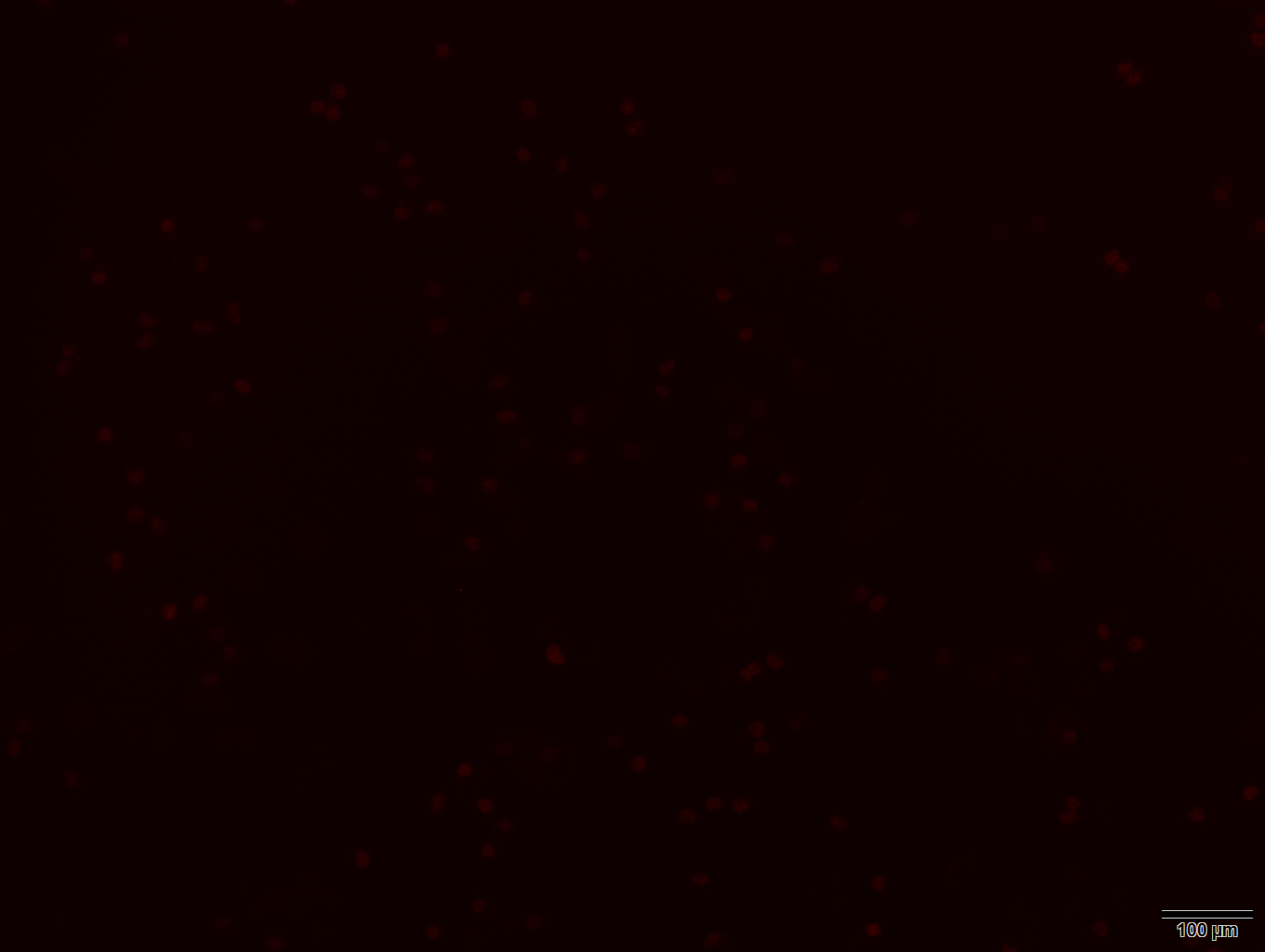

Supplement: S1 File — (ZIP) [file pone.0290753.s001.zip › du-145 S2_0023.tif]

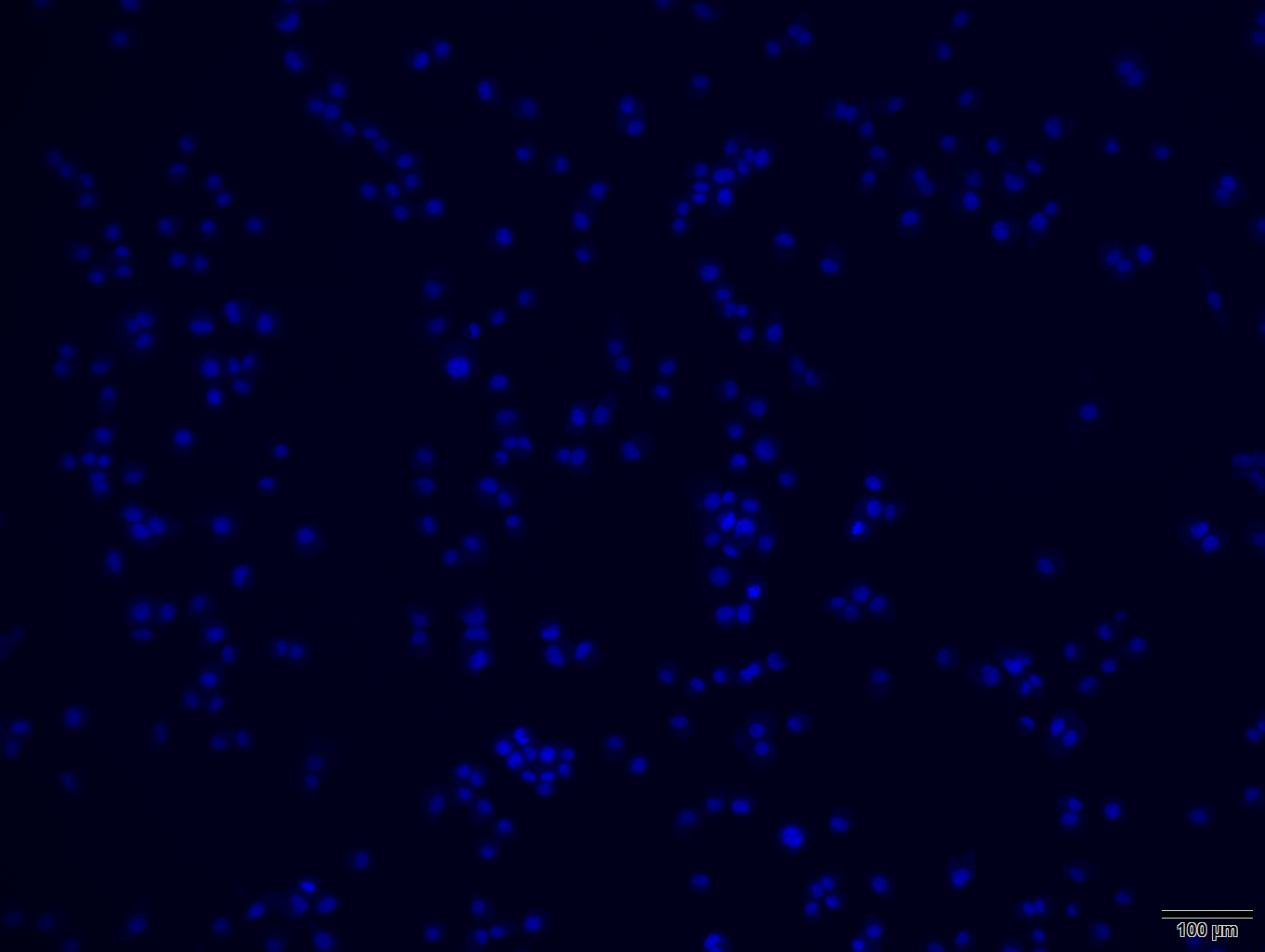

Supplement: S1 File — (ZIP) [file pone.0290753.s001.zip › du-145 S2_0024.tif]

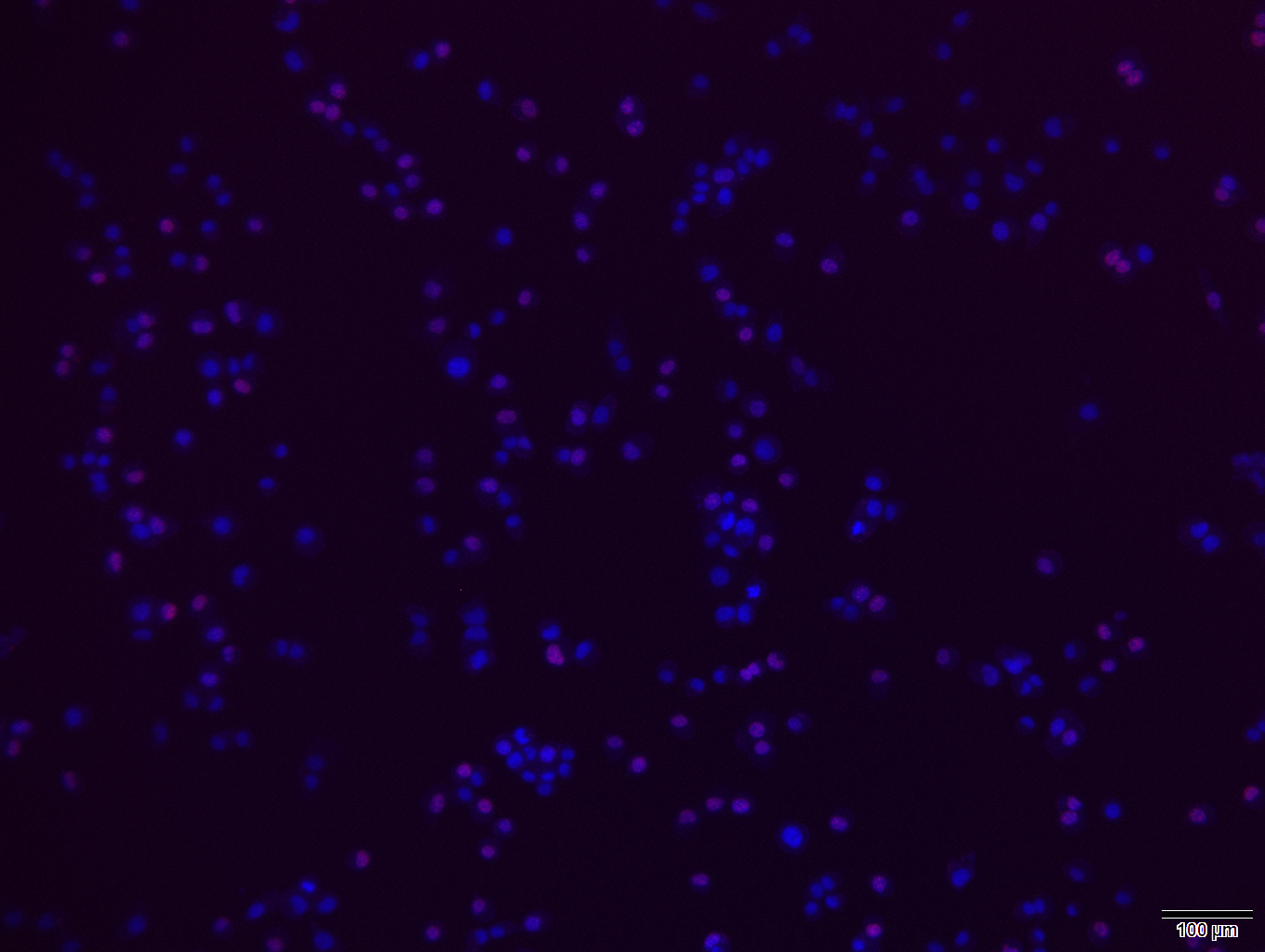

Supplement: S1 File — (ZIP) [file pone.0290753.s001.zip › du-145 S2_23 24merge .tif]

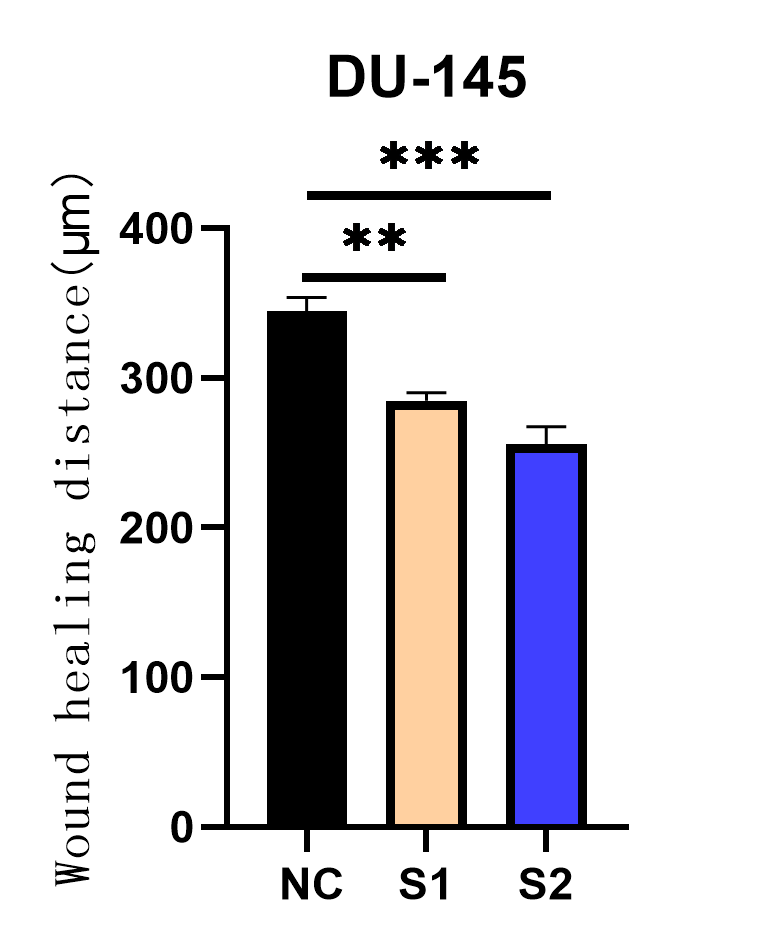

Supplement: S1 File — (ZIP) [file pone.0290753.s001.zip › DU-145 scratch statistical analysis.tif]

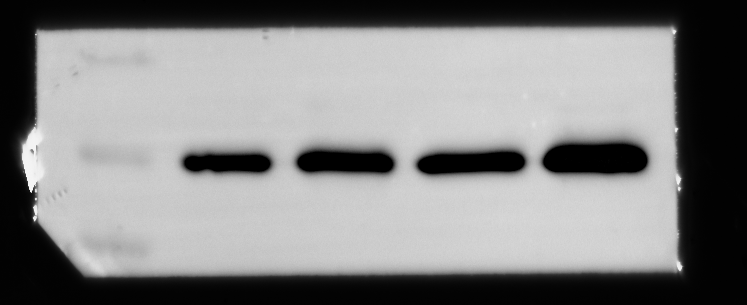

Supplement: S1 File — (ZIP) [file pone.0290753.s001.zip › E-cad.tif]

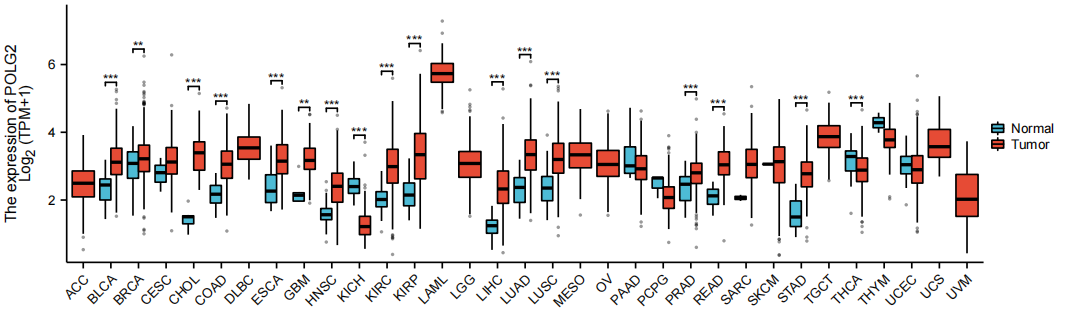

Supplement: S1 File — (ZIP) [file pone.0290753.s001.zip › Fan cancer analysis.tif]

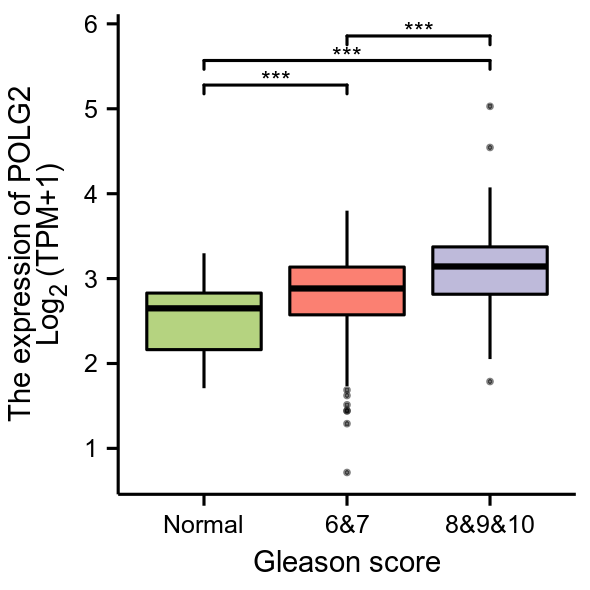

Supplement: S1 File — (ZIP) [file pone.0290753.s001.zip › Gleason score.tiff]

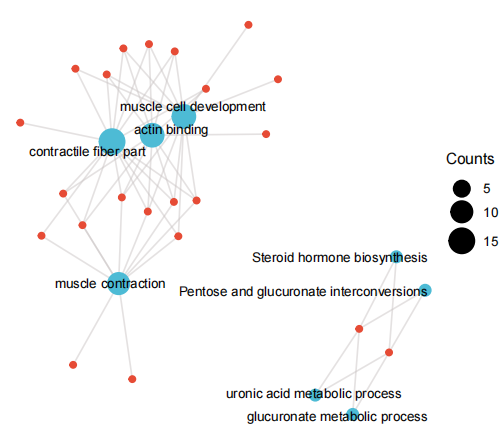

Supplement: S1 File — (ZIP) [file pone.0290753.s001.zip › GO_KEGG visual network.tif]

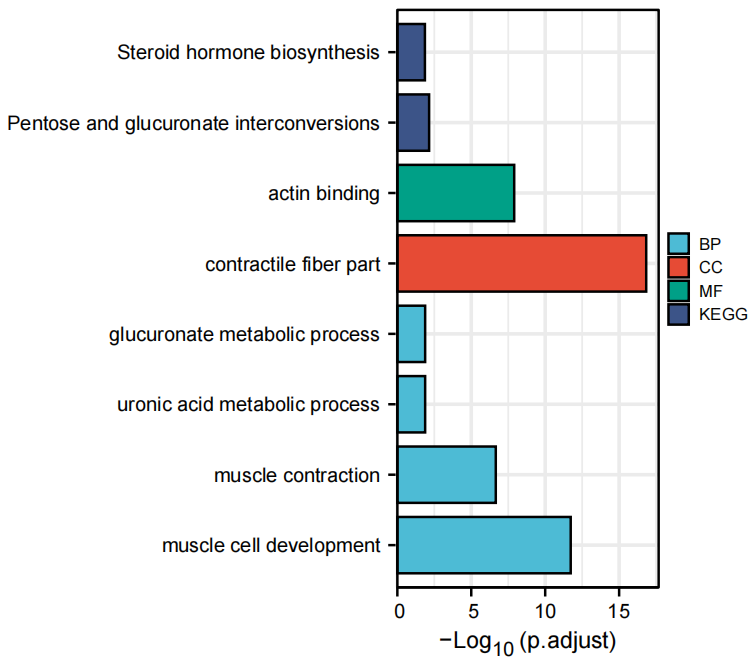

Supplement: S1 File — (ZIP) [file pone.0290753.s001.zip › GO_KEGG visualization.tif]

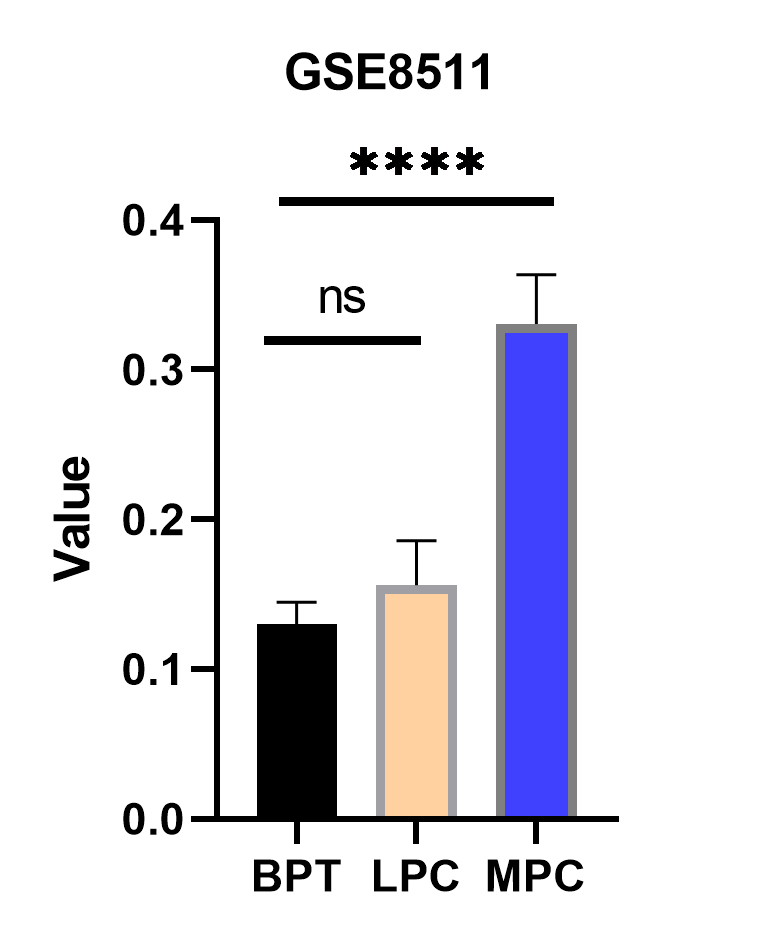

Supplement: S1 File — (ZIP) [file pone.0290753.s001.zip › GSE 8511.tif]

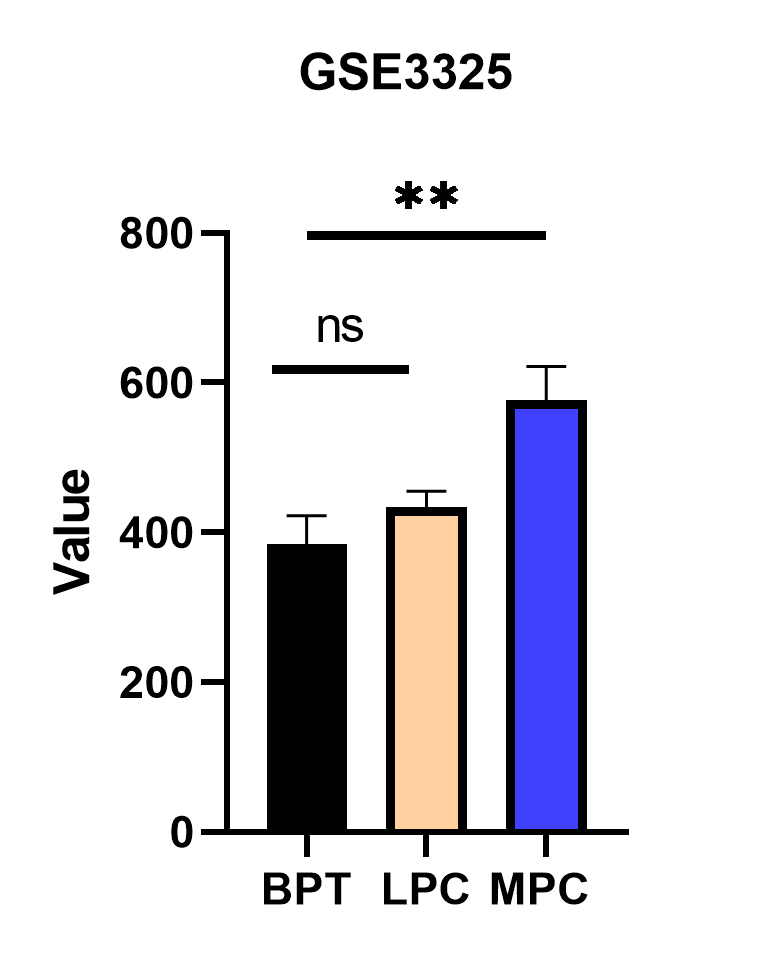

Supplement: S1 File — (ZIP) [file pone.0290753.s001.zip › GSE3325.tif]

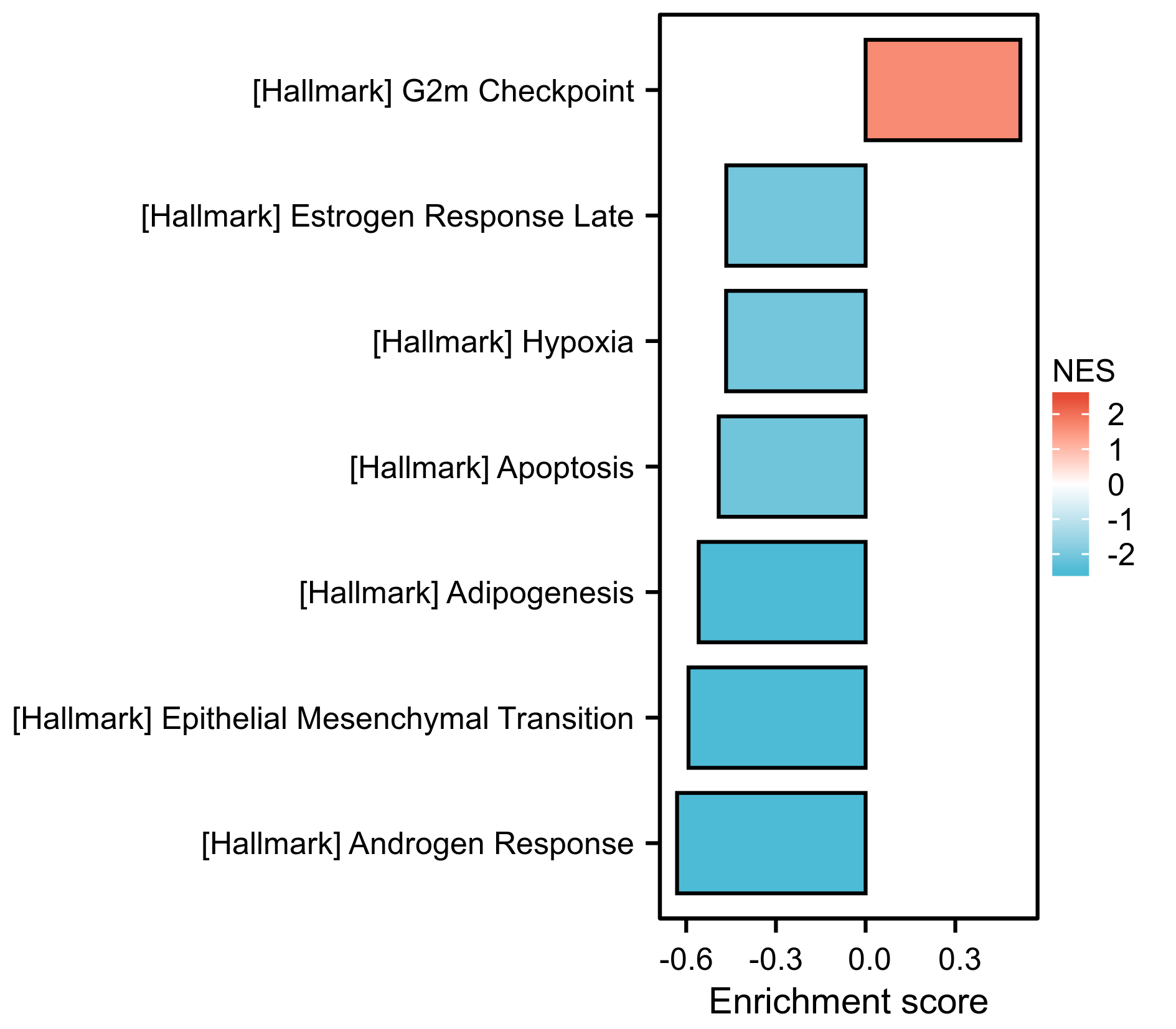

Supplement: S1 File — (ZIP) [file pone.0290753.s001.zip › GSEA histogram.tiff]

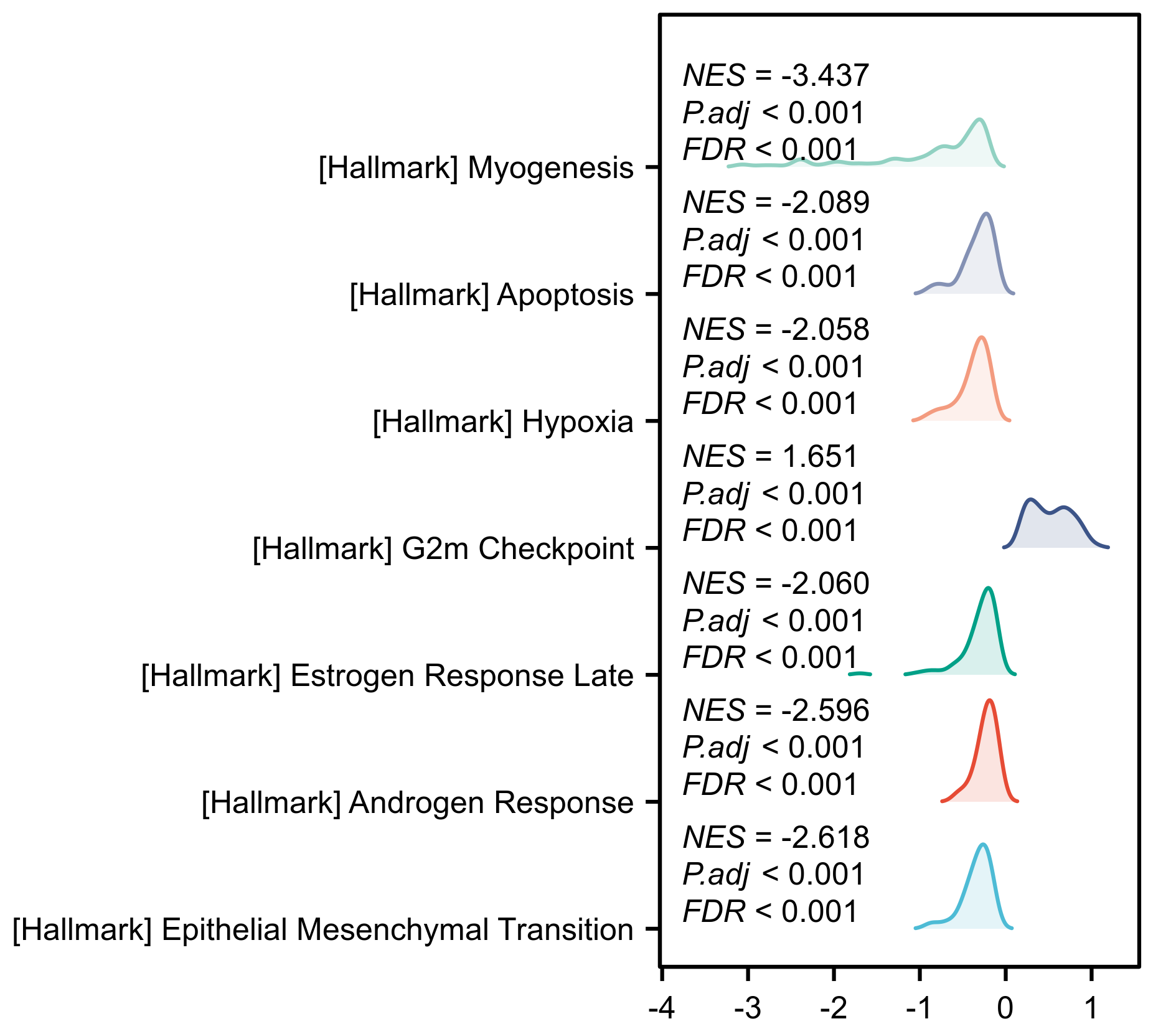

Supplement: S1 File — (ZIP) [file pone.0290753.s001.zip › GSEA Mountain map.tiff]

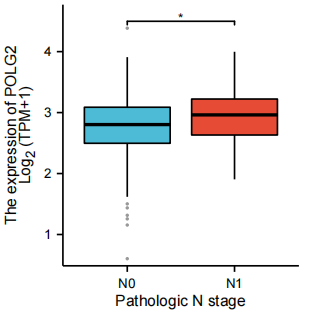

Supplement: S1 File — (ZIP) [file pone.0290753.s001.zip › N stage.tif]

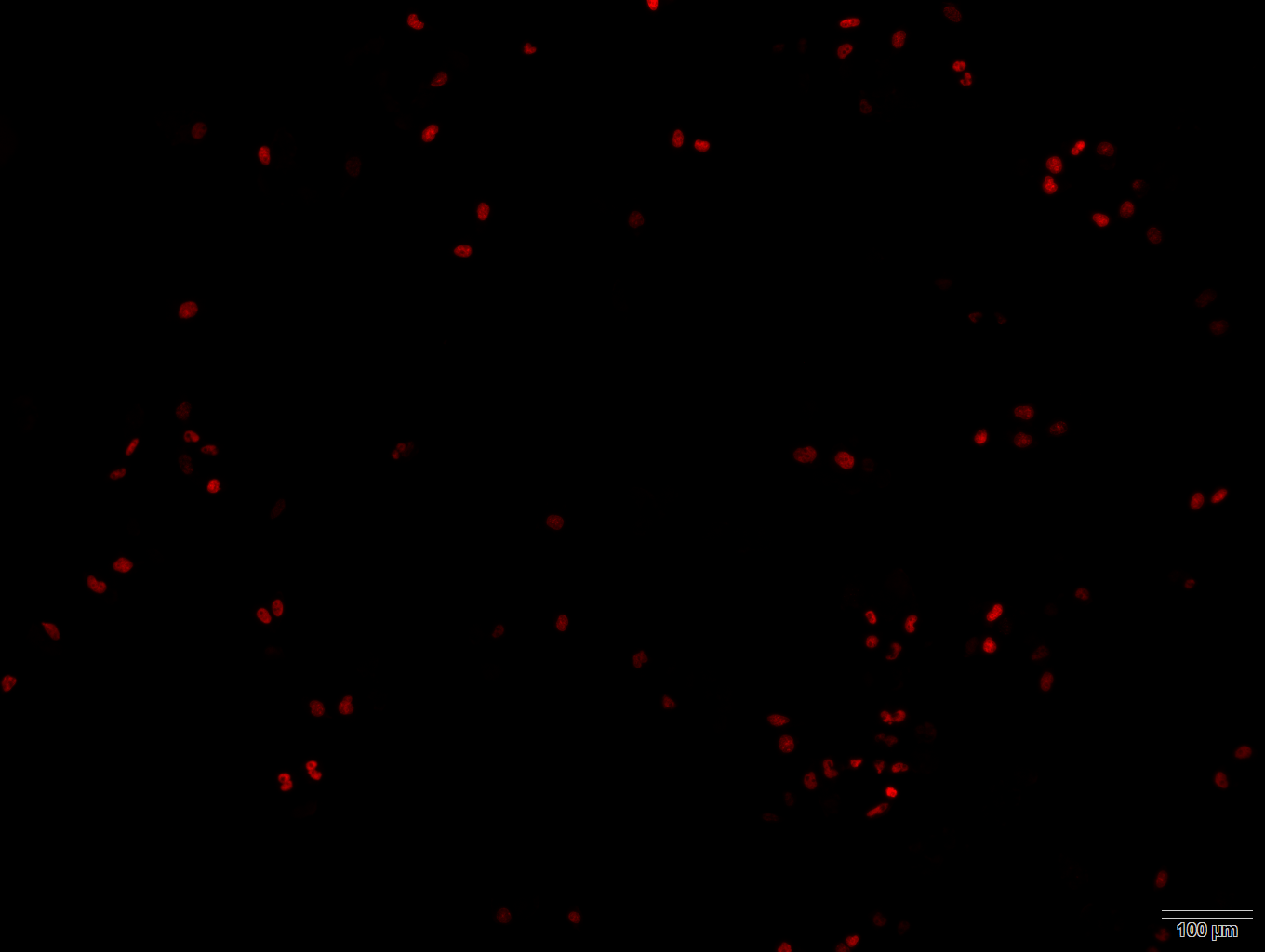

Supplement: S1 File — (ZIP) [file pone.0290753.s001.zip › N-(+)_1.tif]

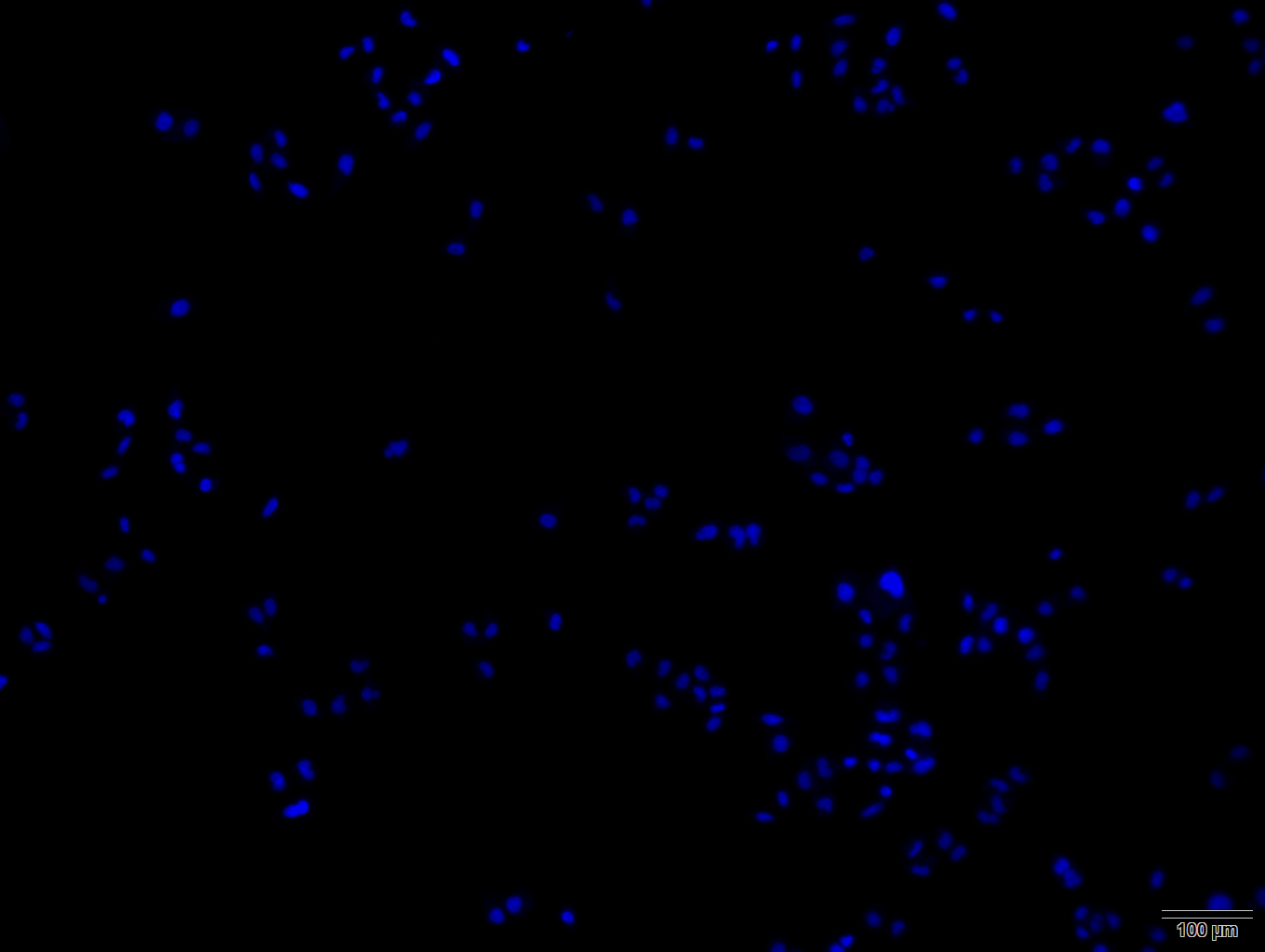

Supplement: S1 File — (ZIP) [file pone.0290753.s001.zip › N-(+)_2.tif]

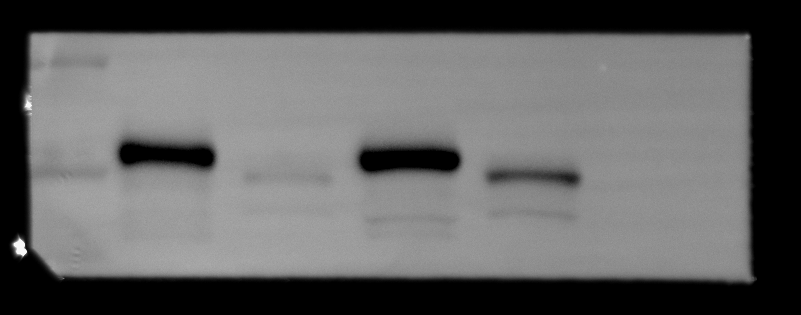

Supplement: S1 File — (ZIP) [file pone.0290753.s001.zip › N-Cad.tif]

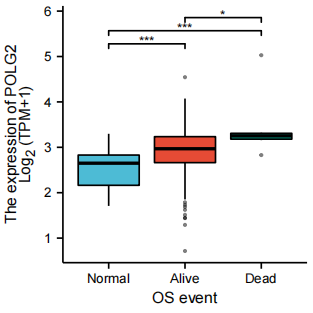

Supplement: S1 File — (ZIP) [file pone.0290753.s001.zip › OS event .tif]

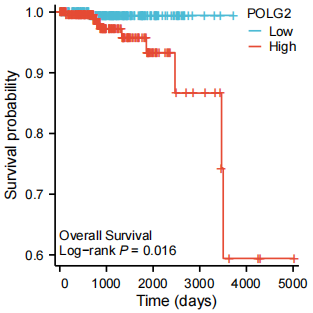

Supplement: S1 File — (ZIP) [file pone.0290753.s001.zip › OS K-M.tif]

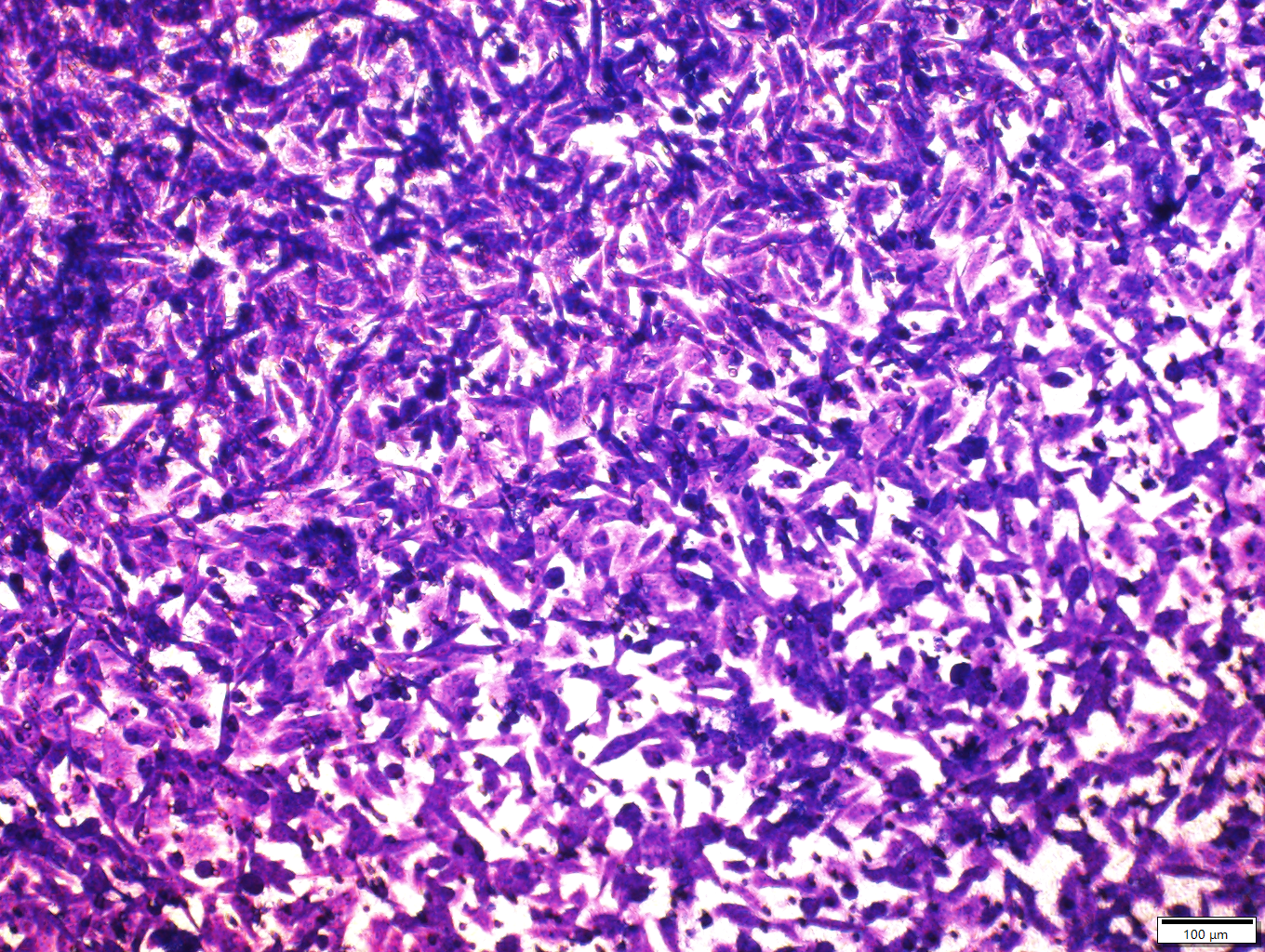

Supplement: S1 File — (ZIP) [file pone.0290753.s001.zip › pc nc invasion.tif]

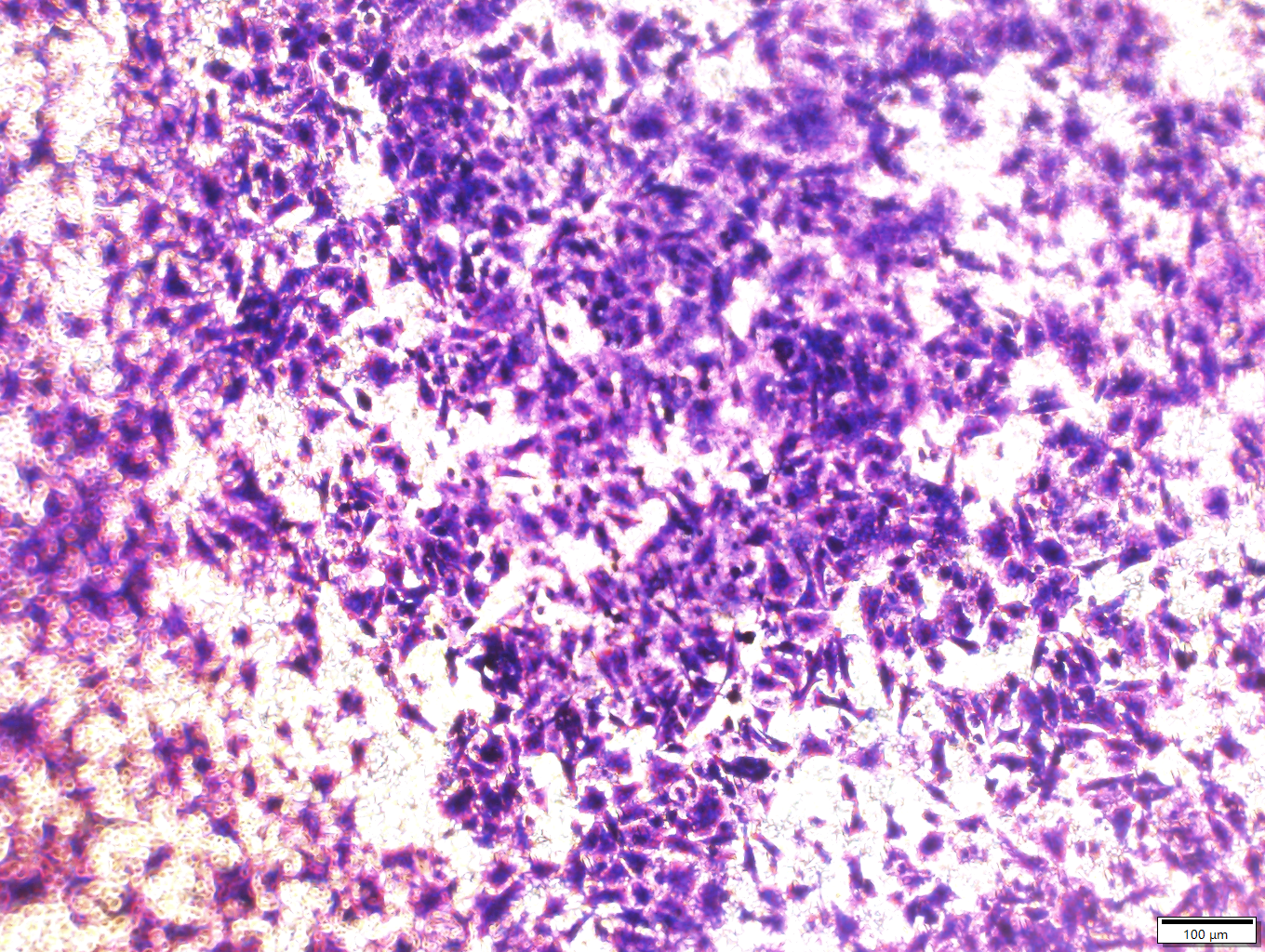

Supplement: S1 File — (ZIP) [file pone.0290753.s001.zip › pc shPOLG2 invasion.tif]

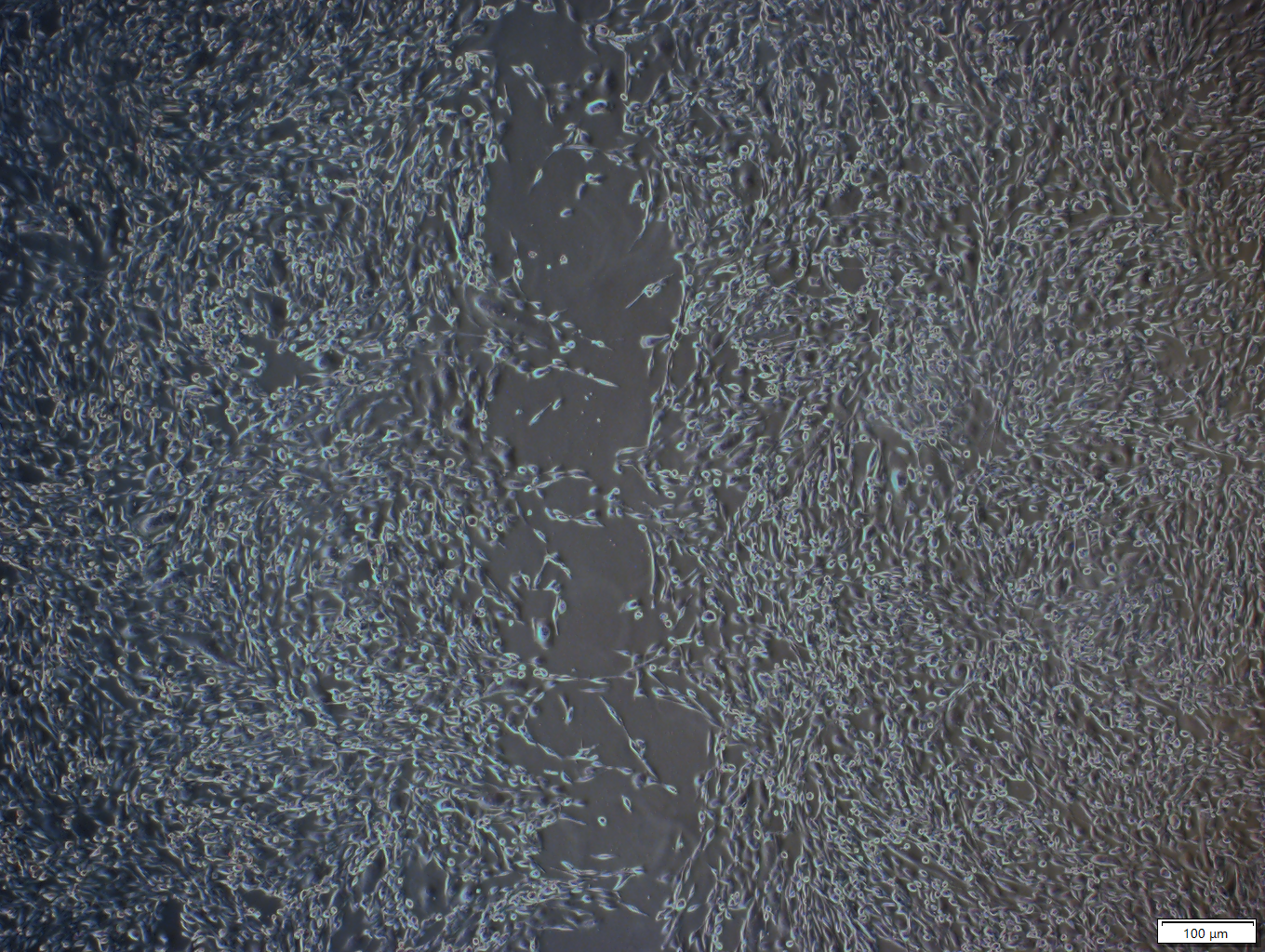

Supplement: S1 File — (ZIP) [file pone.0290753.s001.zip › PC-3 NC 48h.tif]

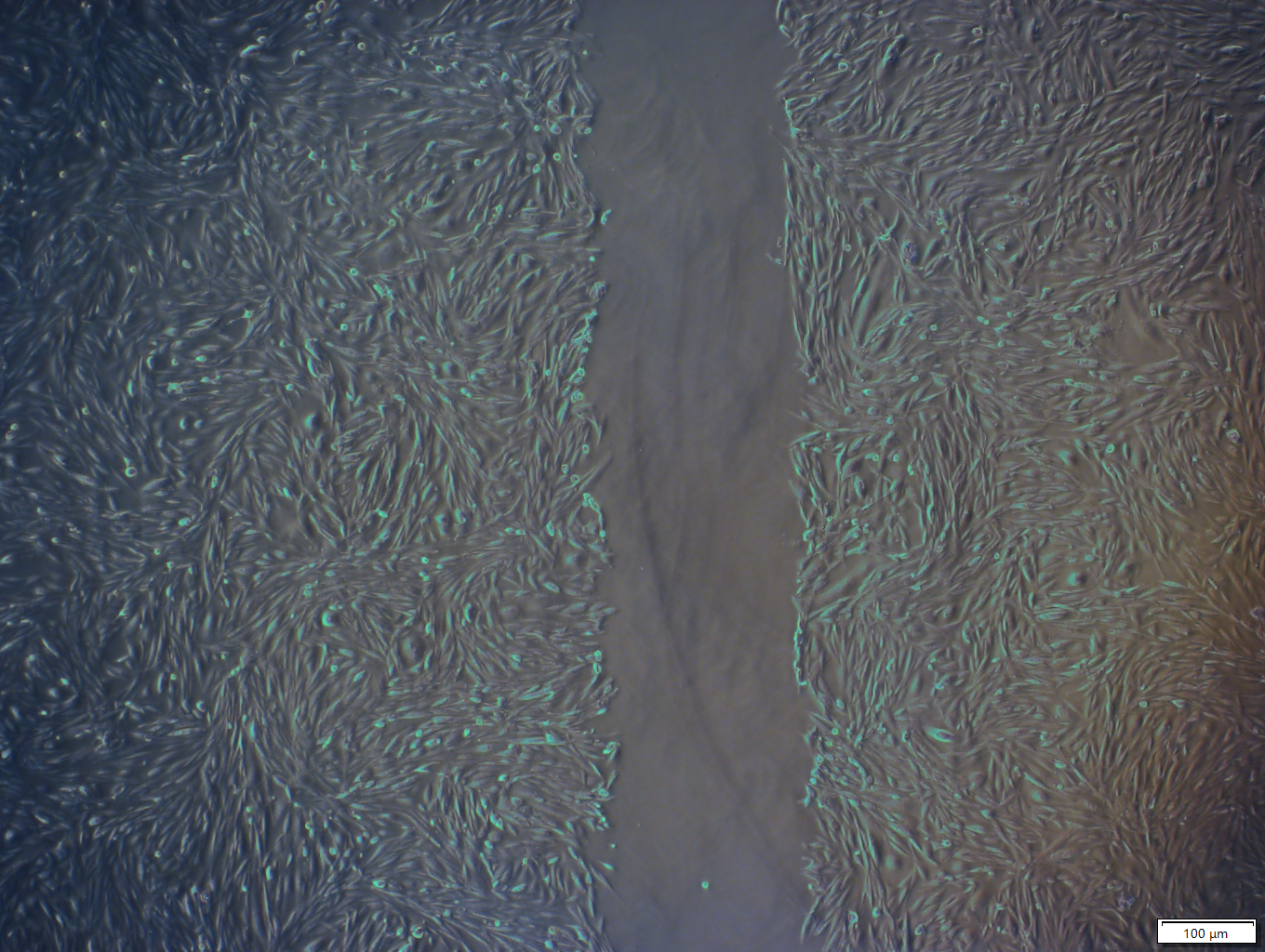

Supplement: S1 File — (ZIP) [file pone.0290753.s001.zip › pc-3 NC.tif]

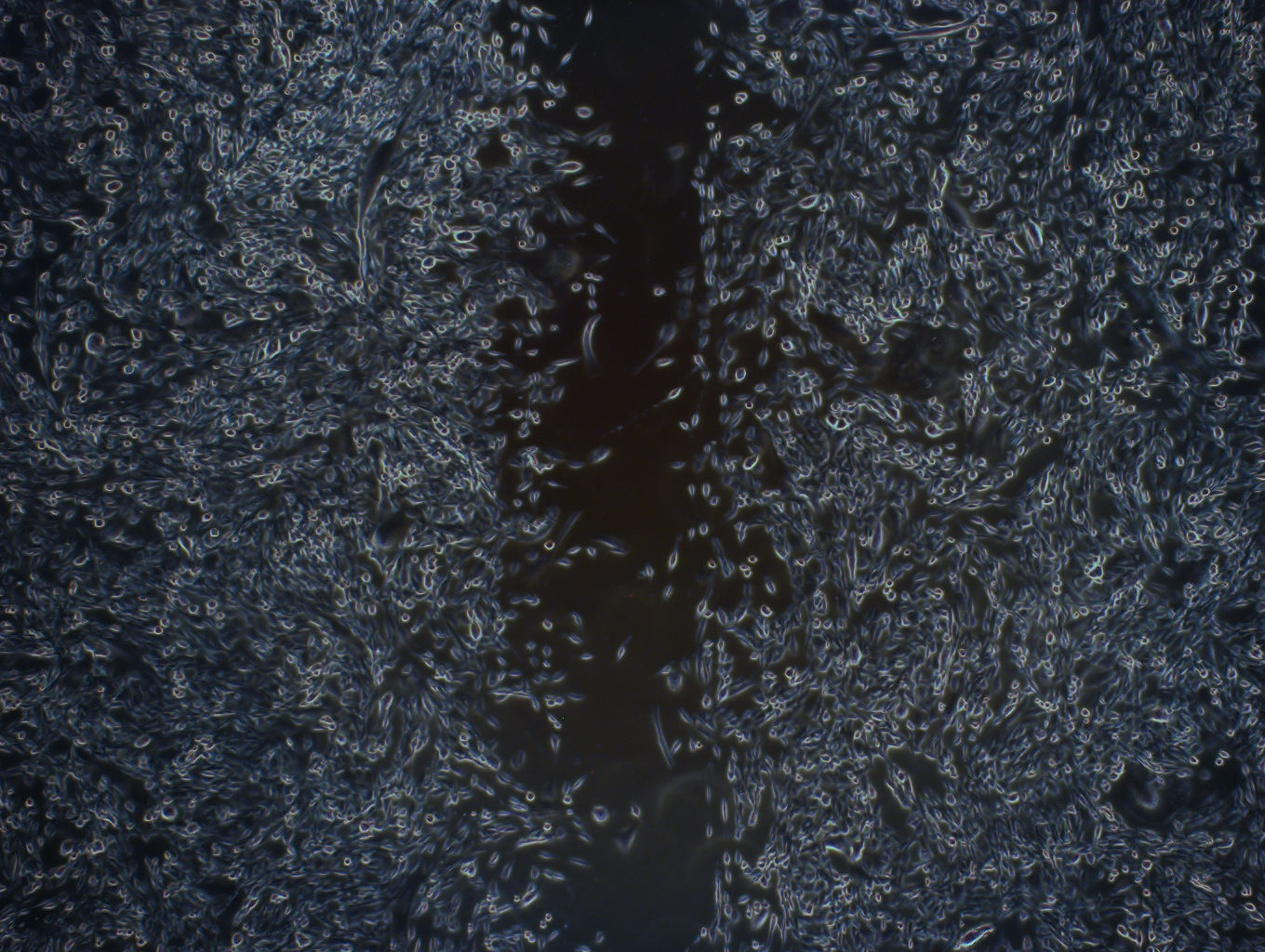

Supplement: S1 File — (ZIP) [file pone.0290753.s001.zip › pc-3 shPOLG2 48h.tif]

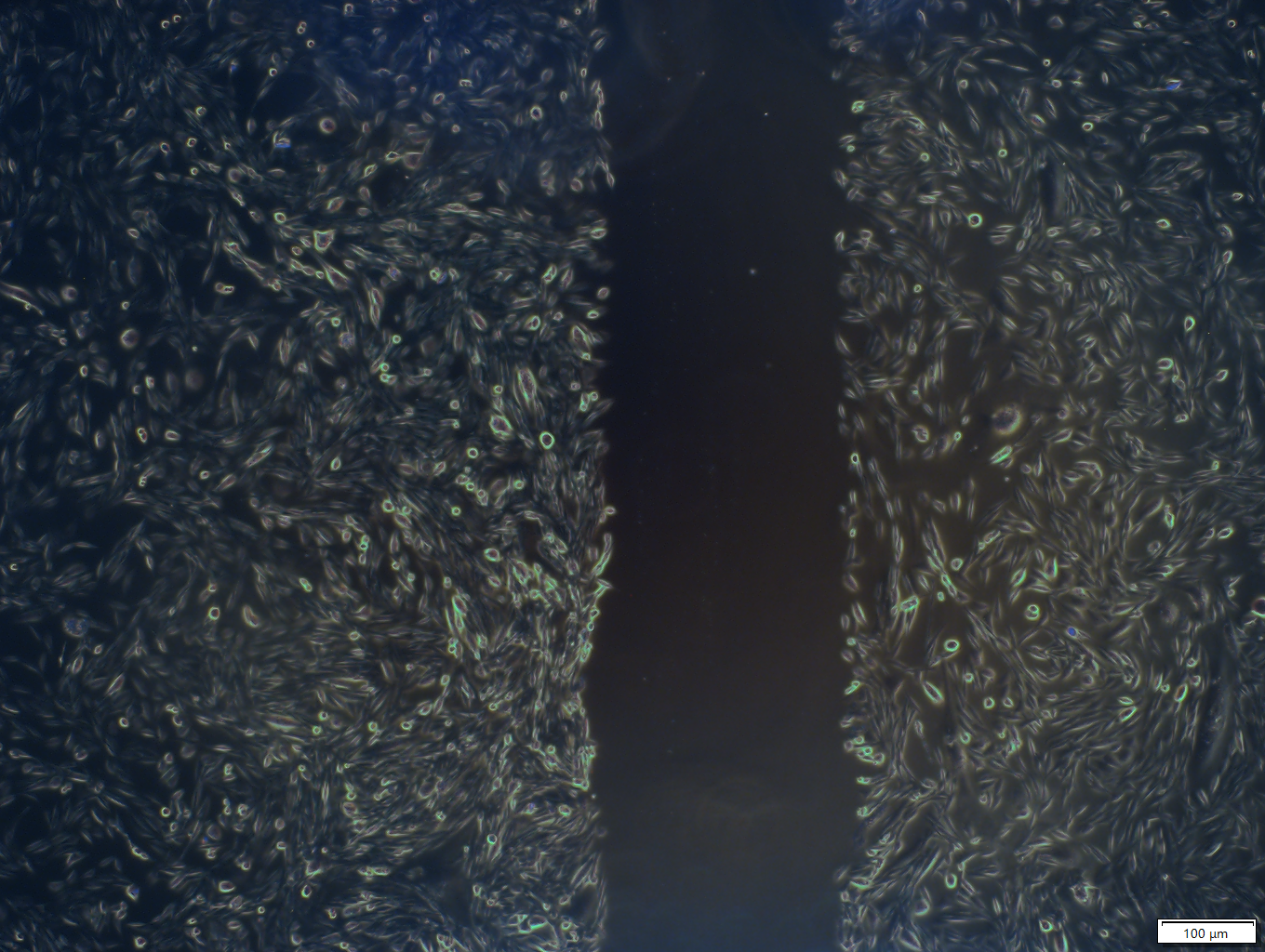

Supplement: S1 File — (ZIP) [file pone.0290753.s001.zip › pc-3 shPOLG2.tif]

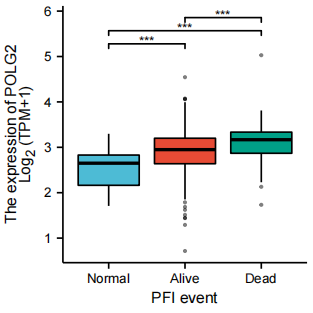

Supplement: S1 File — (ZIP) [file pone.0290753.s001.zip › PFI .tif]

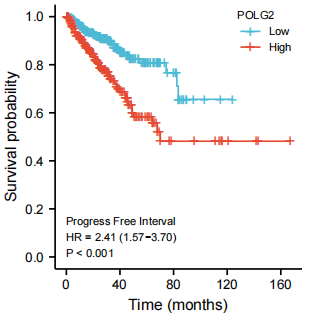

Supplement: S1 File — (ZIP) [file pone.0290753.s001.zip › PFI K-M.tif]

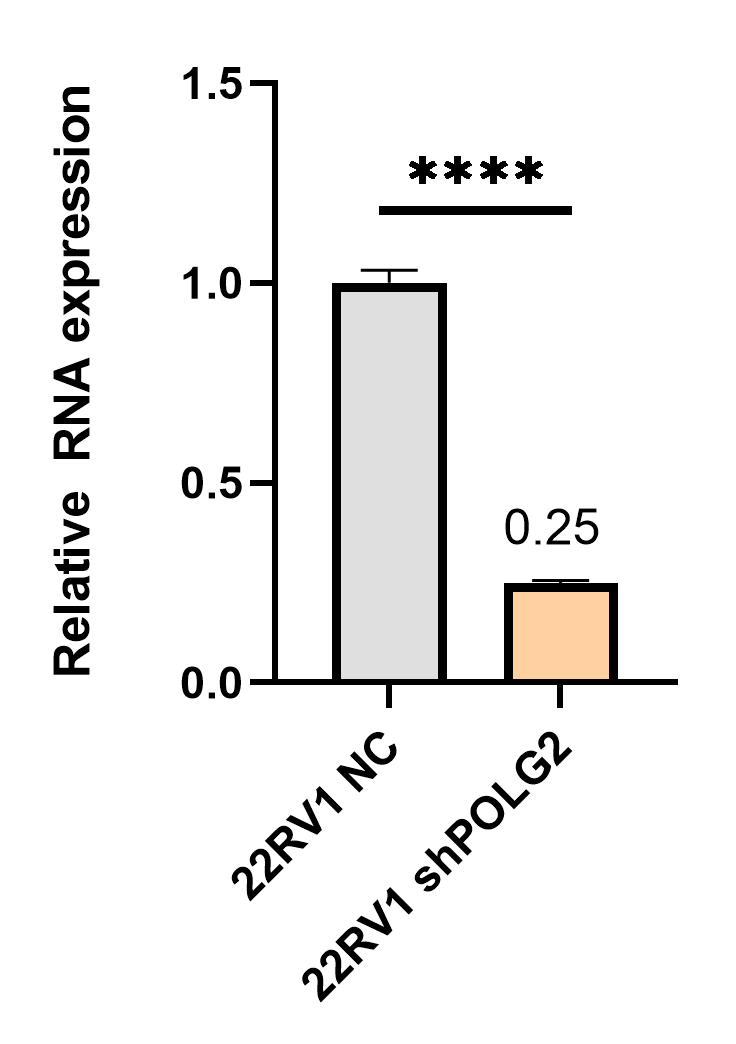

Supplement: S1 File — (ZIP) [file pone.0290753.s001.zip › POLG2 Knockdown efficiency in 22RV1stable cell line .tif]

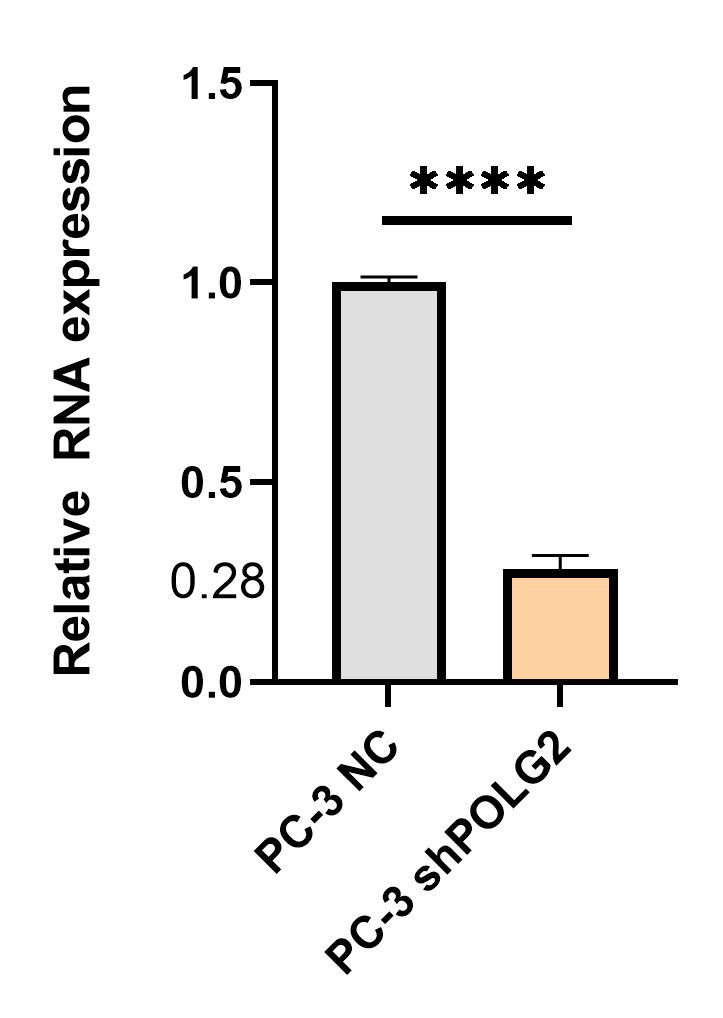

Supplement: S1 File — (ZIP) [file pone.0290753.s001.zip › POLG2 Knockdown efficiency in PC-3 stable cell line .tif]

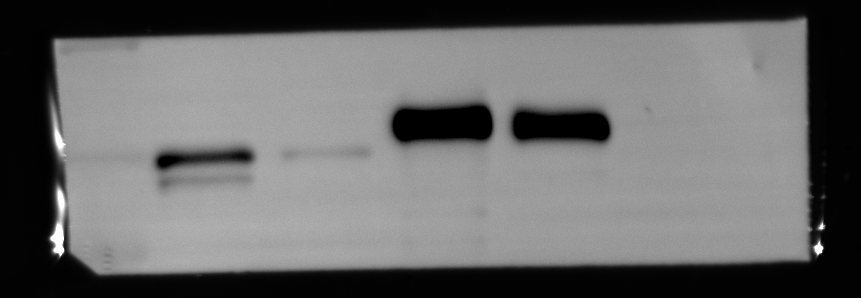

Supplement: S1 File — (ZIP) [file pone.0290753.s001.zip › POLG2.tif]

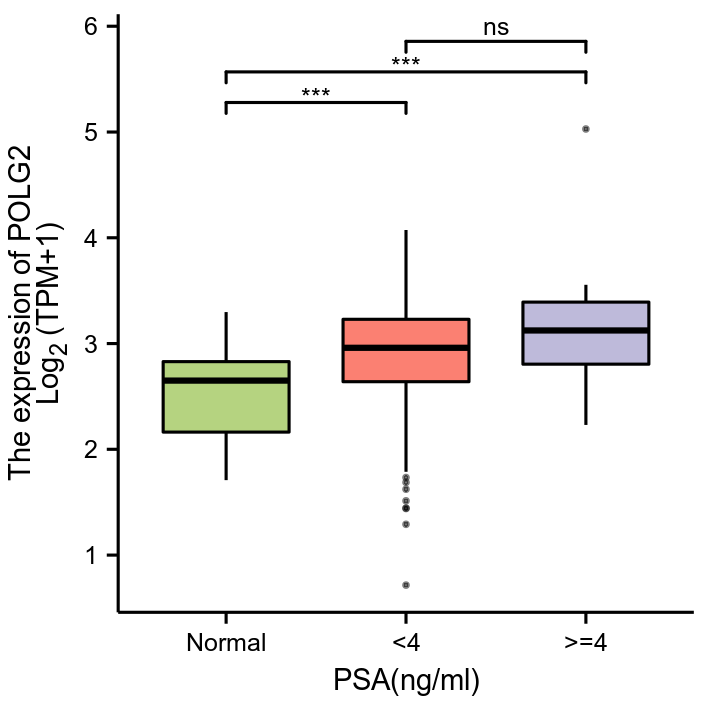

Supplement: S1 File — (ZIP) [file pone.0290753.s001.zip › PSA.tiff]

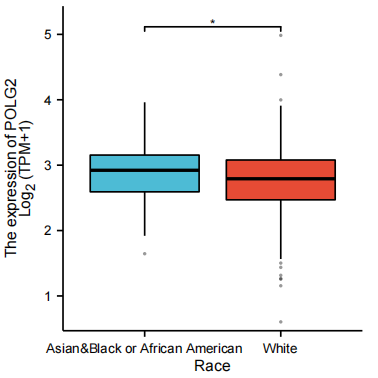

Supplement: S1 File — (ZIP) [file pone.0290753.s001.zip › RACE.tif]

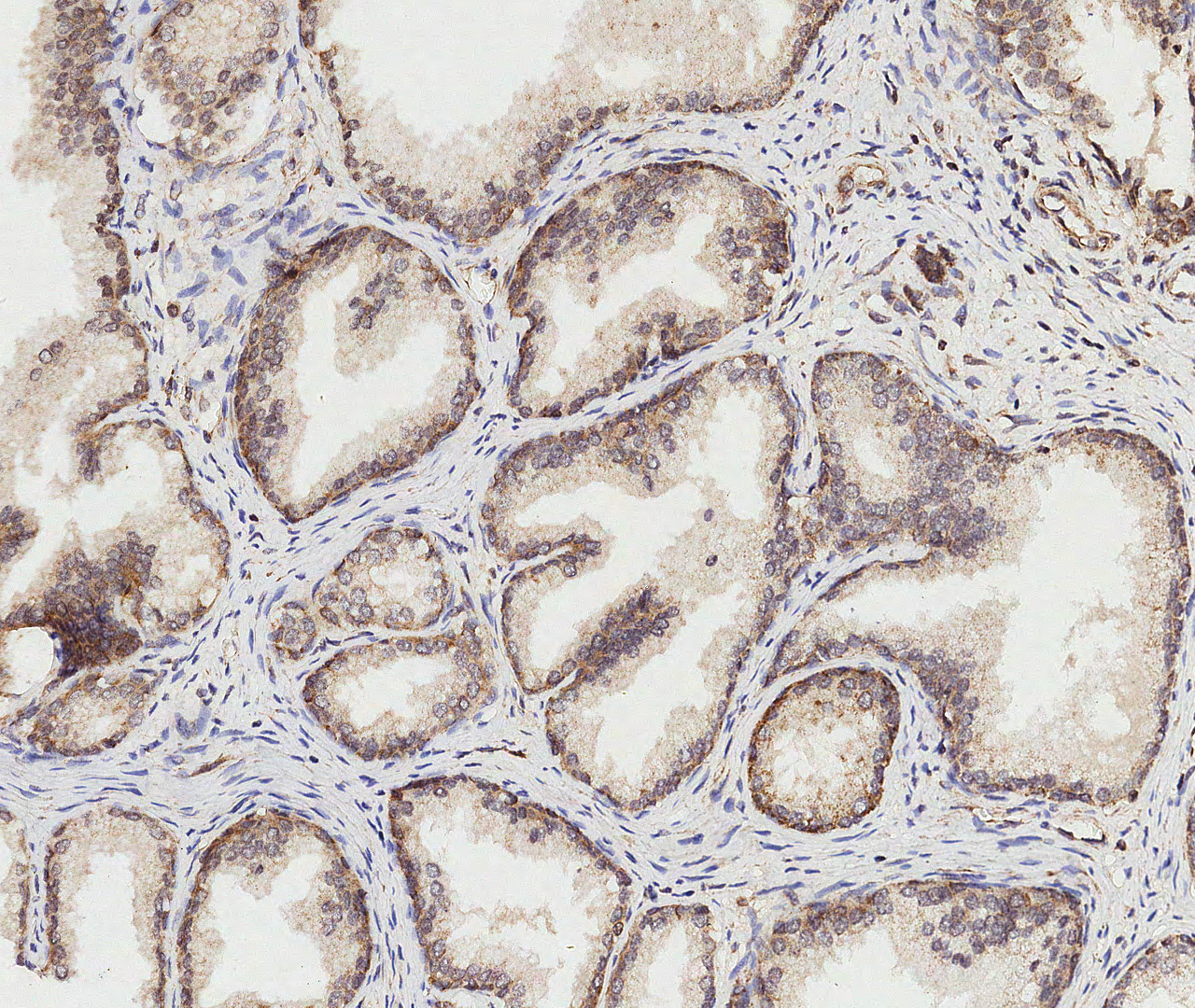

Supplement: S1 File — (ZIP) [file pone.0290753.s001.zip › renamed_12943.tif]

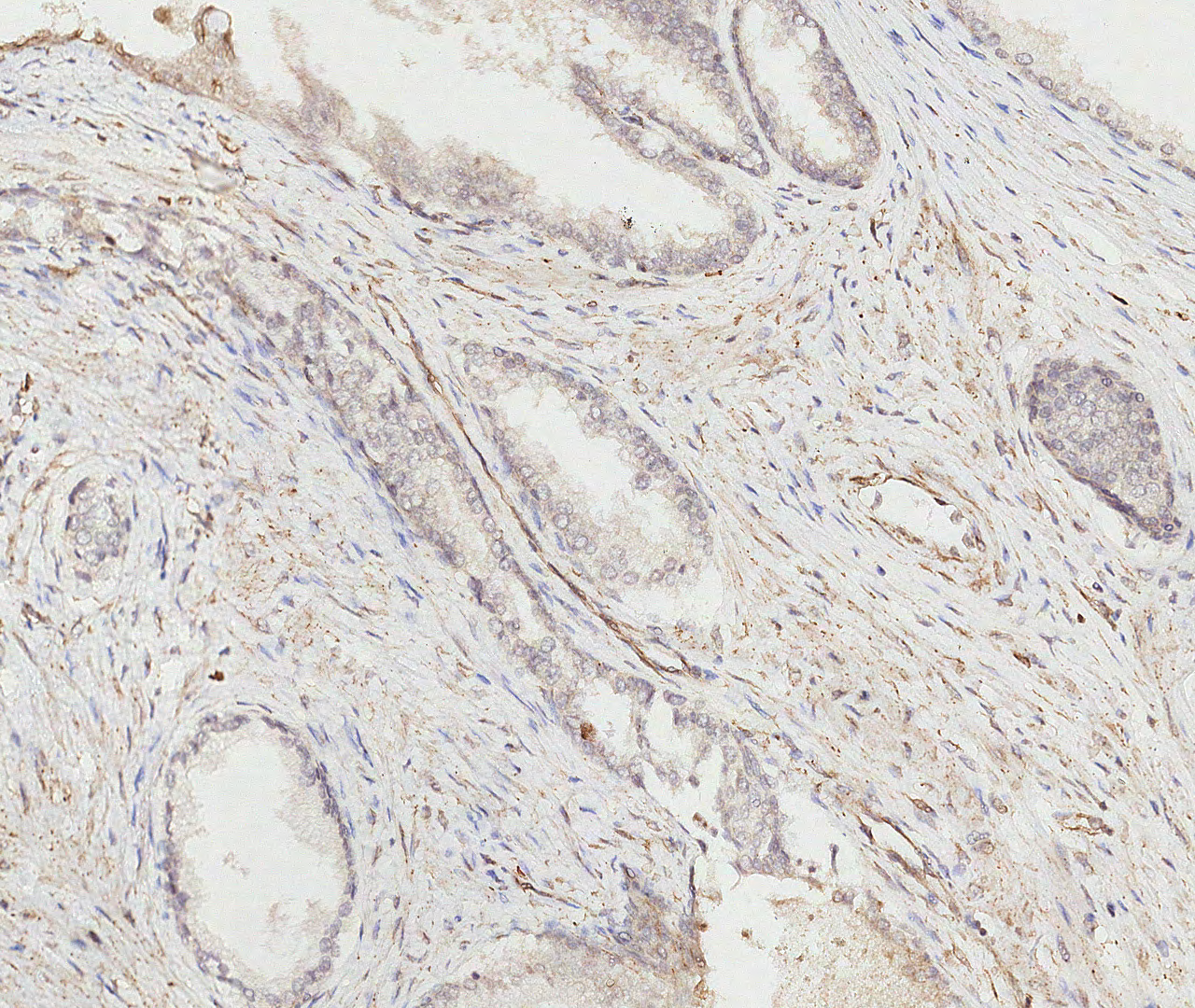

Supplement: S1 File — (ZIP) [file pone.0290753.s001.zip › renamed_13cdc.tif]

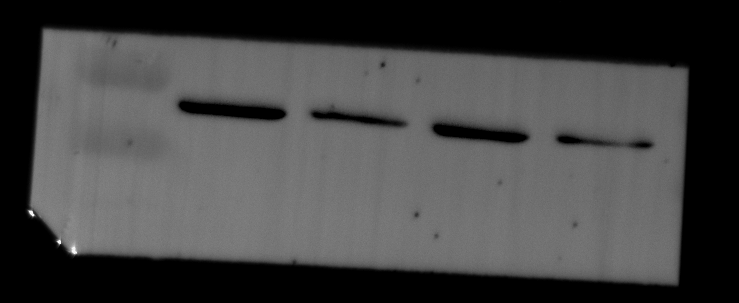

Supplement: S1 File — (ZIP) [file pone.0290753.s001.zip › renamed_16675.tif]

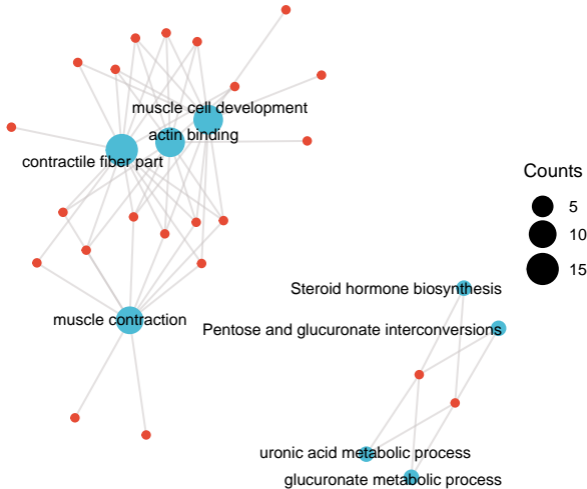

Supplement: S1 File — (ZIP) [file pone.0290753.s001.zip › renamed_20a57.pdf]

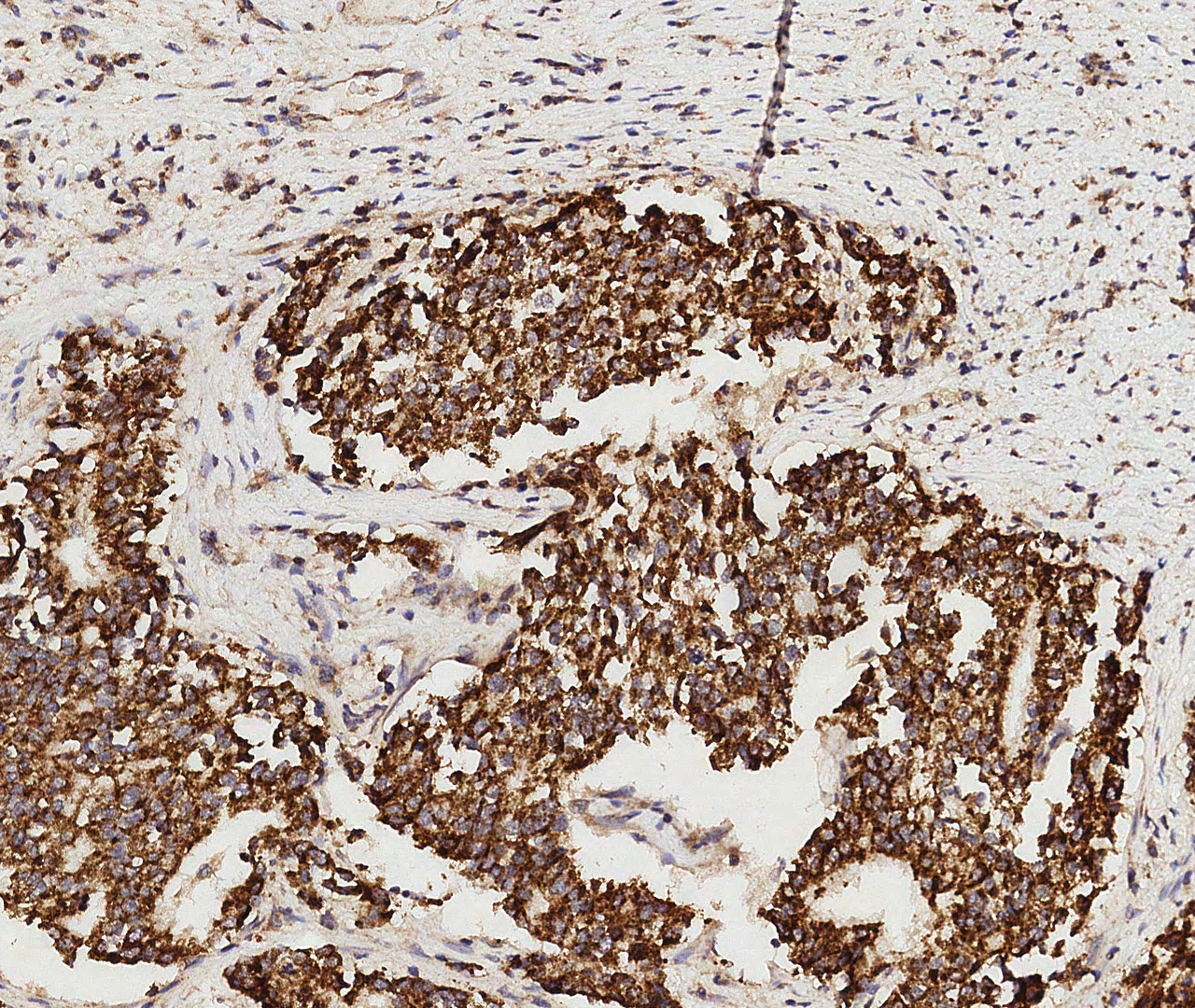

Supplement: S1 File — (ZIP) [file pone.0290753.s001.zip › renamed_294b3.tif]

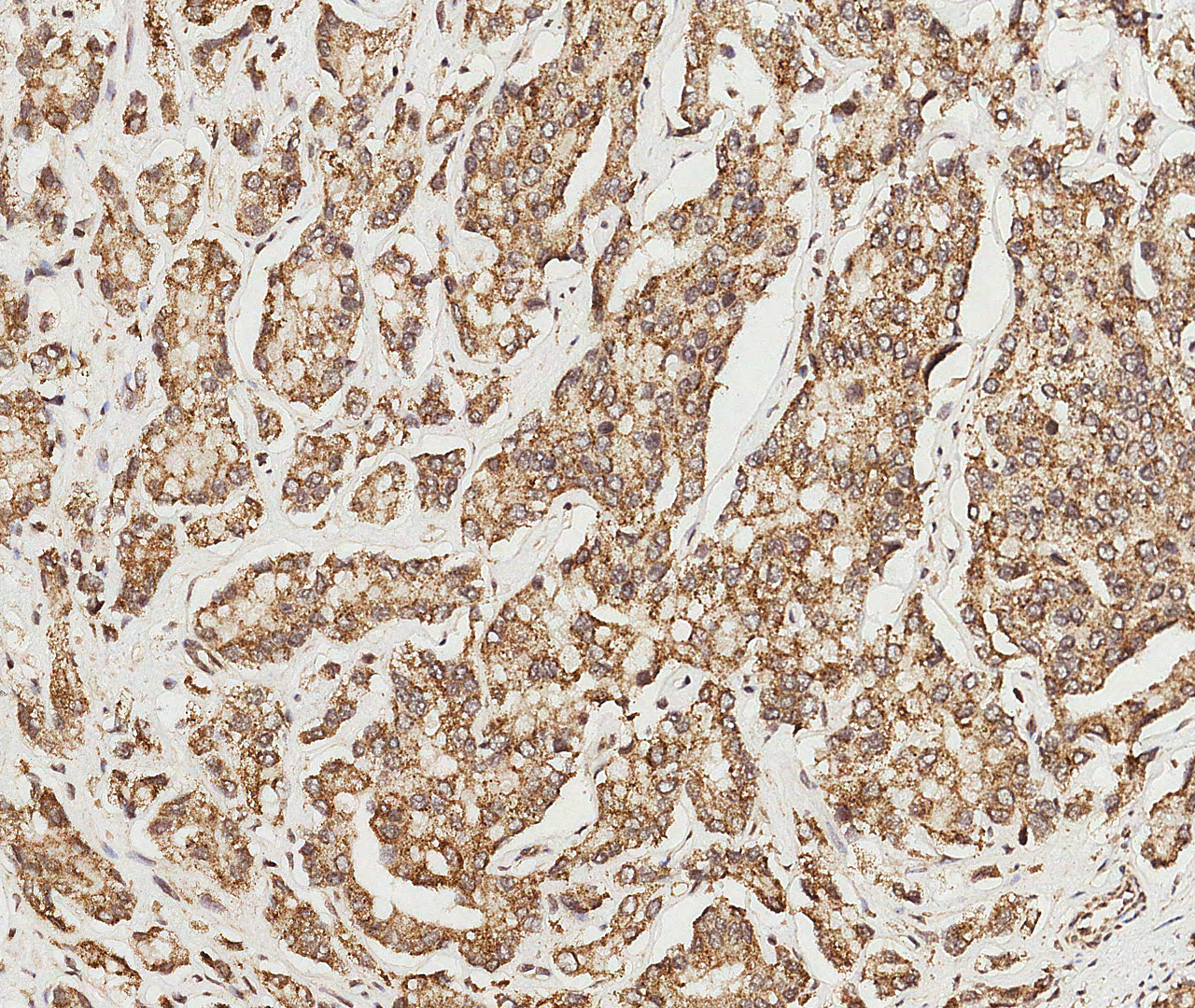

Supplement: S1 File — (ZIP) [file pone.0290753.s001.zip › renamed_663ca.tif]

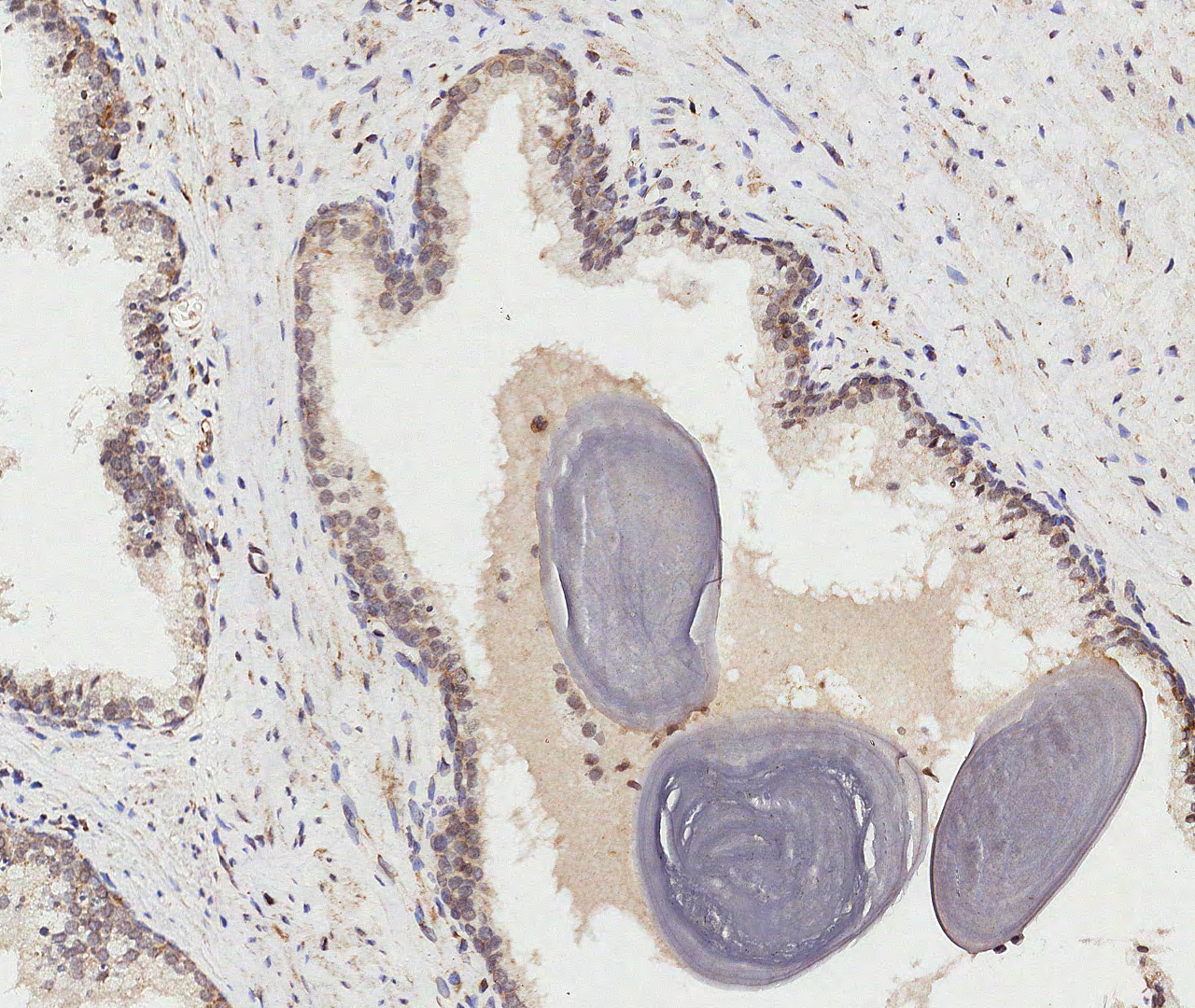

Supplement: S1 File — (ZIP) [file pone.0290753.s001.zip › renamed_7a525.tif]

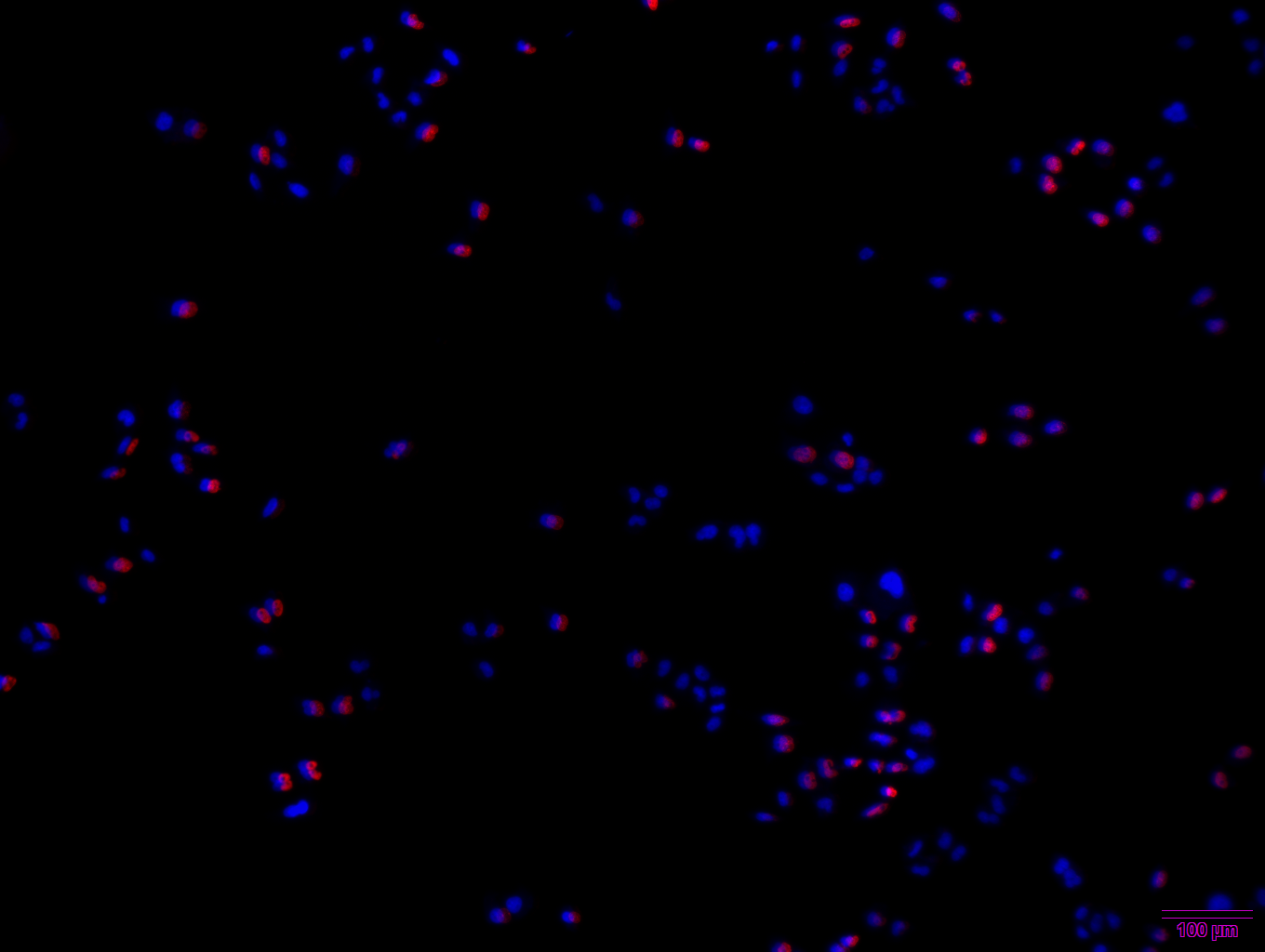

Supplement: S1 File — (ZIP) [file pone.0290753.s001.zip › renamed_97aff.tif]

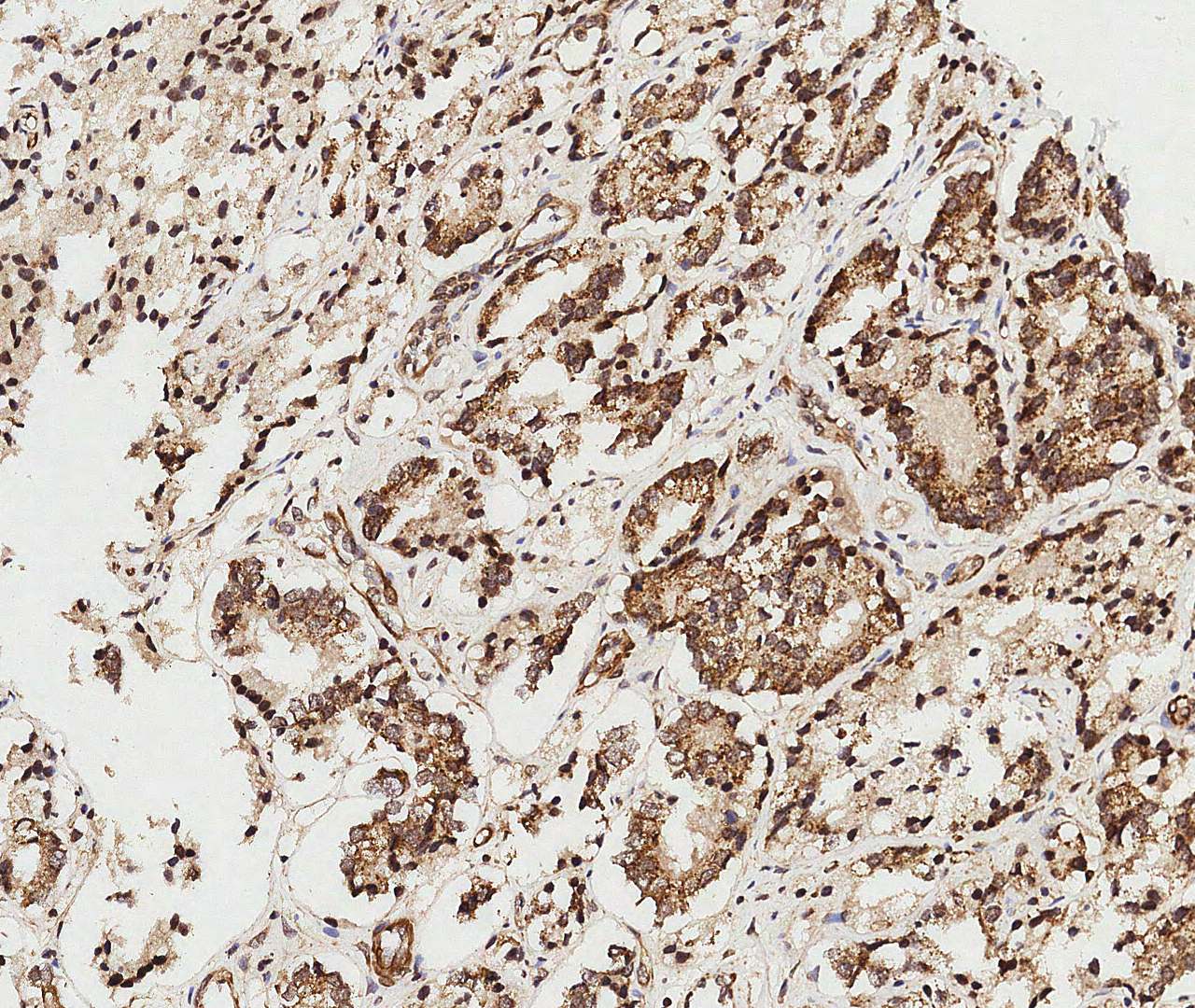

Supplement: S1 File — (ZIP) [file pone.0290753.s001.zip › renamed_a57d8.tif]

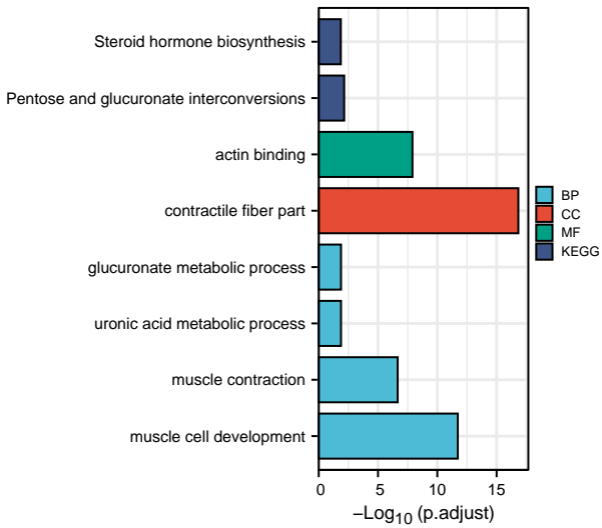

Supplement: S1 File — (ZIP) [file pone.0290753.s001.zip › renamed_d9678.pdf]

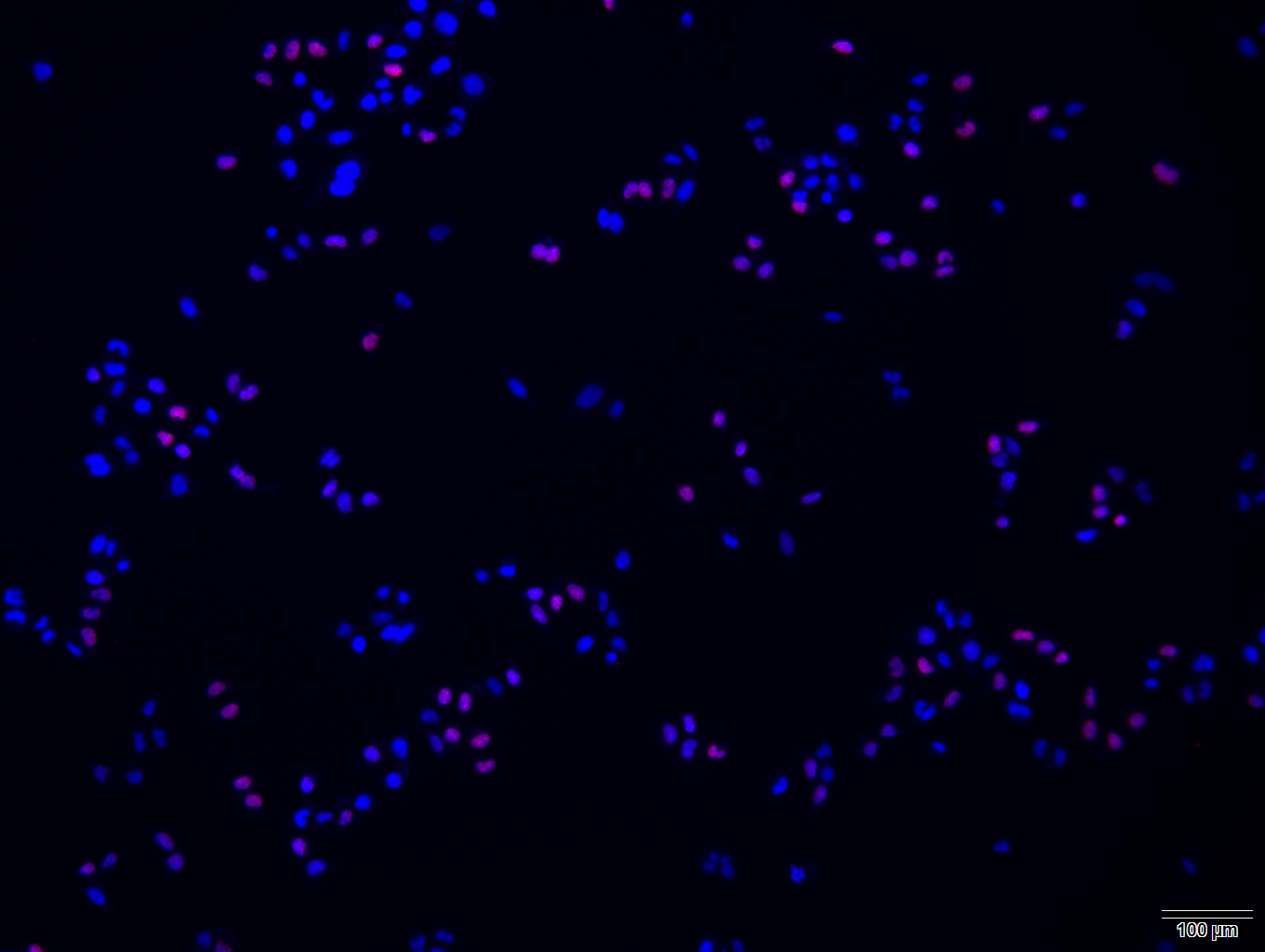

Supplement: S1 File — (ZIP) [file pone.0290753.s001.zip › s1 6 7merge .tif]

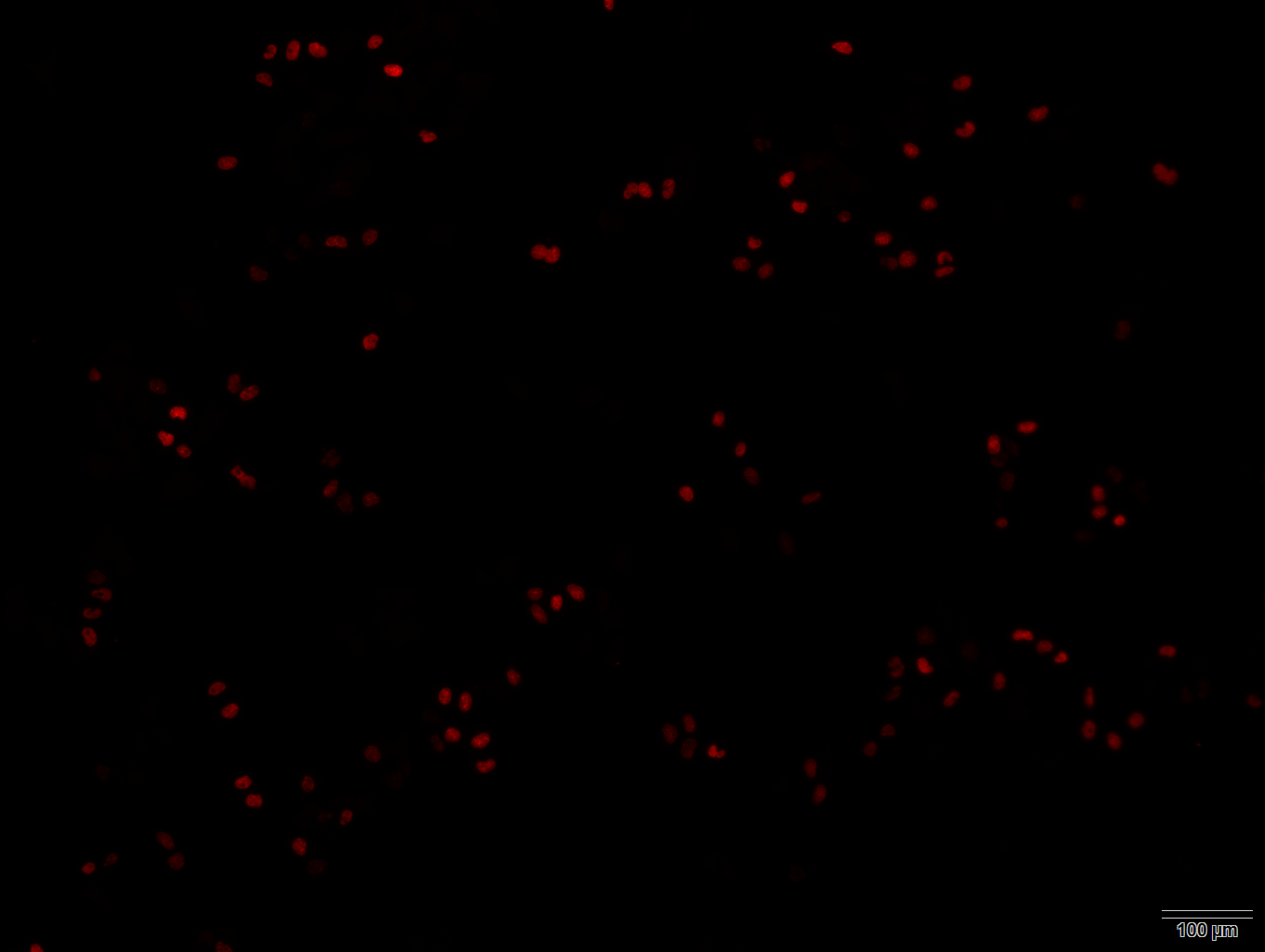

Supplement: S1 File — (ZIP) [file pone.0290753.s001.zip › s1 6.tif]

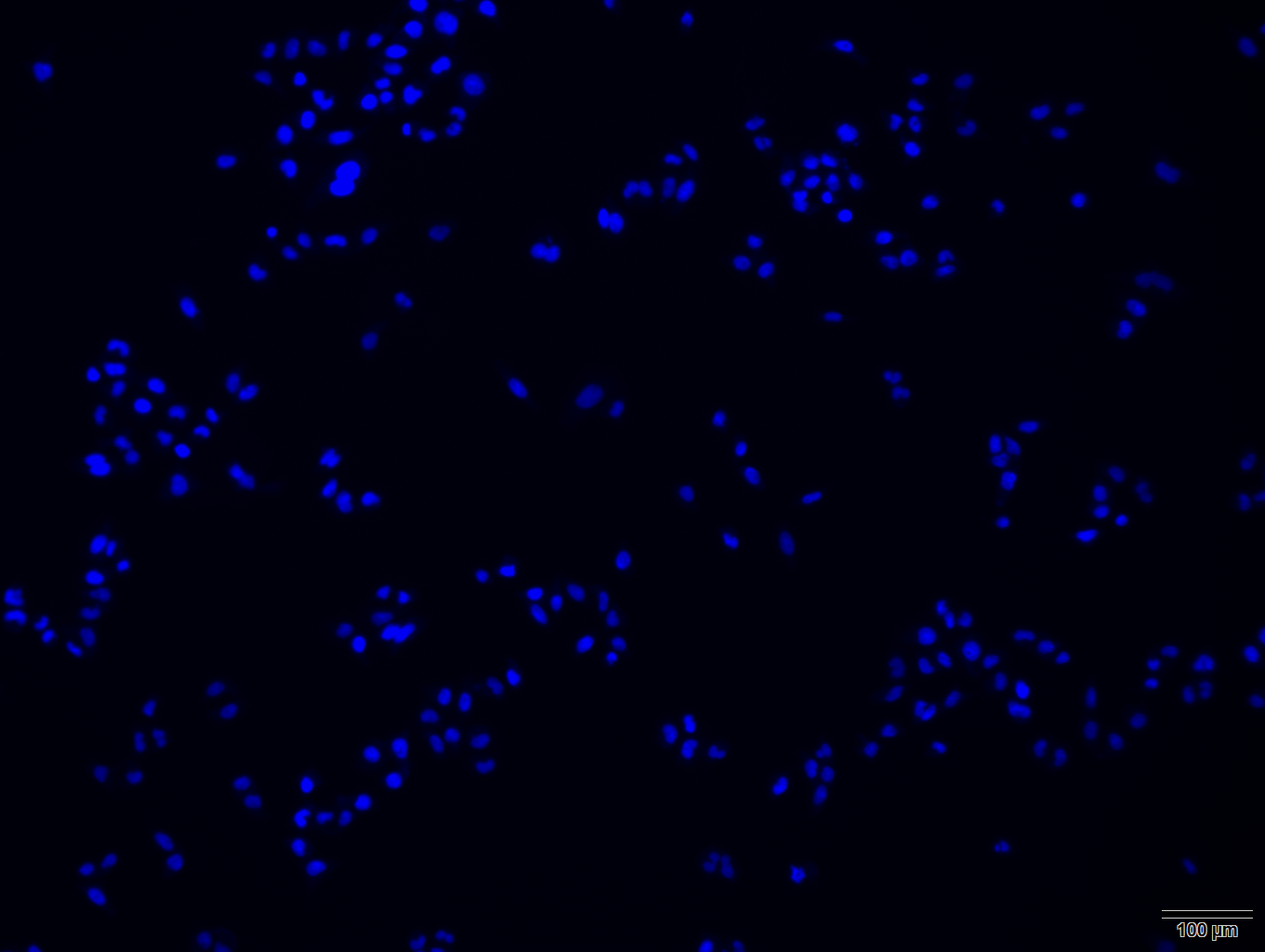

Supplement: S1 File — (ZIP) [file pone.0290753.s001.zip › s1 7.tif]

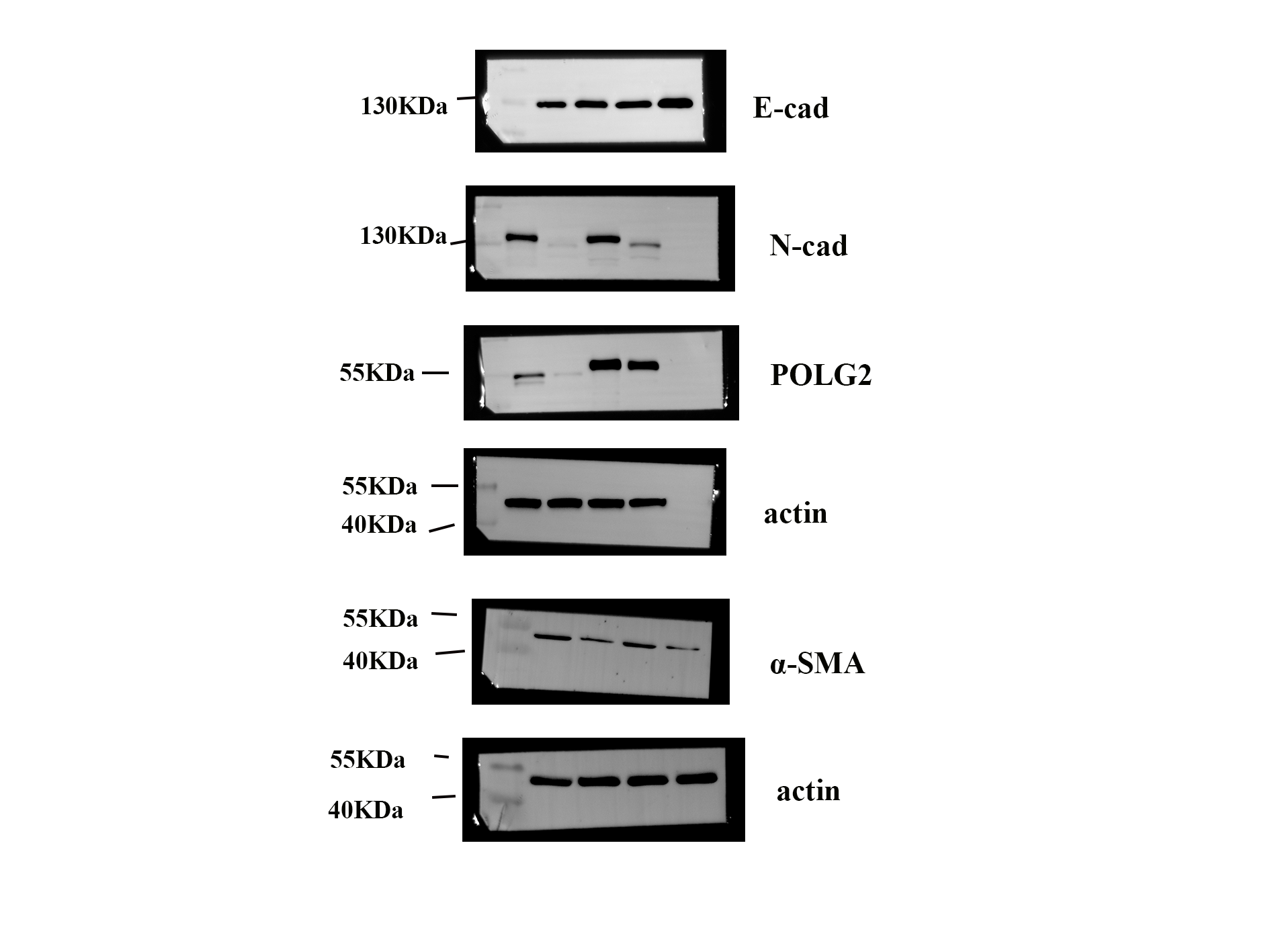

Supplement: S1 File — (ZIP) [file pone.0290753.s001.zip › S1_raw_images.tif]

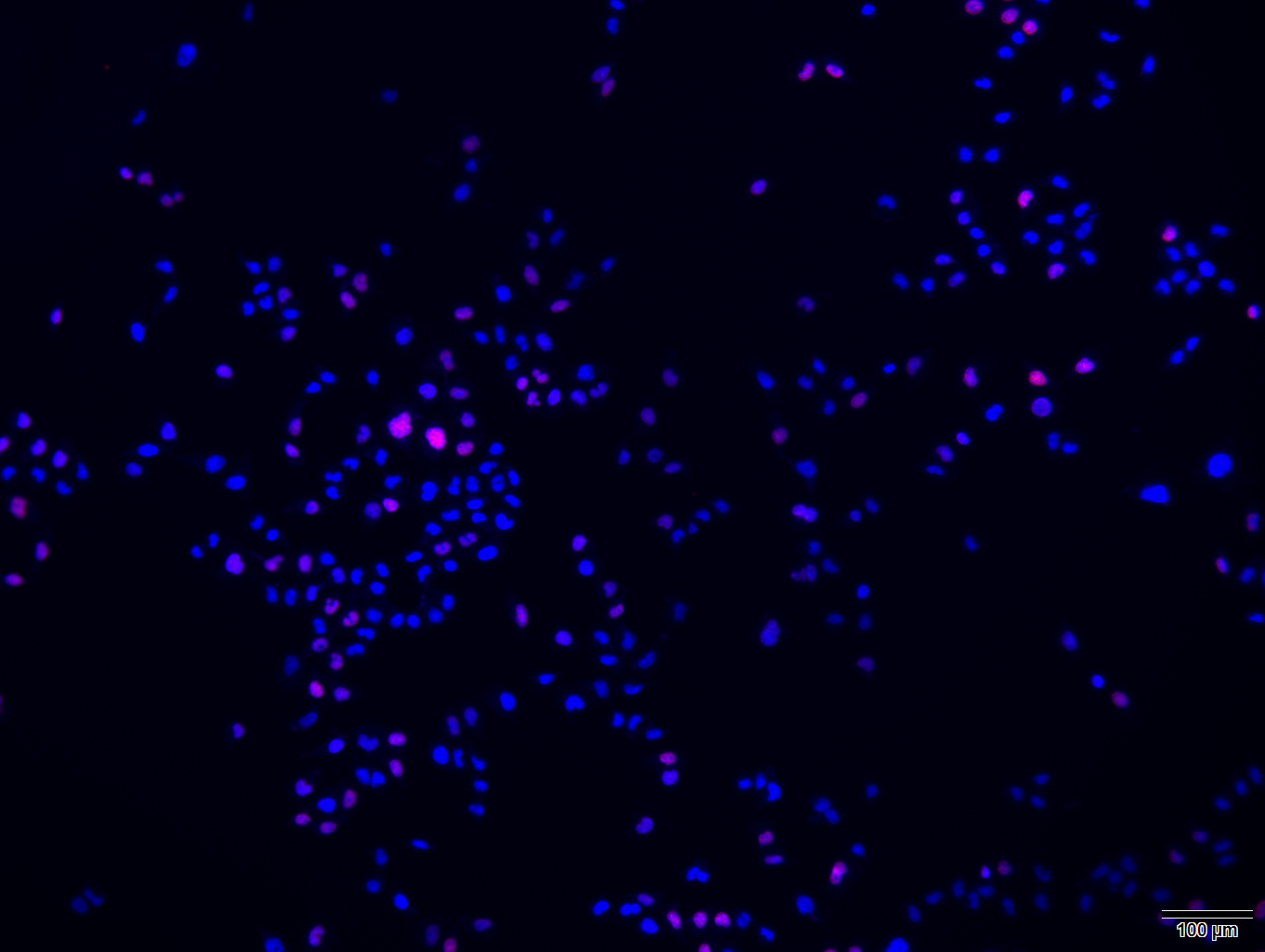

Supplement: S1 File — (ZIP) [file pone.0290753.s001.zip › S2 26 27merge.tif]

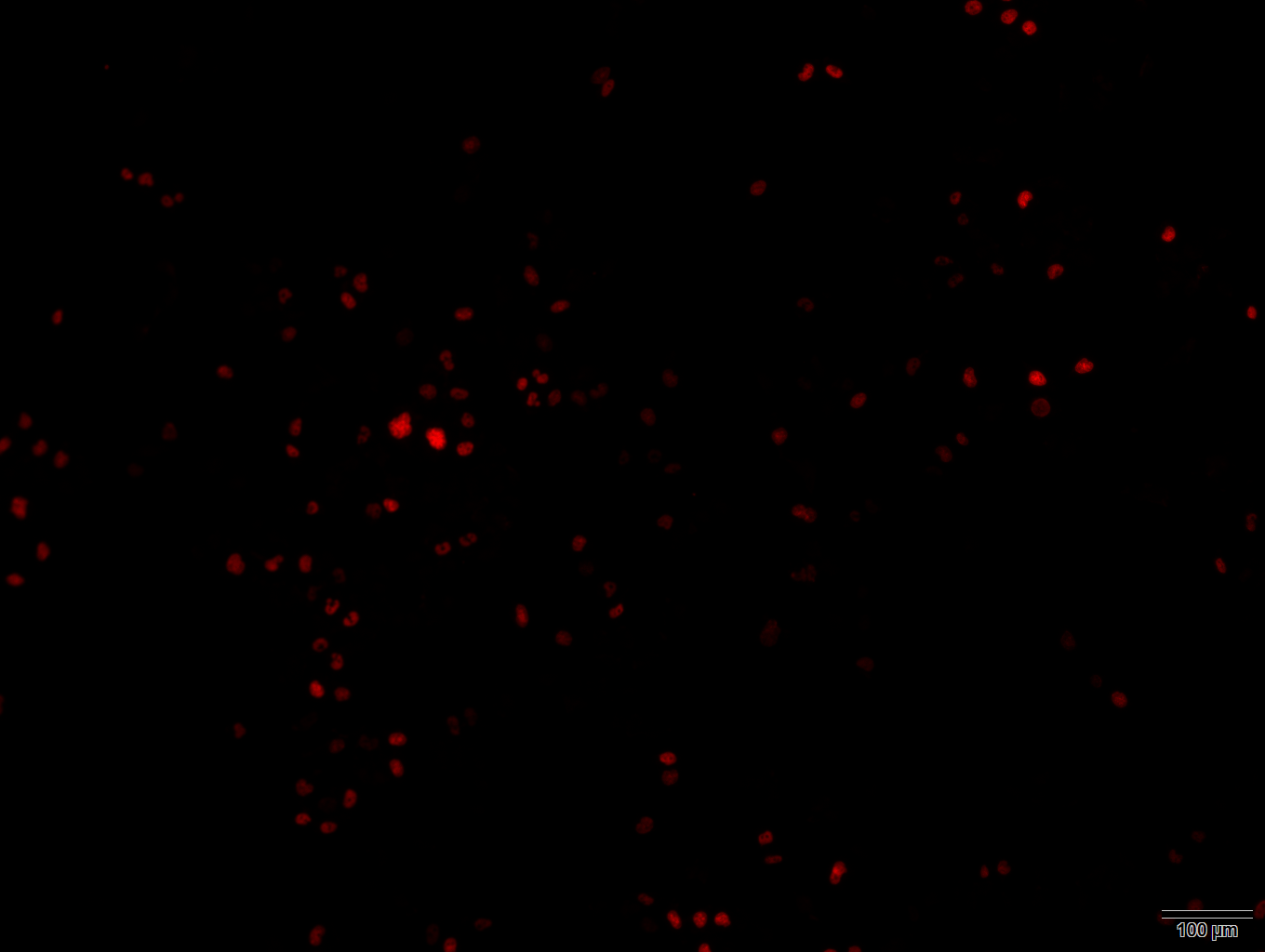

Supplement: S1 File — (ZIP) [file pone.0290753.s001.zip › S2 26.tif]

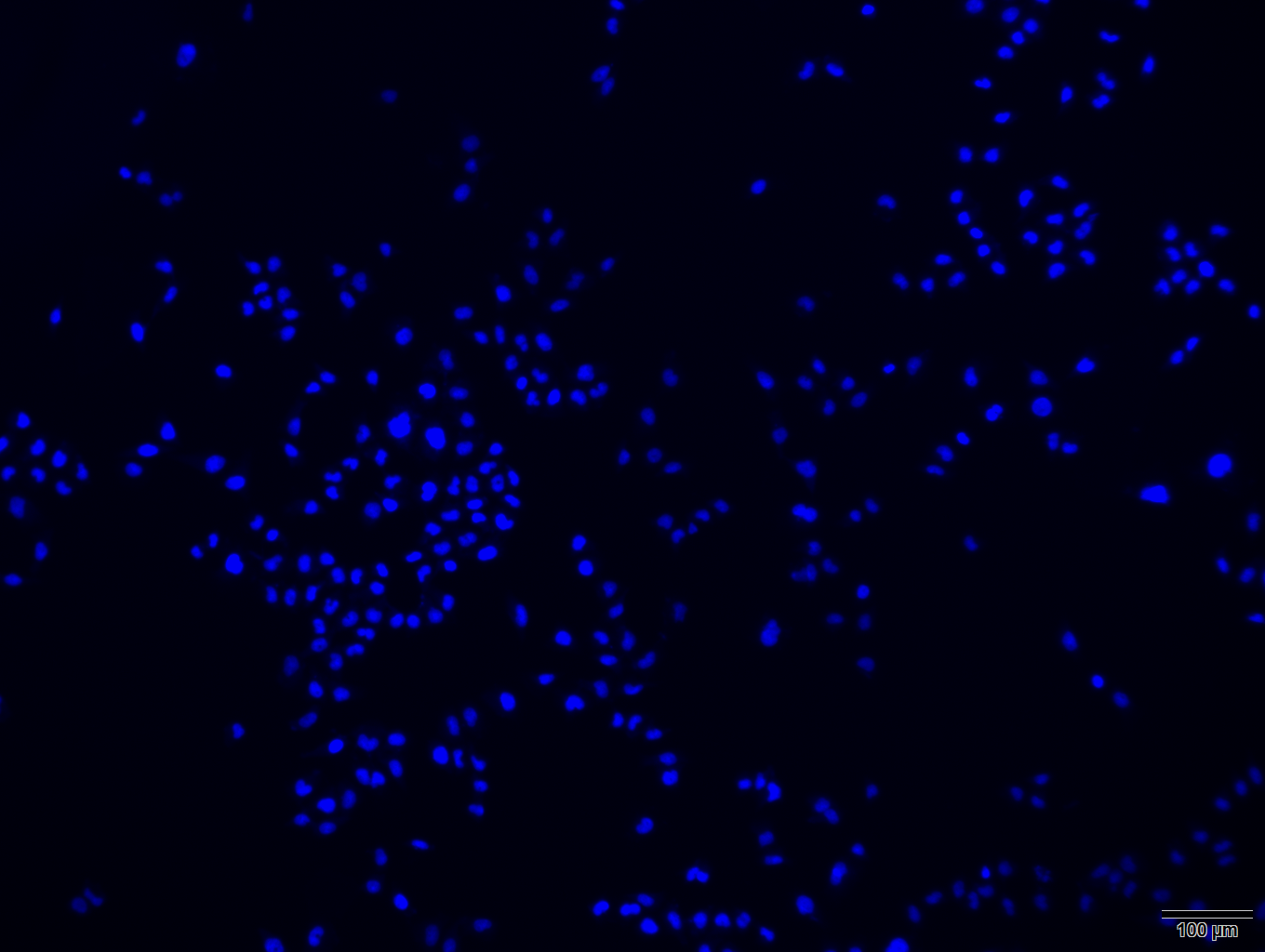

Supplement: S1 File — (ZIP) [file pone.0290753.s001.zip › S2 27.tif]

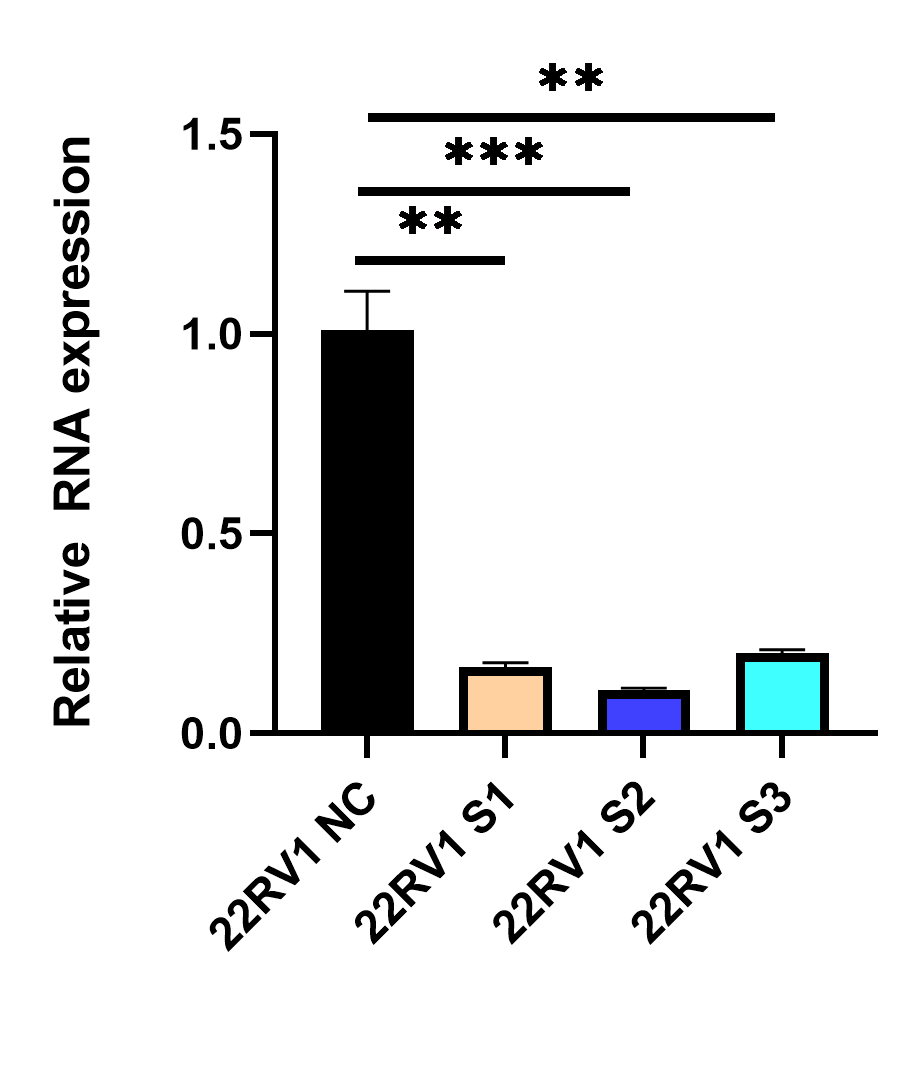

Supplement: S1 File — (ZIP) [file pone.0290753.s001.zip › siRNA knockdown in 22RV1.tif]

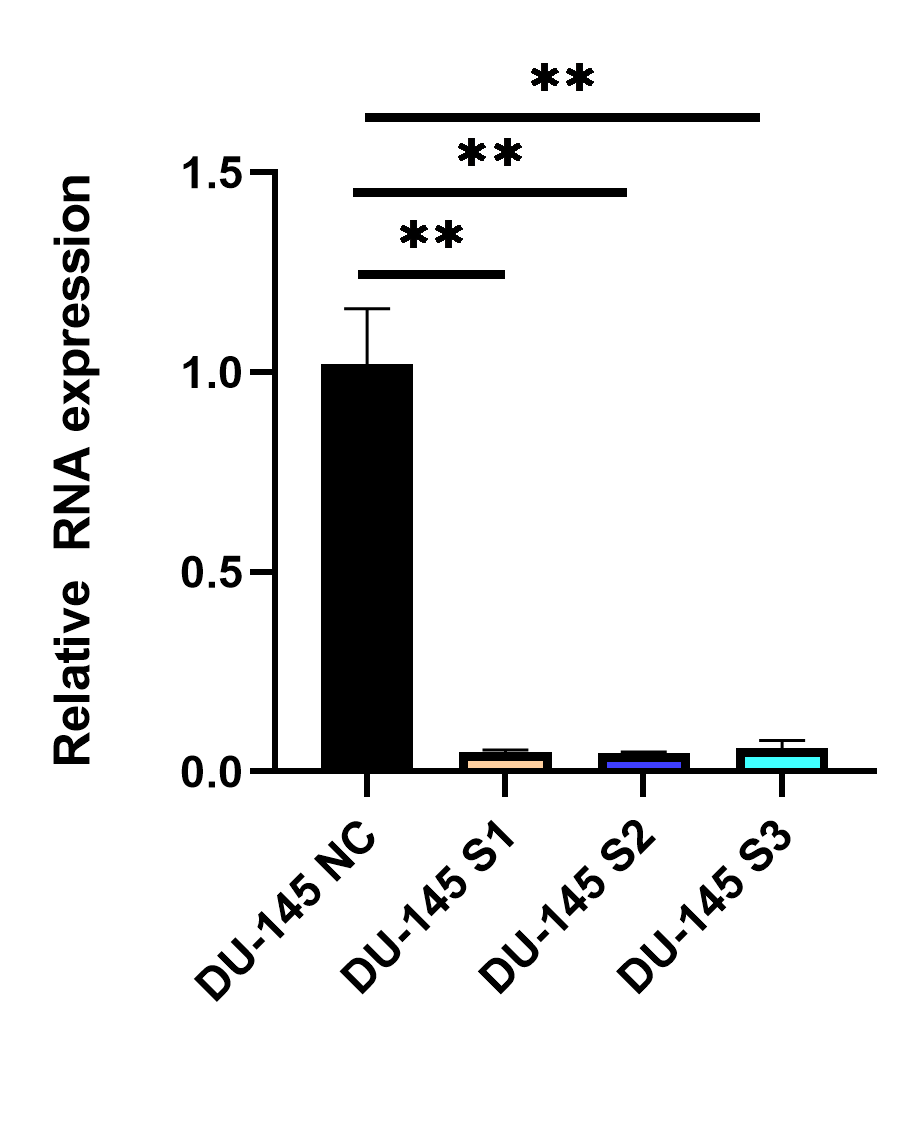

Supplement: S1 File — (ZIP) [file pone.0290753.s001.zip › siRNA knockdown in DU-145.tif]

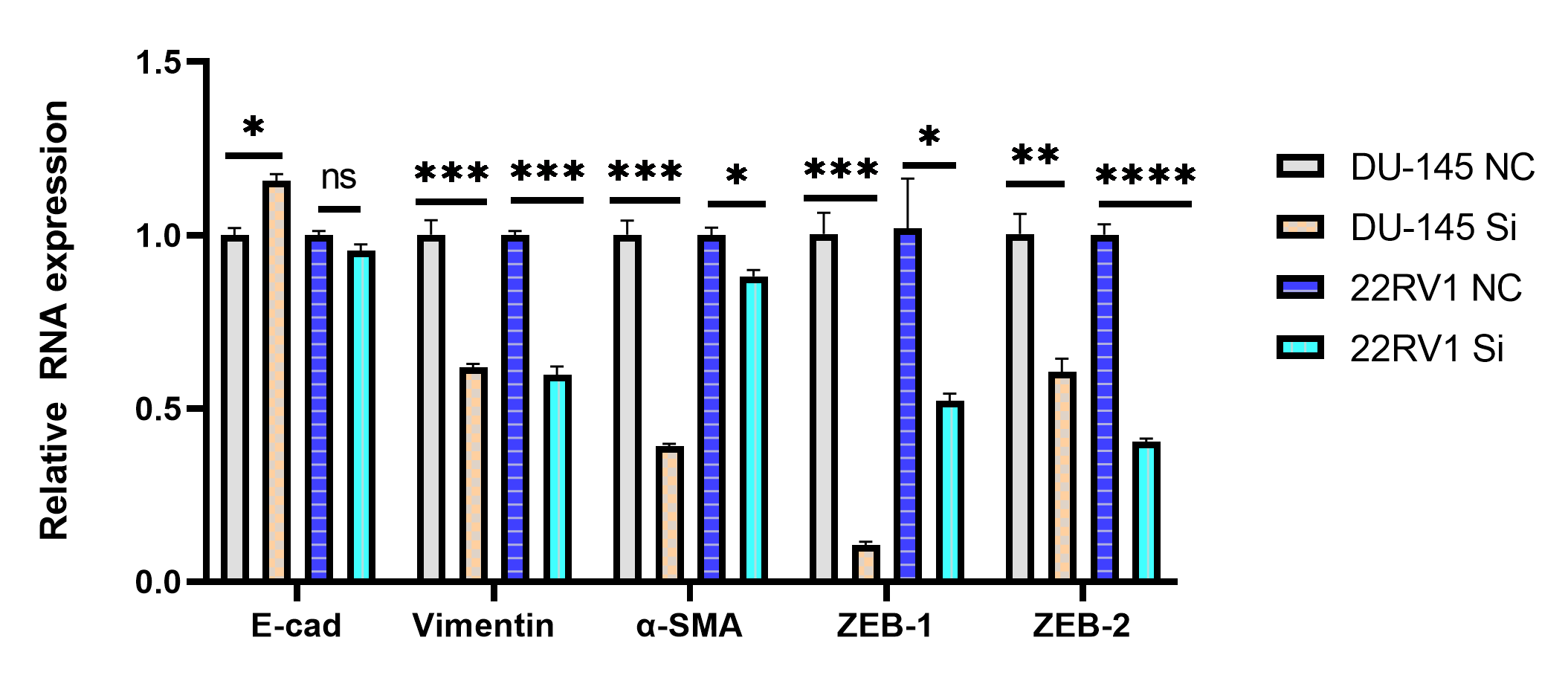

Supplement: S1 File — (ZIP) [file pone.0290753.s001.zip › Statistical analysis of EMT molecular expression.tif]

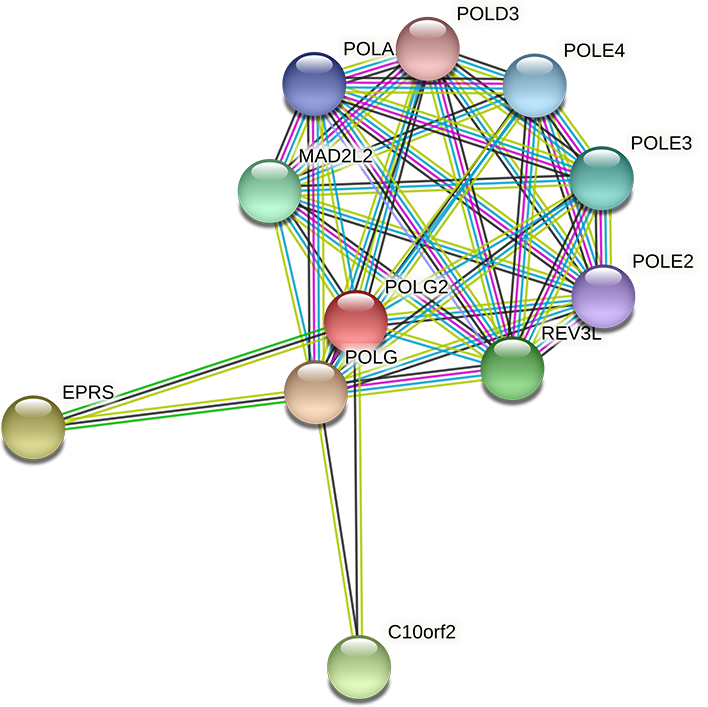

Supplement: S1 File — (ZIP) [file pone.0290753.s001.zip › string_hires_image.tif]

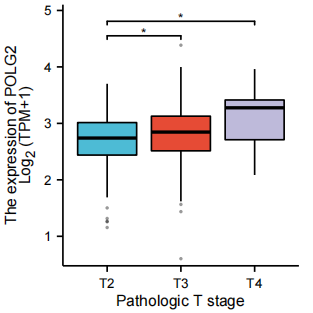

Supplement: S1 File — (ZIP) [file pone.0290753.s001.zip › T stage.tif]

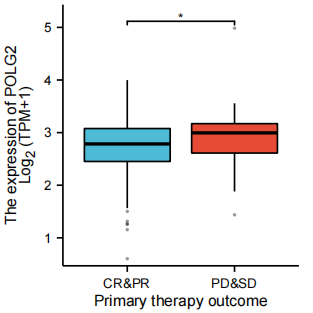

Supplement: S1 File — (ZIP) [file pone.0290753.s001.zip › treatment outcome.tif]
